# Tetrazole and acylsulfonamide bioisosteric replacements of the carboxylic acid in a dual MCL-1/BCL-x_L_ inhibitor are tolerated†

**DOI:** 10.1039/d3ra05711a

**Published:** 2023-11-22

**Authors:** Lijia Chen, Brandon Lowe, Steven Fletcher

**Affiliations:** a University of Maryland School of Pharmacy 20N Pine St Baltimore MD 21201 USA steven.fletcher@rx.umaryland.edu; b University of Maryland Greenebaum Cancer Center 20 S. Greene St. Baltimore MD 21201 USA

## Abstract

Overexpression of the anti-apoptotic protein MCL-1 is associated with a plethora of human cancers, and it reduces the sensitivity of cancer cells to approved chemotherapies. Accordingly, the discovery of MCL-1 inhibitors is an active area of interest. Many inhibitors of the anti-apoptotic MCL-1 protein bear a crucial carboxylic acid that may engage Arg263 in the BH3-binding groove. We previously described the salicylic acid-based dual MCL-1/BCL-x_L_ inhibitor 17cd, which is currently undergoing lead optimization. As part of that process, we wished to investigate bioisosteric replacement of 17cd's key carboxylic acid. Herein we describe the synthesis of a variety of analogues of a simpler analogue of 17cd presenting carboxylic acid surrogates. The acylsulfonamide and tetrazole motifs, which exhibit comparable p*K*_a_s to the carboxylic acid function, displayed similar, or better, binding affinities to MCL-1 and BCL-x_L_ as the corresponding carboxylic acid-containing lead. Our best compound was acylsulfonamide 7d with a *K*_i_ of 800 nM against MCL-1 and 1.82 mM against BCL-x_L_, and demonstrated an improved effect on the viability of the HL60 acute myeloid leukemia cell line relative to the parent carboxylic acid-containing dual inhibitor from which it was derived.

## Abstract 2

Replacement of a key carboxylic acid in a dual MCL-1/BCL-x_L_ inhibitor with bioisosteres was successful, and resulted in the discovery of an acylsulfonamide-derived compound (7d) with the greatest anti-leukemic activity of the entire series.

## Graphical abstract

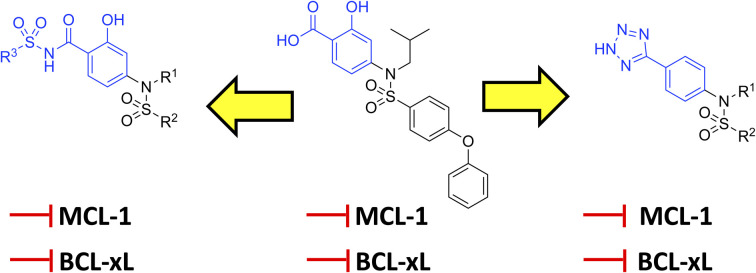

## 1 Introduction

Myeloid cell leukemia-1 (MCL-1) is an anti-apoptotic member of the BCL-2 family of proteins that regulates apoptosis.^1^ Overexpression of MCL-1 is associated with a wide range of human cancers, and protects cancer cells from apoptosis, which inherently also reduces the sensitivity of these cells to standard chemotherapies, as well as to radiotherapy.^2–4^

**Fig. 1:**
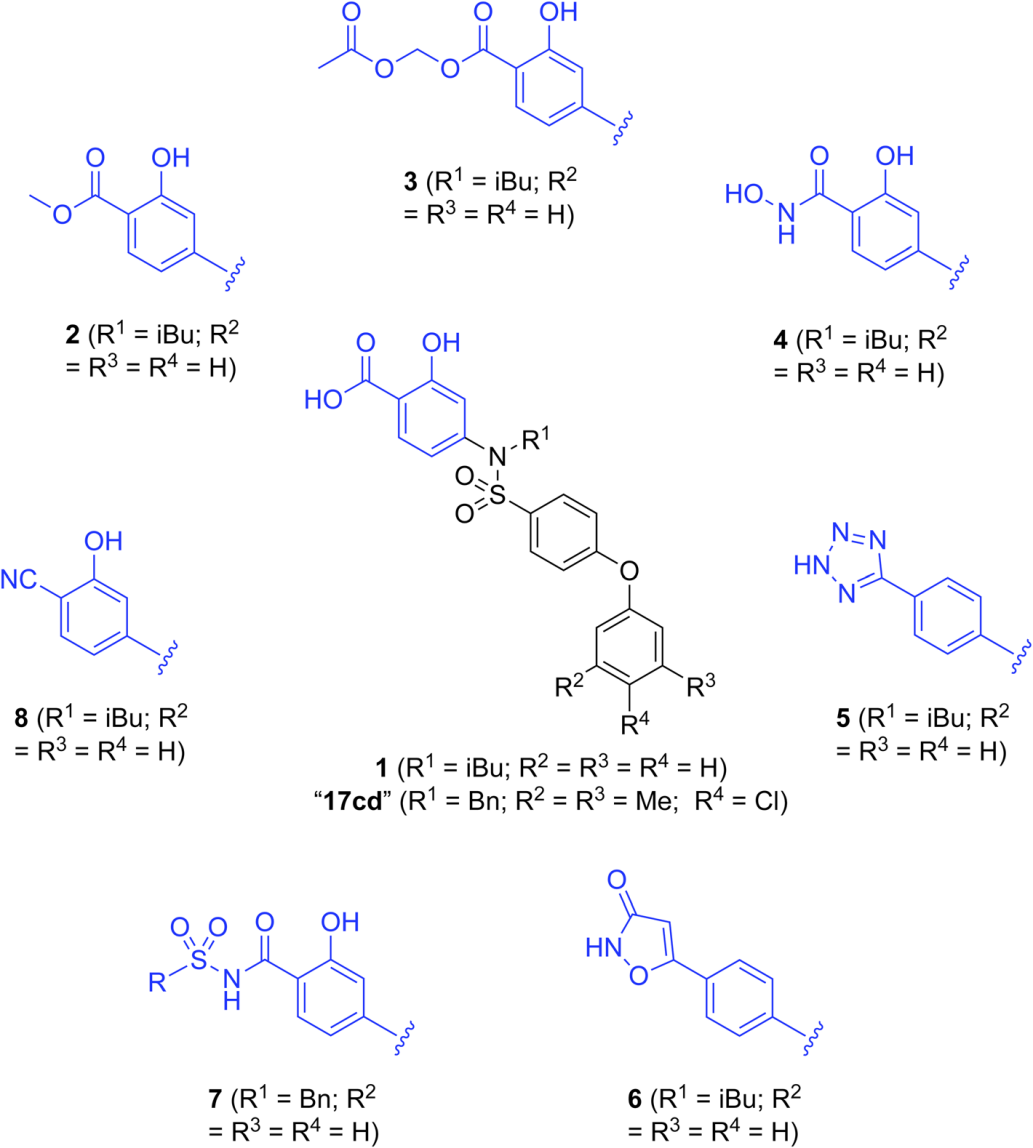
Target esters and bioisosteres of 1 and “17cd”.^11^

Currently, there are several on-going clinical trials investigating the therapeutic efficacy of a variety of MCL-1 inhibitors. However, some trials have been terminated or suspended owing to serious adverse effects.^5^ The majority of MCL-1 inhibitors bind in the BH3-binding groove on the surface of the protein, mimicking the BH3 domain of the pro-apoptotic counterparts, thereby relinquishing these to activate cell death.^5–7^ It is important that research into MCL-1 inhibitors bearing novel chemotypes is continued to increase the likelihood of the discovery of a clinically effective and safe therapeutic. However, selective MCL-1 inhibitors may suffer from resistance due to concomitant anti-apoptotic sister protein upregulation, and *vice versa*.^8,9^ Previously, we described the small molecule “17cd” (Fig. 1) that is a dual inhibitor of MCL-1 (*K*_i_ = 0.629 μM) and the sister anti-apoptotic BCL-2 protein BCL-x_L_ (*K*_i_ = 1.67 μM). We reasoned that a dual MCL-1/BCL-x_L_ inhibitor may be a more promising therapeutic than a selective inhibitor through co-targeting two anti-apoptotic BCL-2 proteins.^10^ However, as predicted, the impact of 17cd on the viability of human leukemia HL60 cells is modest with an IC_50_ of 18.5 μM. We are thus continuing our optimization of 17cd in order to gain greater affinities to both MCL-1 and BCL-x_L_, and that work will be published in due course. The carboxylic acid of 17cd is paramount to its biological activity, and it cannot be discarded,^11^ as has been observed with other carboxylic acid featuring MCL-1 inhibitors^12,13^ that (may) form a salt bridge with Arg263 in the BH3-binding groove on MCL-1.^14^

Since lipophilic drugs containing carboxylic acids poorly penetrate cell membranes and/or become bound to serum proteins, which reduces their bioavailability,^15^ we wanted to investigate if ester prodrugs would enhance cell killing, and if carboxylic acid bioisosteres of 17cd would retain/improve the activity of the parent compound, as well as improve cell killing. Herein, we describe the synthesis and biological evaluation of some ester prodrugs of our salicylic acid-based dual MCL-1/BCL-x_L_ inhibitors as well as some classical and non-classical bioisosteres.

## 2 Experimental

### 2.1 Chemistry

#### 2.1.1 General procedures

All reactions were performed in oven-dried glassware under an inert (N_2_) atmosphere, unless otherwise stated. Anhydrous solvents were used as supplied without further purification. ^1^H and ^13^C NMR spectra were recorded on a Varian 400 MHz NMR spectrometer at 25 °C. Chemical shifts are reported in parts per million (ppm) and are referenced to residual non-deuterated solvent peak (CHCl_3_: *δ*_H_ 7.26, *δ*_C_ 77.2; DMSO: *δ*_H_ 2.50, *δ*_C_ 39.5); coupling constants are in Hz. High resolution mass spectra (HRMS) were recorded on an Aglient 6560 Quadrupole time of flight (QTOF) mass spectrometer using electrospray ionization (ESI).

##### 2.1.1.1 General procedure A: methyl esterification

The acid (1 eq.) was suspended in MeOH (0.5 M), cooled to 0 °C, and H_2_SO_4_ (7 eq.) was added dropwise. The reaction was slowly warmed up to room temperature and then refluxed overnight. TLC indicated the reaction was complete. The volatiles were evaporated and the residue was poured into ice. The pH was adjusted to 7 using 1 M NaOH solution. The precipitate was filtered and washed with deionized water, then dried in the vacuum oven overnight to yield the methyl ester as a white solid.

##### 2.1.1.2 General procedure B: *O*-benzylation

The phenol (1 eq.) was dissolved in anhydrous DMF (0.1 M), cooled to 0 °C, followed by addition of K^*t*^OBu (1.1 eq.). Benzyl bromide (1.1 eq.) was then added and the reaction was stirred at room temperature overnight. TLC indicated the reaction was complete, which was then partitioned between EtOAc and H_2_O. The organic layer was collected, washed repeatedly with H_2_O (4×), then dried over Na_2_SO_4_, filtered, concentrated and purified by flash column chromatography over silica gel using an eluent of Hex/EtOAc 1 : 1 to give the product.

##### 2.1.1.3 General procedure C: reductive amination

The aniline (1 eq.) was dissolved in dichloroethane (0.1 M), then the corresponding aldehyde/ketone (1.3 eq.) and acetic acid (1.2 eq.) were added. Next, NaBH(OAc)_3_ (2.5 eq.) was added batchwise into the reaction and stirred at room temperature overnight. TLC indicated the reaction was complete. Saturated NaHCO_3_ (aq) was poured into the reaction mixture and bubbled for 30 min. The mixture was then collected and partitioned between water and DCM (3×). The combined organic layers were dried over Na_2_SO_4_, filtered, concentrated and purified by flash column chromatography over silica gel using an eluent of Hex/EtOAc 4 : 1 to give the product.

##### 2.1.1.4 General procedure D: sulfonamide synthesis

The aniline (1 eq.) was dissolved in anhydrous CHCl_3_ (0.1 M), followed by the addition of the desired sulfonyl chloride (1.5 eq.), DIPEA (3 eq.) and DMAP (0.1 eq.). The reaction was heated at 65 °C overnight under N_2_ atmosphere. TLC indicated the reaction was complete. The volatiles were evaporated and the residual was reconstituted in EtOAc and washed with 1 M HCl. The organic layer was collected and dried over Na_2_SO_4_, filtered, concentrated and purified by flash column chromatography over silica gel using an eluent of Hex/EtOAc 4 : 1 to give the product.

##### 2.1.1.5 General procedure E: nucleophilic aromatic substitution (S_N_Ar)

The fluorobenzene (1 eq.) was dissolved in anhydrous DMSO (0.1 M), followed by adding corresponding phenol (5 eq.), and K_2_CO_3_ (5 eq.). The reaction was heated at 100 °C overnight. TLC indicated the reaction was complete, which was then partitioned between EtOAc and H_2_O. The organic layer was collected, washed repeatedly with H_2_O (4×), then dried over Na_2_SO_4_, filtered, concentrated and then reconstituted with Et_2_O. The Et_2_O solution was washed with 1 M NaOH (4×), then dried over Na_2_SO_4_, filtered, concentrated and then purified by flash column chromatography over silica gel using an eluent of Hex/EtOAc 4 : 1 to give the product.

##### 2.1.1.6 General procedure F: ester hydrolysis

The ester (1 eq.) was dissolved in a mixed solvent of THF/MeOH/H_2_O 3 : 1 : 1 (0.1 M). LiOH·H_2_O (4 eq.) was added to the reaction, which was stirred at room temperature overnight, or until TLC indicated the reaction was complete. The volatiles were evaporated and the residue was partitioned between EtOAc and 1 M HCl. The organic layer was collected, dried over Na_2_SO_4_, filtered, and concentrated to yield the product.

##### 2.1.1.7 General procedure G: debenzylation

The benzylated phenol (1 eq.) was dissolved in a solvent mixture of toluene/TFA 2 : 1 (0.1 M), and stirred at room temperature overnight. TLC indicated the reaction was complete. The volatiles were evaporated and the residue was purified by preparative TLC using an eluent of DCM/MeOH/AcOH 92 : 7 : 1 to give the product.

##### 2.1.1.8 Methyl 2-hydroxy-4-(*N*-isobutyl-4-phenoxyphenylsulfonamido)benzoate (2)

Methyl 2-(benzyloxy)-4-(*N*-isobutyl-4-phenoxyphenylsulfonamido)benzoate^11^ was debenzylated according to general procedure G on a scale of 0.2 mmol to give the product as a white solid (81 mg, 89%): ^1^H NMR (CDCl_3_, 400 MHz) *δ* 10.78 (1H, s, OH), 7.79 (1H, d, *J* = 8.4 Hz, Ar), 7.51 (2H, d, *J* = 8.4 Hz, Ar), 7.41 (2H, t, *J* = 8.0 Hz, Ar), 7.22 (1H, t, *J* = 8.0 Hz, Ar), 7.07 (2H, d, *J* = 7.6 Hz, Ar), 6.97 (2H, d, *J* = 8.4 Hz, Ar), 6.80 (1H, d, *J* = 8.0 Hz, Ar), 6.64 (1H, s, Ar), 3.95 (3H, s, O-CH_3_), 3.31 (2H, d, *J* = 7.2 Hz, CH_2_), 1.66–1.58 (1H, m, CH), 0.90 (6H, d, *J* = 6.0 Hz, 2*CH_3_); ^13^C NMR (CDCl_3_, 100 MHz) *δ* 169.9, 161.8, 161.6, 155.0, 146.2, 131.5, 130.3, 130.1, 129.7, 124.9, 120.3, 119.7, 117.3, 115.9, 111.3, 57.2, 52.4, 26.8, 19.8.

##### 2.1.1.9 Acetoxymethyl 2-hydroxy-4-(*N*-isobutyl-4-phenoxyphenylsulfonamido)benzoate (3)

Acetoxymethyl 2-(benzyloxy)-4-(*N*-isobutyl-4-phenoxyphenylsulfonamido)benzoate (14) was debenzylated according to general procedure G on a scale of 0.15 mmol to give the product as a white solid (61 mg, 80%): ^1^H NMR (CDCl_3_, 400 MHz) *δ* 10.42 (1H, s, OH), 7.82 (1H, d, *J* = 8.8 Hz, Ar), 7.51 (2H, d, *J* = 8.4 Hz, Ar), 7.41 (2H, t, *J* = 7.6 Hz, Ar), 7.22 (1H, t, *J* = 8.0 Hz, Ar), 7.07 (2H, d, *J* = 8.8 Hz, Ar), 6.97 (2H, d, *J* = 8.4 Hz, Ar), 6.80 (1H, d, *J* = 8.0 Hz, Ar), 6.69 (1H, s, Ar), 5.99 (2H, s, O-C*H*_2_-O), 3.31 (2H, d, *J* = 8.0 Hz, N-CH_2_), 2.15 (3H, s, COCH_3_), 1.65–1.58 (1H, m, CH), 0.90 (6H, d, *J* = 6.4 Hz, 2*CH_3_); ^13^C NMR (CDCl_3_, 100 MHz) *δ* 162.3, 161.7, 147.0, 131.4, 130.7, 130.1, 129.6, 125.0, 120.3, 119.7, 117.3, 116.1, 110.3, 79.3, 57.1, 26.8, 20.6, 19.8; HRMS-ESI: *m*/*z* found 512.1343 [M–H]^−^, C_26_H_26_NO_8_S requires 512.1385.

##### 2.1.1.10 *N*,2-Dihydroxy-4-(*N*-isobutyl-4-phenoxyphenylsulfonamido)benzamide (4)

NH_2_OH·HCl (6.1 mmol, 1.12 g, 25 eq.) and NaOH (36.5 mmol, 1.46 g, 57 eq.) was dissolved in H_2_O (14 mL) in a flask, followed by dropwise addition of methyl 2-hydroxy-4-(*N*-isobutyl-4-phenoxyphenylsulfonamido)benzoate (2; 0.64 mmol, 293 mg, 1 eq.) in dioxane (7 mL). Reaction was stirred at room temperature overnight. TLC indicated the reaction was complete. The reaction was cooled to 0 °C, and 1 M HCl was added slowly. The reaction mixture was partition between DCM and H_2_O, organic layer was collected and dried over Na_2_SO_4_, concentrated, and purified by flash column chromatography over silica gel using an eluent of DCM/MeOH/H_2_O 79 : 9 : 1 to give the product as a light red solid (209 mg, 71%): ^1^H NMR (DMSO-*d*_6_, 400 MHz) *δ* 12.2 (1H, s br, NH), 11.4 (1H, s br, NH-O*H*), 9.34 (1H, s, Ph-O*H*), 7.59 (1H, d, *J* = 7.6 Hz, Ar), 7.52–7.42 (4H, m, Ar), 7.22 (1H, s, Ar), 7.10 (2H, d, *J* = 7.2 Hz, Ar), 7.05 (2H, d, *J* = 7.6 Hz, Ar), 6.63–6.58 (2H, m, Ar), 3.28 (2H, d, *J* = 12.0 Hz, N-CH_2_), 1.44–1.41 (1H, m, Ar), 0.80 (6H, d, *J* = 6.4 Hz, 2*CH_3_); ^13^C NMR (DMSO-*d*_6_, 100 MHz) *δ* 161.3, 159.7, 155.1, 143.5, 131.8, 130.8, 130.2, 128.0, 125.5, 120.5, 118.5, 118.0, 116.9, 114.0, 109.8, 59.8, 25.8, 20.0; HRMS-ESI: *m*/*z* found 455.1323 [M–H]^−^, C_23_H_23_N_2_O_6_S requires 455.1288.

##### 2.1.1.11 *N*-(4-(2*H*-Tetrazol-5-yl)phenyl)-*N*-isobutyl-4-phenoxybenzenesulfonamide (5)

*N*-(4-Cyanophenyl)-*N*-isobutyl-4-phenoxybenzenesulfonamide (18; 0.25 mmol, 100 mg, 1 eq.) was dissolved in anhydrous DMF (0.1 M), followed by the addition of NaN_3_ (0.74 mmol, 49 mg, 3 eq.) and NH_4_Cl (1.0 mmol, 54 mg, 4 eq.). The reaction mixture was heated at 125 °C overnight. TLC indicated the reaction was complete. 1 M HCl was added to the flask to quench the reaction, and the mixture was partitioned between EtOAc and 1 M HCl. Organic layer was collected and washed with 1 M HCl 3 times, dried over Na_2_SO_4_, concentrated, and purified by prep-TLC using an eluent of DCM/MeOH/AcOH 92 : 7 : 1 to give the product as a white solid (86 mg, 76%): ^1^H NMR (CDCl_3_, 400 MHz) *δ* 8.13 (2H, d, *J* = 6.4 Hz, Ar), 7.51 (2H, d, *J* = 8.0 Hz, Ar), 7.39 (2H, t, *J* = 6.8 Hz, Ar), 7.30–7.19 (3H, m, Ar), 7.06 (2H, d, *J* = 6.8 Hz, Ar), 6.97 (2H, d, *J* = 7.6 Hz, Ar), 3.35 (2H, d, *J* = 6.4 Hz, N-CH_2_), 1.62–1.58 (1H, m, CH), 0.90 (6H, d, *J* = 6.0 Hz, 2*CH_3_); ^13^C NMR (CDCl_3_, 100 MHz) *δ* 161.9, 154.8, 142.2, 130.9, 130.2, 129.8, 129.2, 128.1, 125.1, 123.3, 120.4, 117.3, 57.4, 26.8, 19.8; *t*_R_ = 12.3 min (100%); HRMS-ESI: *m*/*z* found 448.1442 [M–H]^−^, C_23_H_22_N_5_O_3_S requires 448.1449.

##### 2.1.1.12 *N*-Isobutyl-*N*-(4-(3-oxo-2,3-dihydroisoxazol-5-yl)phenyl)-4-phenoxybenzenesulfonamide (6)

NH_2_OH·HCl (0.44 mmol, 31 mg, 4 eq.) and NaOH (0.7 mmol, 28 mg, 6.4 eq.) was dissolved in MeOH (2 mL) in a flask, followed by addition of methyl 3-(4-(*N*-isobutyl-4-phenoxyphenylsulfonamido)phenyl)propiolate (19; 0.11 mmol, 50 mg, 1 eq.). Reaction was stirred at room temperature overnight. TLC indicated the reaction was complete. The reaction was cooled to 0 °C, and 1 M HCl was added slowly. The reaction mixture was partition between DCM and H_2_O, organic layer was collected and dried over Na_2_SO_4_, concentrated, and purified by Prep-TLC using an eluent of DCM/MeOH/AcOH 92 : 7 : 1 and then re-purified by Prep-TLC using an eluent of DCM/MeOH/H_2_O 79 : 9 : 1 to give the product as a white solid (10 mg, 20%): ^1^H NMR (CDCl_3_, 400 MHz) *δ* 7.69 (2H, d, *J* = 8.0 Hz, Ar), 7.49 (2H, d, *J* = 8.0 Hz, Ar), 7.42 (2H, t, *J* = 6.8 Hz, Ar), 7.26–7.20 (3H, m, Ar), 7.07 (2H, d, *J* = 7.6 Hz, Ar), 6.98 (2H, d, *J* = 7.6 Hz, Ar), 6.22 (1H, s, C*H*CO), 3.34 (2H, d, *J* = 6.8 Hz, N-CH_2_), 1.62–1.58 (1H, m, CH), 0.92 (6H, d, *J* = 6.4 Hz, 2*CH_3_); ^13^C NMR (CDCl_3_, 100 MHz) *δ* 161.7, 154.9, 141.6, 131.4, 130.9, 130.2, 129.7, 128.9, 128.1, 126.4, 125.0, 120.3, 117.2, 91.8, 57.4, 26.9, 19.8; HRMS-ESI: *m*/*z* found 463.1313 [M–H]^−^, C_25_H_23_N_2_O_5_S requires 463.1333.

##### 2.1.1.13 4-(*N*-Benzyl-4-phenoxyphenylsulfonamido)-2-hydroxy-*N*-(methylsulfonyl)benzamide (7a)

4-(*N*-Benzyl-4-phenoxyphenylsulfonamido)-2-(benzyloxy)-*N*-(methylsulfonyl)benzamide (21a) was debenzylated according to general procedure G on a scale of 0.09 mmol to give the product as a beige solid (25 mg, 50%): ^1^H NMR (DMSO-*d*_6_, 400 MHz) *δ* 7.64–7.58 (3H, m, Ar), 7.45 (2H, t, *J* = 7.6 Hz, Ar), 7.26–7.21 (5H, m, Ar), 7.17–7.08 (5H, m, Ar), 6.58–6.56 (2H, m, Ar), 4.76 (2H, s, N-CH_2_), 3.06 (3H, s, CH_3_); ^13^C NMR (DMSO-*d*_6_, 100 MHz) *δ* 161.6, 154.7, 136.1 131.4, 130.5, 129.9, 128.4, 127.9, 127.5, 125.1, 120.2, 117.6, 117.4, 116.2, 52.8, 40.8.

##### 2.1.1.14 4-(*N*-Benzyl-4-phenoxyphenylsulfonamido)-2-hydroxy-*N*-(phenylsulfonyl)benzamide (7b)

4-(*N*-Benzyl-4-phenoxyphenylsulfonamido)-2-(benzyloxy)-*N*-(phenylsulfonyl)benzamide (21b) was debenzylated according to general procedure G on a scale of 0.08 mmol to give the product as a beige solid (25 mg, 50%); ^1^H NMR (DMSO-*d*_6_, 400 MHz) *δ* 7.85 (2H, d, *J* = 7.2 Hz, Ar), 7.60 (2H, d, *J* = 8.4 Hz, Ar), 7.54–7.42 (6H, m, Ar), 7.25–7.06 (10H, m, Ar), 6.57–6.51 (2H, m, Ar), 4.73 (2H, s, N-CH_2_); ^13^C NMR (DMSO-*d*_6_, 100 MHz) *δ* 161.5, 155.1, 143.3, 136.5, 132.5, 131.7, 130.9, 130.3, 128.9, 128.8, 128.3, 127.8, 127.7, 125.5, 120.6, 118.1, 117.8, 116.5, 53.2; HRMS-ESI: *m*/*z* found 613.1178 [M–H]^−^, C_32_H_25_N_2_O_7_S_2_ requires 613.1109.

##### 2.1.1.15 4-(*N*-Benzyl-4-phenoxyphenylsulfonamido)-2-hydroxy-*N*-((3-nitro-4-(((tetrahydro-2*H*-pyran-4-yl)methyl)amino)phenyl)sulfonyl)benzamide (7c)

4-(*N*-Benzyl-4-phenoxyphenylsulfonamido)-2-(benzyloxy)-*N*-((3-nitro-4-(((tetrahydro-2*H*-pyran-4-yl)methyl)amino)phenyl)sulfonyl)benzamide (21c) was debenzylated according to general procedure G on a scale of 0.08 mmol to give the product as a bright yellow solid (25 mg, 40%): ^1^H NMR (DMSO-*d*_6_, 400 MHz) *δ* 8.61 (1H, t, *J* = 5.6 Hz, N*H*-Ph), 8.57 (1H, s, Ar), 7.90 (1H, d, *J* = 9.6 Hz, Ar), 7.65 (2H, d, *J* = 8.8 Hz, Ar), 7.53–7.47 (3H, m, Ar), 7.29–7.16 (10H, m, Ar), 7.11 (2H, d, *J* = 8.4 Hz, Ar), 6.64 (1H, s, SO_2_N*H*CO), 6.59 (1H, d, *J* = 8.8 Hz, Ar), 4.78 (2H, s, N-CH_2_), 3.85–3.82 (2H, m, CH_2_), 3.34–3.22 (4H, m, 2*CH_2_), 1.91–1.88 (1H, m, CH), 1.60 (2H, d, *J* = 12.0 Hz, CH_2_), 1.29–1.20 (2H, m, CH_2_); ^13^C NMR (DMSO-*d*_6_, 100 MHz) *δ* 161.6, 155.0, 147.4, 143.5, 136.3, 134.8, 131.7, 130.9, 130.5, 130.3, 129.9, 128.8, 128.3, 127.9, 125.5, 120.6, 118.0, 117.8, 116.6, 115.3, 67.0, 53.2, 48.3, 34.2, 30.6; HRMS-ESI: *m*/*z* found 771.1800 [M–H]^−^, C_38_H_35_N_4_O_10_S_2_ requires 771.1693.

##### 2.1.1.16 4-(*N*-Benzyl-4-(4-chloro-3,5-dimethylphenoxy)phenylsulfonamido)-2-hydroxy-*N*-((3-nitro-4-(((tetrahydro-2*H*-pyran-4-yl)methyl)amino)phenyl)sulfonyl)benzamide (7d)

4-(*N*-Benzyl-4-(4-chloro-3,5-dimethylphenoxy)phenylsulfonamido)-2-(benzyloxy)-*N*-((3-nitro-4-(((tetrahydro-2*H*-pyran-4-yl)methyl)amino)phenyl)sulfonyl)benzamide (21d) was debenzylated according to general procedure G on a scale of 0.14 mmol to give the product as a bright yellow solid (65 mg, 56%): ^1^H NMR (DMSO-*d*_6_, 400 MHz) *δ* 8.60–8.51 (2H, m, Ph-N*H* and Ar), 7.85 (1H, d, *J* = 8.8 Hz, Ar), 7.59 (2H, d, *J* = 7.6 Hz, Ar), 7.47 (1H, d, *J* = 8.0 Hz, Ar), 7.20–7.06 (8H, m, Ar), 6.99 (2H, s, Ar), 6.56–6.51 (2H, m, Ar), 4.73 (2H, s, N-CH_2_), 3.79 (2H, d, *J* = 9.6 Hz, CH_2_), 3.28–3.18 (4H, m, 2*CH_2_), 2.30 (6H, s, 2*CH_3_), 1.87–1.84 (1H, m, CH), 1.56 (2H, d, *J* = 12.4 Hz, CH_2_), 1.21–1.18 (2H, m, CH_2_); ^13^C NMR (DMSO-*d*_6_, 100 MHz) *δ* 161.4, 153.0, 147.3, 143.3, 138.4, 136.5, 134.8, 131.8, 130.3, 129.8, 128.8, 128.3, 127.8, 120.7, 118.1, 117.8, 116.5, 115.1, 67.0, 53.2, 48.3, 34.2, 30.6, 20.8; HRMS-ESI: *m*/*z* found 833.1383 [M–H]^−^, C_40_H_38_ClN_4_O_10_S_2_ requires 833.1723.

##### 2.1.1.17 *N*-(4-Cyano-3-hydroxyphenyl)-*N*-isobutyl-4-phenoxybenzenesulfonamide (8)

*N*-(4-Cyano-3-methoxyphenyl)-*N*-isobutyl-4-phenoxybenzenesulfonamide (24; 0.07 mmol, 30 mg, 1 eq.) was dissolved in anhydrous DMF (0.1 M), followed by the addition of LiCl (0.28 mmol, 12 mg, 4 eq.). The reaction mixture was then stirred at 160 °C for 30 min in a microwave vessel. The reaction was allowed to cool, dilute with EtOAc and partition with between EtOAc and H_2_O. The organic layers were combined and washed with H_2_O, dried over Na_2_SO_4_, concentrated, and purified by flash column chromatography over silica gel using an eluent of Hex/EtOAc 4 : 1 to give the product as a white solid (11 mg, 40%): ^1^H NMR (CDCl_3_, 400 MHz) *δ* 7.50 (2H, d, *J* = 8.0 Hz, Ar), 7.42 (3H, t, *J* = 9.2 Hz, Ar), 7.23 (1H, t, *J* = 7.2 Hz, Ar), 7.06 (2H, d, *J* = 7.6 Hz, Ar), 7.01–6.97 (3H, m, Ar), 6.60 (1H, d, *J* = 7.6 Hz, Ar), 3.29 (2H, d, *J* = 6.8 Hz, Ar), 1.61–1.56 (1H, m, CH), 0.89 (6H, d, *J* = 6.0 Hz, 2*CH_3_); ^13^C NMR (CDCl_3_, 100 MHz) *δ* 162.0, 159.3, 154.7, 145.0, 133.2, 130.7, 130.2, 129.7, 125.2, 120.4, 119.1, 117.3, 117.2, 115.8, 98.8, 57.1, 26.8, 19.8; *t*_R_ = 13.3 min (100%); HRMS-ESI: *m*/*z* found 421.1261 [M–H]^−^, C_23_H_21_N_4_O_10_S_2_ requires 421.1228.

##### 2.1.1.18 Methyl 4-(*N*-benzyl-4-phenoxyphenylsulfonamido)-2-(benzyloxy)benzoate (11)

4-Amino-2-hydroxybenzoic acid was esterified according to general procedure A on a scale of 26 mmol to give methyl 4-amino-2-hydroxybenzoate as a brown solid (4.1 g, 95%): ^1^H NMR (CDCl_3_, 400 MHz) *δ* 10.92 (1H, s, O*H*), 7.59 (1H, d, *J* = 7.6 Hz, Ar), 6.13 (1H, d, *J* = 7.6 Hz, Ar), 6.11 (1H, s, Ar), 3.85 (3H, s, OC*H*_3_); ^13^C NMR (CDCl_3_, 100 MHz) *δ* 170.5, 163.5, 153.3, 131.6, 106.8, 103.0, 100.7, 51.7; methyl 4-amino-2-hydroxybenzoate was *O*-benzylated according to general procedure B on a scale of 12 mmol to give Methyl 4-amino-2-(benzyloxy)benzoate as a beige solid (2.1 g, 70%): ^1^H NMR (CDCl_3_, 400 MHz) *δ* 7.78 (1H, d, *J* = 8.8 Hz, Ar), 7.55–7.46 (2H, m, Ar), 7.37 (2H, t, *J* = 7.2 Hz, Ar), 7.30 (1H, t, *J* = 7.2 Hz, Ar), 6.35–6.24 (2H, m, Ar), 5.12 (2H, s, C*H*_2_), 4.02 (2H, br s, N*H*_2_), 3.83 (3H, s, OC*H*_3_); ^13^C NMR (CDCl_3_, 100 MHz) *δ* 161.6, 155.9, 147.1, 132.1, 129.6, 123.8, 122.9, 121.9, 102.1, 94.7, 65.6, 46.7. Methyl 4-amino-2-(benzyloxy)benzoate underwent reductive amination with benzaldehyde according to general procedure C on a scale of 3.0 mmol to give methyl 4-(benzylamino)-2-(benzyloxy)benzoate as a pale brown solid (500 mg, 48%): ^1^H NMR (CDCl_3_, 400 MHz) *δ* 7.77 (1H, d, *J* = 8.4 Hz, Ar), 7.46 (2H, d, *J* = 8.0 Hz, Ar), 7.37–7.24 (7H, m, Ar), 6.19 (1H, d, *J* = 8.4 Hz, Ar), 6.14 (1H, s, Ar), 5.26 (2H, s, O-CH_2_), 4.46 (1H, s, NH), 4.31 (2H, s, N-CH_2_), 3.82 (3H, s, O-CH_3_); ^13^C NMR (CDCl_3_, 100 MHz) *δ* 166.4, 160.7, 152.8, 138.2, 136.9, 134.2, 128.8, 128.5, 127.6, 127.5, 127.4, 126.7, 108.6, 104.9, 97.4, 70.3, 51.4, 47.7. Methyl 4-(benzylamino)-2-(benzyloxy)benzoate was coupled to 4-phenoxybenzenesulfonyl chloride according to general procedure D on a scale of 1.44 mmol to yield the product as a beige solid (800 mg, 96%): ^1^H NMR (CDCl_3_, 400 MHz) *δ* 7.63 (1H, d, *J* = 8.4 Hz, Ar), 7.55 (2H, d, *J* = 8.4 Hz, Ar), 7.42–7.34 (6H, m, Ar), 7.30 (1H, d, *J* = 6.4 Hz, Ar), 7.22–7.16 (4H, m, Ar), 7.12–7.10 (2H, m, Ar), 7.05 (2H, d, *J* = 8.0 Hz, Ar), 6.98 (2H, d, *J* = 8.8 Hz, Ar), 6.75 (1H, s, Ar), 6.58 (1H, d, *J* = 7.6 Hz, Ar), 4.98 (2H, s, O-CH_2_), 4.68 (2H, s, N-CH_2_), 3.83 (3H, s, O-CH_3_); ^13^C NMR (CDCl_3_, 100 MHz) *δ* 166.0, 161.9, 158.2, 154.8, 143.5, 136.2, 135.2, 132.0, 131.5, 130.3, 129.9, 128.6, 128.5, 128.4, 127.9, 127.8, 126.8, 125.1, 120.4, 119.6, 119.3, 117.3, 114.8, 70.6, 54.1, 52.1.

##### 2.1.1.19 4-(*N*-Benzyl-4-phenoxyphenylsulfonamido)-2-(benzyloxy)benzoic acid (13)

Methyl 4-(*N*-benzyl-4-phenoxyphenylsulfonamido)-2-(benzyloxy)benzoate was saponified according to general procedure F on a scale of 1.38 mmol to give the product as a beige solid (450 mg, 57%): ^1^H NMR (CDCl_3_, 400 MHz) *δ* 10.5 (1H, s br, COOH), 7.97 (1H, d, *J* = 8.8 Hz, Ar), 7.54 (2H, d, *J* = 8.4 Hz, Ar), 7.44–7.34 (6H, m, Ar), 7.24–7.15 (6H, m, Ar), 7.08–7.05 (3H, m, Ar), 6.99 (2H, d, *J* = 8.4 Hz, Ar), 6.64 (1H, d, *J* = 8.8 Hz, Ar), 5.14 (2H, s, O-CH_2_), 4.72 (2H, s, N-CH_2_); ^13^C NMR (CDCl_3_, 100 MHz) *δ* 164.5, 162.2, 157.1, 154.7, 144.9, 134.9, 133.9, 131.0, 130.3, 129.8, 129.3, 129.2, 128.6, 128.3, 128.1, 127.9, 125.3, 120.5, 119.7, 117.4, 116.8, 114.8, 72.5, 53.8.

##### 2.1.1.20 Acetoxymethyl 2-(benzyloxy)-4-(*N*-isobutyl-4-phenoxyphenylsulfonamido)benzoate (14)

2-(Benzyloxy)-4-(*N*-isobutyl-4-phenoxyphenylsulfonamido)benzoic acid^11^ (0.22 mmol, 116 mg, 1 eq.) was dissolved in anhydrous DMF (0.1 M),followed by the addition of K_2_CO_3_ (0.44 mmol, 61 mg, 2 eq.), bromomethyl acetate (0.22 mmol, 22 μL, 1 eq.). The reaction was stirred at room temperature overnight. TLC indicated the reaction was completed. The mixture was then partitioned between water (50 mL) and EtOAc (3 × 50 mL). Organic layer was combined, washed with H_2_O, dried over Na_2_SO_4_, filtered, concentrated and purified by flash column chromatography over silica gel using an eluent of Hex/EtOAc 4 : 1 to give product as white solid (91 mg, 68%); ^1^H NMR (CDCl_3_, 400 MHz) *δ* 7.78 (1H, d, *J* = 8.0 Hz, Ar), 7.45 (4H, d, *J* = 8.4 Hz, Ar), 7.40–7.34 (4H, m, Ar), 7.24 (1H, t, *J* = 7.2 Hz, Ar), 7.20 (1H, t, *J* = 6.8 Hz, Ar), 7.03 (2H, d, *J* = 7.6 Hz, Ar), 6.95–6.90 (3H, m, Ar), 6.58 (1H, d, *J* = 8.4 Hz, Ar), 5.94 (2H, s, O-C*H*_2_-O), 5.10 (2H, s, O-CH_2_-Ph), 3.26 (2H, d, *J* = 6.8 Hz, N-CH_2_), 2.07 (3H, s, COCH_3_), 1.52–1.45 (1H, m, CH), 0.83 (6H, d, *J* = 6.4 Hz, 2*CH_3_); ^13^C NMR (CDCl_3_, 100 MHz) *δ* 163.7, 161.7, 159.0, 154.9, 145.0, 135.9, 132.6, 131.3, 130.2, 129.7, 128.6, 127.9, 126.9, 125.1, 120.3, 118.8, 117.9, 117.2, 114.8, 79.5, 70.7, 57.3 26.8, 20.7, 19.8.

##### 2.1.1.21 *N*-(4-Iodophenyl)-*N*-isobutyl-4-phenoxybenzenesulfonamide (17)

4-Iodo-*N*-isobutylaniline was coupled to 4-phenoxybenzenesulfonyl chloride according to general procedure D on a scale of 0.73 mmol to give the product as a beige solid (100 mg, 27%): ^1^H NMR (CDCl_3_, 400 MHz) *δ* 7.61 (2H, d, *J* = 8.8 Hz, Ar), 7.47 (2H, d, *J* = 8.8 Hz, Ar), 7.39 (2H, t, *J* = 7.6 Hz, Ar), 7.20 (1H, t, *J* = 8.0 Hz, Ar), 7.04 (2H, d, *J* = 8.0 Hz, Ar), 6.95 (2H, d, *J* = 8.8 Hz, Ar), 6.81 (2H, d, *J* = 8.4 Hz, Ar), 3.25 (2H, d, *J* = 7.2 Hz, N-CH_2_), 1.55–1.52 (1H, m, CH), 0.87 (6H, d, *J* = 6.8 Hz, 2*CH_3_); ^13^C NMR (CDCl_3_, 100 MHz) *δ* 161.6, 154.9, 139.3, 138.2, 131.4, 130.3, 130.1, 129.8, 125.0, 120.3, 117.2, 93.1, 57.5, 26.8, 19.8.

##### 2.1.1.22 *N*-(4-Cyanophenyl)-*N*-isobutyl-4-phenoxybenzenesulfonamide (18)

A stirring solution of *N*-(4-iodophenyl)-*N*-isobutyl-4-phenoxybenzenesulfonamide (17; 0.6 mmol, 300 mg, 1 eq.) in anhydrous DMF (0.1 M) was degassed, and then Zn(CN)_2_ (0.6 mmol, 70 mg, 1 eq.), Pd(PPh_3_)_4_ (0.3 mmol, 347 mg, 0.5 eq.), Zn (0.03 mmol, 2 mg, 0.05 eq.) and Zn(OAc)_2_ (0.03 mmol, 6 mg, 0.05 eq.) were added. The reaction was heated to 120 °C for 2 h. After which reaction was allowed to cool, add water, extract with EtOAc 3 times. The organic layers were combined and washed with NaHCO_3_ (aq), dried over Na_2_SO_4_, concentrated, and purified by flash column chromatography over silica gel using an eluent of Hex/EtOAc 6 : 1 to give the product as a brown solid (100 mg, 41%): ^1^H NMR (CDCl_3_, 400 MHz) *δ* 7.60 (2H, d, *J* = 7.6 Hz, Ar), 7.44–7.38 (4H, m, Ar), 7.22 (3H, d, *J* = 8.4 Hz, Ar), 7.04 (2H, d, *J* = 8.0 Hz, Ar), 6.95 (2H, d, *J* = 7.6 Hz, Ar), 3.32 (2H, d, *J* = 7.2 Hz, N-CH_2_), 1.60–1.51 (1H, m, CH), 0.88 (6H, d, *J* = 6.4 Hz, 2*CH_3_); ^13^C NMR (CDCl_3_, 100 MHz) *δ* 161.9, 143.8, 132.8, 130.9, 130.2, 129.6, 128.7, 125.1, 120.4, 118.1, 117.3, 111.2, 57.1, 26.9, 19.8.

##### 2.1.1.23 Methyl 3-(4-(*N*-isobutyl-4-phenoxyphenylsulfonamido)phenyl)propiolate (19)

*N*-(4-Iodophenyl)-*N*-isobutyl-4-phenoxybenzenesulfonamide (17; 0.6 mmol, 300 mg, 1 eq.) was dissolved in anhydrous toluene (0.1 M), followed by PPh_3_ (0.06 mmol, 15.7 mg, 0.1 eq.), CuI (0.24 mmol, 46 mg, 0.4 mmol) and Et_3_N (4.8 mmol, 669 μL, 8 eq.). The system was flushed with N_2_ for 5 min, then Pd(PPh_3_)_2_Cl_2_ (0.12 mmol, 84 mg, 0.2 eq.) and methyl propionate (2.4 mmol, 213.5 μL, 4 eq.) was added. The reaction was stirred in a microwave reactor at 110 °C fir 40 min with high absorption level, then cooled to room temperature and stirred overnight. TLC indicated the reaction was complete. The reaction mixture was partitioned between H_2_O and DCM 3 times, organic layer was collected and dried over Na_2_SO_4_, concentrated, and purified by flash column chromatography over silica gel using an eluent of hexanes/ethyl acetate 4 : 1 to give the product as a yellow oil (124 mg, 44%): ^1^H NMR (CDCl_3_, 400 MHz) *δ* 7.53 (2H, d, *J* = 8.4 Hz, Ar), 7.47–7.39 (4H, m, Ar), 7.24 (1H, t, *J* = 8.0 Hz, Ar), 7.12 (2H, d, *J* = 8.8 Hz, Ar), 7.06 (2H, d, *J* = 6.8 Hz, Ar), 6.96 (2H, d, *J* = 8.4 Hz, Ar), 3.04 (3H, s, O-CH_3_), 3.31 (2H, d, *J* = 8.0 Hz, N-CH_2_), 1.59–1.55 (1H, m, CH), 0.89 (6H, d, *J* = 6.0 Hz, 2*CH_3_); ^13^C NMR (CDCl_3_, 100 MHz) *δ* 161.7, 154.9, 154.3, 141.6, 133.6, 131.2, 130.2, 129.7, 128.4, 125.1, 120.3, 118.7, 117.2, 85.4, 81.1, 57.3, 52.9, 26.8, 19.8.

##### 2.1.1.24 4-Fluoro-3-nitrobenzenesulfonamide

2-fluoronitrobenzene (35 mmol, 5 g, 1 eq.) was dissolved in chlorosulfonic acid (11 mL) and heated at 95 °C overnight. The reaction mixture was cooled to room temperature, whilst 110 mL isopropanol and 30 mL NH_3_·H_2_O was prepared in a round bottom flask and cooled to −40 °C. The reaction mixture was slowly added into the flask over 1 h, which formed yellow slurry. Upon the completion of addition, TLC indicated the reaction was complete. 3 M HCl was added to the reaction till pH was acidic. The mixture was partitioned between H_2_O and EtOAc 3 times, organic layer was collected and combined, dried over Na_2_SO_4_, concentrated then reconstituted with H_2_O to reform the slurry, filtered, and washed with small amount of H_2_O, filter cake was collected and dried in an vacuum oven overnight to yield the product as a beige solid (1.7 g, 22%); ^1^H NMR (DMSO-*d*_6_, 400 MHz) *δ* 8.50 (1H, d, *J* = 7.2 Hz, Ar), 8.18–8.15 (1H, m, Ar), 7.81–7.76 (1H, m, Ar), 7.71 (2H, s, NH_2_); ^13^C NMR (DMSO-*d*_6_, 100 MHz) *δ* 158.0, 155.4, 141.3, 133.9, 133.8, 124.5, 120.5, 120.3.

##### 2.1.1.25 3-Nitro-4-(((tetrahydro-2*H*-pyran-4-yl)methyl)amino)benzenesulfonamide (20)

4-Fluoro-3-nitrobenzenesulfonamide (7.75 mmol, 1.7 g, 1 eq.) was dissolved in THF (0.1 M) followed by TEA (9.3 mmol, 1.3 mL, 1.2 eq.) and 1-tetrahydropyran-4-yl-methylamine (7.75 mmol, 892 mg, 1 eq.). The reaction was stirred at room temperature overnight. TLC indicated the reaction was complete. The reaction was diluted with EtOAc, then washed the organic layer with saturated NaH_2_PO_4_ solution, brine, then dried over dried over Na_2_SO_4_, concentrated and purified by flash column chromatography over silica gel using an eluent of hexanes/ethyl acetate, 1 : 2 to give the product as a bright yellow solid (2.1 g, 90%); ^1^H NMR (DMSO-*d*_6_, 400 MHz) *δ* 8.55 (1H, t, *J* = 5.2 Hz, NH), 8.42 (1H, d, *J* = 1.2 Hz, Ar), 7.78 (1H, d, *J* = 9.2 Hz, Ar), 7.31 (2H, s, NH_2_), 7.26 (1H, d, *J* = 8.4 Hz, Ar),3.83–3.79 (2H, m, NH-C*H*_2_), 3.33–3.19 (4H, m, 2*CH_2_), 1.88–1.585 (1H, m, CH), 1.57 (2H, d, *J* = 12.4 Hz, CH_2_), 1.26–1.17 (2H, m, CH_2_); ^13^C NMR (DMSO-*d*_6_, 100 MHz) *δ* 147.1, 133.2, 130.4, 129.8, 125.2, 116.2, 67.1, 48.2, 39.3, 30.6.

##### 2.1.1.26 4-(*N*-Benzyl-4-phenoxyphenylsulfonamido)-2-(benzyloxy)-*N*-(methylsulfonyl)benzamide (21a)

4-(*N*-Benzyl-4-phenoxyphenylsulfonamido)-2-(benzyloxy)benzoic acid was coupled to methanesulfonamide according to general procedure H on a scale of 0.18 mmol to give the product as a beige solid (56 mg, 56%): ^1^H NMR (CDCl_3_, 400 MHz) *δ* 10.14 (1H, s, NH), 8.01 (1H, d, *J* = 8.8 Hz, Ar), 7.56 (2H, d, *J* = 8.4 Hz, Ar), 7.45–7.36 (7H, m, Ar) 7.26–7.17 (6H, m, Ar), 7.08 (2H, d, *J* = 8.0 Hz, Ar), 7.03–7.00 (3H, m, Ar), 6.68 (1H, d, *J* = 8.0 Hz, Ar), 5.12 (2H, s, O-CH_2_), 4.76 (2H, s, N-CH_2_), 3.27 (3H, s, CH_3_); ^13^C NMR (CDCl_3_, 100 MHz) *δ* 162.6, 162.2, 157.0, 154.7, 145.3, 134.9, 133.9, 132.9, 131.1, 130.3, 129.8, 129.2, 128.3, 127.9, 127.8, 125.3, 120.5, 119.6, 117.7, 117.4, 114.7, 72.3, 53.8, 41.6.

##### 2.1.1.27 4-(*N*-Benzyl-4-phenoxyphenylsulfonamido)-2-(benzyloxy)-*N*-(phenylsulfonyl)benzamide (21b)

4-(*N*-benzyl-4-phenoxyphenylsulfonamido)-2-(benzyloxy)benzoic acid (13) was coupled to benzenesulfonamide according to general procedure H on a scale of 0.18 mmol to give the product as a beige solid (55 mg, 44%): ^1^H NMR (CDCl_3_, 400 MHz) *δ* 10.24 (1H, s, NH), 7.93 (2H, d, *J* = 8.0 Hz, Ar), 7.84 (1H, d, *J* = 8.0 Hz, Ar), 7.56 (1H, t, *J* = 8.0 Hz, Ar), 7.52–7.38 (10H, m, Ar), 7.21–7.13 (7H, m, Ar), 7.05 (2H, d, *J* = 8.0 Hz, Ar), 6.98–6.95 (3H, m, Ar), 6.57 (1H, d, *J* = 8.8 Hz, Ar), 5.09 (2H, s, O-CH_2_), 4.70 (2H, s, N-CH_2_); ^13^C NMR (CDCl_3_, 100 MHz) *δ* 184.0, 178.4, 162.1, 161.5, 156.8, 144.9, 138.8, 134.9, 134.1, 133.7, 132.8, 130.9, 130.3, 129.7, 129.2, 128.8, 128.6, 128.4, 128.3, 128.0, 127.9, 125.3, 120.5, 119.3, 118.1, 117.3, 114.8, 72.2, 53.8.

##### 2.1.1.28 4-(*N*-Benzyl-4-phenoxyphenylsulfonamido)-2-(benzyloxy)-*N*-((3-nitro-4-(((tetrahydro-2*H*-pyran-4-yl)methyl)amino)phenyl)sulfonyl)benzamide (21c)

4-(*N*-Benzyl-4-phenoxyphenylsulfonamido)-2-(benzyloxy)benzoic acid (13) was coupled to 3-nitro-4-(((tetrahydro-2*H*-pyran-4-yl)methyl)amino)benzenesulfonamide (20) according to general procedure H on a scale of 0.27 mmol to give the product as bright yellow solid (66 mg, 30%): ^1^H NMR (CDCl_3_, 400 MHz) *δ* 10.28 (1H, s, SO_2_N*H*), 8.73 (1H, s, N*H*CH_2_), 8.49 (1H, t, *J* = 5.2 Hz, Ar), 8.01 (1H, d, *J* = 8.8 Hz, Ar), 7.84 (1H, d, *J* = 8.0 Hz, Ar), 7.52–7.38 (8H, m, Ar), 7.24–7.12 (7H, m, Ar), 7.05 (2H, d, *J* = 7.6 Hz, Ar), 6.97–6.95 (3H, m, Ar), 6.85 (1H, d, *J* = 10.0 Hz, Ar), 6.59 (1H, d, *J* = 8.8 Hz, Ar), 5.09 (2H, s, O-CH_2_), 4.70 (2H, s, N-CH_2_), 4.02–3.98 (2H, m, CH_2_), 3.38 (2H, t, *J* = 12.4 Hz, CH_2_), 3.23 (2H, t, *J* = 6.0 Hz, CH_2_), 1.93–1.90 (1H, m, CH), 1.70 (2H, d, *J* = 12.4 Hz, CH_2_), 1.45–1.35 (2H, m, CH_2_); ^13^C NMR (CDCl_3_, 100 MHz) *δ* 162.1, 161.7, 156.9, 154.6, 147.9, 145.1, 135.5, 134.9, 133.9, 132.7, 130.9, 130.3, 129.7, 129.4, 129.3, 129.1, 128.6, 128.3, 127.9, 125.3, 124.8, 120.5, 119.4, 117.8, 117.3, 114.6, 113.7, 72.3, 67.4, 53.8, 49.1, 34.6, 30.7.

##### 2.1.1.29 4-(*N*-Benzyl-4-(4-chloro-3,5-dimethylphenoxy)phenylsulfonamido)-2-(benzyloxy)-*N*-((3-nitro-4-(((tetrahydro-2*H*-pyran-4-yl)methyl)amino)phenyl)sulfonyl)benzamide (21d)

4-(*N*-Benzyl-4-(4-chloro-3,5-dimethylphenoxy)phenylsulfonamido)-2-(benzyloxy)benzoic acid^11^ was coupled to 3-nitro-4-(((tetrahydro-2*H*-pyran-4-yl)methyl)amino)benzenesulfonamide (20) according to general procedure H on a scale of 0.32 mmol to give the product as a bright yellow solid (132 mg, 43%): ^1^H NMR (CDCl_3_, 400 MHz) *δ* 10.28 (1H, s, SO_2_N*H*), 8.72 (1H, d, *J* = 2.4 Hz, Ar), 8.49 (1H, t, *J* = 5.6 Hz, Ar), 8.00 (1H, d, *J* = 8.8 Hz, Ar), 7.84 (1H, d, *J* = 8.8 Hz, Ar), 7.52–7.42 (5H, m, Ar), 7.39 (2H, d, *J* = 6.8 Hz, Ar), 7.21–7.12 (5H, m, Ar), 6.98 (1H, s, N*H*CH_2_), 6.94 (2H, d, *J* = 8.4 Hz, Ar), 6.85 (1H, d, *J* = 8.8 Hz, Ar), 6.78 (2H, s, Ar), 6.58 (1H, d, *J* = 8.4 Hz, Ar), 5.09 (2H, s, O-CH_2_), 4.70 (2H, s, N-CH_2_), 4.01–3.98 (2H, m, CH_2_), 3.41–3.21 (4H, m, 2*CH_2_), 2.35 (6H, s, 2*CH_3_), 1.93–1.91 (1H, m, CH), 1.70 (2H, d, *J* = 12.4 Hz, CH_2_), 1.45–1.37 (2H, m, CH_2_); ^13^C NMR (CDCl_3_, 100 MHz) *δ* 162.1, 161.7, 156.9, 152.3, 147.9, 145.1, 138.4, 135.5, 134.9, 133.9, 132.7, 131.1, 130.7, 129.7, 129.4, 129.3, 139.1, 128.6, 128.3, 127.9, 124.8, 120.3, 119.4, 117.8, 117.2, 114.7, 117.2, 114.7, 113.7, 97.8, 72.3, 67.4, 53.8, 49.1, 34.6, 30.7, 20.9.

##### 2.1.1.30 *N*-(4-Bromo-3-methoxyphenyl)-*N*-isobutyl-4-phenoxybenzenesulfonamide (23)

4-Bromo-3-methoxyaniline (22) underwent reductive amination with isobutyraldehyde according to general procedure C on a scale of 4.95 mmol to give 4-bromo-*N*-isobutyl-3-methoxyaniline as a brown oil (1 g, 78%): ^1^H NMR (CDCl_3_, 400 MHz) *δ* 6.96 (1H, d, *J* = 8.4 Hz, Ar), 5.88 (1H, s, Ar), 5.82 (1H, d, *J* = 7.6 Hz, Ar), 3.56 (3H, s, O-CH_3_), 3.49 (1H, s br, NH), 2.62 (2H, d, *J* = 6.4 Hz, N-CH_2_), 1.63–1.56 (1H, m, CH), 0.90 (6H, d, *J* = 6.0 Hz, 2*CH_3_); ^13^C NMR (CDCl_3_, 100 MHz) *δ* 165.8, 156.4, 149.2, 133.2, 105.9, 97.3, 55.9, 51.8, 28.1, 20.5. 4-Bromo-*N*-isobutyl-3-methoxyaniline was coupled to 4-phenoxybenzene sulfonyl chloride according to general procedure D on a scale of 3.8 mmol to give the product as a beige solid (1.6 g, 89%): ^1^H NMR (CDCl_3_, 400 MHz) *δ* 7.49 (2H, d, *J* = 6.8 Hz, Ar), 7.39 (3H, t, *J* = 8.4 Hz, Ar), 7.20 (1H, t, *J* = 7.6 Hz, Ar), 7.04 (2H, d, *J* = 7.6 Hz, Ar), 6.96 (2H, d, *J* = 8.0 Hz, Ar), 6.72 (1H, s, Ar), 6.40 (1H, d, *J* = 8.0 Hz, Ar), 3.81 (3H, s, O-CH_3_), 3.26 (2H, d, *J* = 6.8 Hz, N-CH_2_), 1.61–1.55 (1H, m, CH), 0.89 (6H, d, *J* = 6.4 Hz, 2*CH_3_); ^13^C NMR (CDCl_3_, 100 MHz) *δ* 161.6, 156.1 154.9, 139.9, 133.0, 131.3, 130.2, 129.8, 125.0, 120.6, 120.3, 117.2, 113.5, 111.0, 57.8, 56.3, 26.8, 19.9.

##### 2.1.1.31 *N*-(4-Cyano-3-methoxyphenyl)-*N*-isobutyl-4-phenoxybenzenesulfonamide (24)

A stirring solution of *N*-(4-bromo-3-methoxyphenyl)-*N*-isobutyl-4-phenoxybenzenesulfonamide (23; 0.2 mmol, 100 mg, 1 eq.) in anhydrous DMF (0.1 M) was degassed, and then Zn(CN)_2_ (0.2 mmol, 24 mg, 1 eq.), Pd(PPh_3_)_4_ (0.1 mmol, 120 mg, 0.5 eq.), Zn (0.1 mmol, 10 mg, 0.5 eq.) and Zn(OAc)_2_ (0.1 mmol, 20 mg, 0.5 eq.) were added. The reaction was heated to 120 °C for 2 h. After which reaction was allowed to cool, add water, extract with EtOAc 3 times. The organic layers were combined and washed with NaHCO_3_ (aq), dried over Na_2_SO_4_, concentrated, and purified by flash column chromatography over silica gel using an eluent of Hex/EtOAc 4 : 1 to give the product as a light yellow solid (30 mg, 34%): ^1^H NMR (CDCl_3_, 400 MHz) *δ* 7.69–7.59 (4H, m, Ar), 7.46–7.41 (2H, m, Ar), 7.25 (2H, d, *J* = 7.6 Hz, Ar), 7.17 (2H, d, *J* = 8.0 Hz, Ar), 7.11 (1H, s, Ar), 6.77 (1H, d, *J* = 8.0 Hz, Ar), 4.09 (3H, s, O-CH_3_), 3.51 (2H, d, *J* = 6.8 Hz, CH_2_), 1.81–1.77 (1H, m, CH), 1.10 (6H, d, *J* = 6.4 Hz, 2*CH_3_); ^13^C NMR (CDCl_3_, 100 MHz) *δ* 161.9, 161.5, 154.8, 145.4, 133.7, 130.9, 130.2, 129.7, 125.1, 120.3, 118.8, 117.2, 115.8, 112.7, 100.8, 57.2, 56.2, 26.9, 19.9.

##### 2.1.1.32 5-(*N*-Benzyl-4-(4-chloro-3,5-dimethylphenoxy)phenylsulfonamido)-2-hydroxybenzoic acid (27)

Methyl 5-amino-2-hydroxybenzoate was reductive aminated by benzaldehyde according to general procedure C on a scale of 6.0 mmol to give methyl 5-(benzylamino)-2-hydroxybenzoate as a brown oil (1.26 g, 81%): ^1^H NMR (CDCl_3_, 400 MHz) *δ* 10.18 (1H, s, OH), 7.35–7.24 (5H, m, Ar), 7.08 (1H, s, Ar), 6.83 (2H, s, Ar), 4.26 (2H, s, N-CH_2_), 3.90 (3H, s, O-CH_3_); ^13^C NMR (CDCl_3_, 100 MHz) *δ* 170.5, 154.4, 140.6, 139.1, 128.7, 127.7, 127.4, 122.8, 118.2, 112.1, 111.7, 52.2, 49.2. Methyl 2-hydroxy-5-(isobutylamino)benzoate was coupled to 4-fluorobenzenesulfonyl chloride according to general procedure D on a scale of 4.9 mmol to yield methyl 5-(*N*-benzyl-4-fluorophenylsulfonamido)-2-hydroxybenzoate as a beige solid (1.85 g, 90%): ^1^H NMR (CDCl_3_, 400 MHz) *δ* 10.76 (1H, s, OH), 7.69–7.65 (2H, m, Ar), 7.45 (1H, d, *J* = 3.2 Hz, Ar), 7.25–7.16 (7H, m, Ar), 6.94 (1H, d, *J* = 9.2 Hz, Ar), 6.79 (1H, d, *J* = 9.2 Hz, Ar), 4.67 (2H, s, N-CH_2_), 3.00 (3H, s, O-CH_3_); ^13^C NMR (CDCl_3_, 100 MHz) *δ* 169.7, 166.5, 163.9, 161.0, 135.9, 135.3, 130.8, 130.4, 130.3, 129.6, 128.6, 128.5, 127.9, 118.2, 116.4, 116.1, 112.5, 54.8, 52.5. Methyl 5-(*N*-benzyl-4-fluorophenylsulfonamido)-2-hydroxybenzoate was *O*-benzylated according to general procedure B on a scale of 4.45 mmol to give methyl 5-(*N*-benzyl-4-fluorophenylsulfonamido)-2-(benzyloxy)benzoate as a white solid (1.21 g, 54%): ^1^H NMR (DMSO-*d*_6_, 400 MHz) *δ* 7.71–7.67 (2H, m, Ar), 7.46–7.39 (4H, m, Ar), 7.35 (2H, t, *J* = 7.2 Hz, Ar), 7.29–7.14 (8H, m, Ar), 7.07 (1H, d, *J* = 8.8 Hz, Ar), 5.09 (2H, s, O-CH_2_), 4.73 (2H, s, N-CH_2_), 3.71 (3H, s, O-CH_3_); ^13^C NMR (DMSO-*d*_6_, 100 MHz) *δ* 166.4, 165.5, 163.8, 156.9, 136.9, 136.4, 134.2, 133.8, 131.6, 131.0, 130.9, 128.9, 128.8, 128.6, 128.2, 127.9, 127.4, 120.6, 117.2, 116.9, 114.6, 70.2, 53.9, 52.5. Methyl 5-(*N*-benzyl-4-fluorophenylsulfonamido)-2-hydroxybenzoate was *O*-benzylated according to general procedure B on a scale of 4.45 mmol to give methyl 5-(*N*-benzyl-4-fluorophenylsulfonamido)-2-(benzyloxy)benzoate as a white solid (1.21 g, 54%): ^1^H NMR (DMSO-*d*_6_, 400 MHz) *δ* 7.71–7.67 (2H, m, Ar), 7.46–7.39 (4H, m, Ar), 7.35 (2H, t, *J* = 7.2 Hz, Ar), 7.29–7.14 (8H, m, Ar), 7.07 (1H, d, *J* = 8.8 Hz, Ar), 5.09 (2H, s, O-CH_2_), 4.73 (2H, s, N-CH_2_), 3.71 (3H, s, O-CH_3_); ^13^C NMR (DMSO-*d*_6_, 100 MHz) *δ* 166.4, 165.5, 163.8, 156.9, 136.9, 136.4, 134.2, 133.8, 131.6, 131.0, 130.9, 128.9, 128.8, 128.6, 128.2, 127.9, 127.4, 120.6, 117.2, 116.9, 114.6, 70.2, 53.9, 52.5. Methyl 5-(*N*-benzyl-4-fluorophenylsulfonamido)-2-(benzyloxy)benzoate was coupled to 3,5-dimethyl-4-chloro-phenol according to general procedure E on a scale of 2.4 mmol to yield the product as an ivory solid (778 mg, 50%): ^1^H NMR (CDCl_3_, 400 MHz) *δ* 7.58 (2H, d, *J* = 8.8 Hz, Ar), 7.44 (2H, d, *J* = 7.6 Hz, Ar), 7.39–7.35 (3H, m, Ar), 7.31 (1H, d, *J* = 7.2 Hz, Ar), 7.22–7.17 (5H, m, Ar), 7.16–7.13 (1H, m, Ar), 7.00 (2H, d, *J* = 9.6 Hz, Ar), 6.87–6.84 (3H, m, Ar), 5.10 (2H, s, O-CH_2_), 4.68 (2H, s, N-CH_2_), 3.84 (3H, s, O-CH_3_), 2.39 (6H, s, 2*CH_3_); ^13^C NMR (CDCl_3_, 100 MHz) *δ* 165.7, 161.6, 157.6, 152.7, 138.3, 136.2, 135.5, 135.2, 131.8, 131.5, 131.3, 130.7, 129.9, 128.6, 128.5, 127.9, 127.7, 126.7, 120.6, 120.1, 117.4, 113.9, 70.6, 51.7, 52.1, 20.9. Methyl 5-(*N*-benzyl-4-(4-chloro-3,5-dimethylphenoxy)phenylsulfonamido)-2-(benzyloxy)benzoate was saponified according to general procedure F except the temperature was raised to 60 °C on a scale of 1.21 mmol to afford 5-(*N*-benzyl-4-(4-chloro-3,5-dimethylphenoxy)phenylsulfonamido)-2-(benzyloxy)benzoic acid as a beige solid (645 mg, 85%): ^1^H NMR (CDCl_3_, 400 MHz) *δ* 7.58 (1H, d, *J* = 2.4 Hz, Ar), 7.52 (2H, d, *J* = 8.8 Hz, Ar), 7.41–7.38 (5H, m, Ar), 7.21–7.16 (5H, m, Ar), 6.99–6.96 (3H, m, Ar), 6.84 (2H, s, Ar), 5.18 (2H, s, O-CH_2_), 4.68 (2H, s, N-CH_2_), 2.36 (6H, s, 2*CH_3_); ^13^C NMR (CDCl_3_, 100 MHz) *δ* 164.6, 161.9, 156.6, 152.5, 138.3, 137.8, 135.2, 133.8, 133.2, 131.6, 130.8, 129.8, 129.3, 129.2, 128.6, 128.3, 128.0, 127.8, 127.6, 120.3, 117.4, 114.5, 113.7, 72.5, 54.4, 20.9. 5-(*N*-Benzyl-4-(4-chloro-3,5-dimethylphenoxy)phenylsulfonamido)-2-(benzyloxy)benzoic acid was debenzylated according to general procedure G on a scale of 0.16 mmol to give 5-(*N*-benzyl-4-(4-chloro-3,5-dimethylphenoxy)phenylsulfonamido)-2-hydroxybenzoic acid (27) as a beige solid (69 mg, 80%): ^1^H NMR (DMSO-*d*_6_, 400 MHz) *δ* 7.59 (2H, d, *J* = 8.4 Hz, Ar), 7.29 (1H, s, Ar), 7.23–7.19 (5H, m, Ar), 7.16–7.06 (3H, m, Ar), 7.00 (2H, s, Ar), 6.75 (1H, d, *J* = 8.8 Hz, Ar), 4.68 (2H, s, N-CH_2_), 2.31 (6H, s, 2*CH_3_); ^13^C NMR (DMSO-*d*_6_, 100 MHz) *δ* 161.4, 153.1, 138.4, 136.5, 135.8, 131.9, 130.9, 130.4, 130.1, 129.7, 128.8, 128.6, 127.9, 120.7, 118.0, 117.9, 54.2, 20.8; HRMS-ESI: *m*/*z* found 536.0945 [M–H]^−^, C_28_H_23_ClNO_6_S requires 536.0940.

##### 2.1.1.33 5-(*N*-Benzyl-4-(4-chloro-3,5-dimethylphenoxy)phenylsulfonamido)-2-hydroxy-*N*-((3-nitro-4-(((tetrahydro-2*H*-pyran-4-yl)methyl)amino)phenyl)sulfonyl)benzamide (29)

5-(*N*-Benzyl-4-(4-chloro-3,5-dimethylphenoxy)phenylsulfonamido)-2-(benzyloxy)benzoic acid (27) was coupled to 3-nitro-4-(((tetrahydro-2*H*-pyran-4-yl)methyl)amino)benzenesulfonamide (20) according to general procedure H on a scale of 0.24 mmol to give 5-(*N*-benzyl-4-(4-chloro-3,5-dimethylphenoxy)phenylsulfonamido)-2-(benzyloxy)-*N*-((3-nitro-4-(((tetrahydro-2*H*-pyran-4-yl)methyl)amino)phenyl)sulfonyl)benzamide (29) as a bright yellow solid (53 mg, 25%): ^1^H NMR (CDCl_3_, 400 MHz) *δ* 10.32 (1H, s, N*H*SO_2_), 8.70 (1H, s, Ar), 8.49 (1H, t, *J* = 5.2 Hz, Ar), 7.98 (1H, d, *J* = 9.6 Hz, Ar), 7.61 (1H, d, *J* = 2.4 Hz, Ar), 7.52–7.40 (7H, m, Ar), 7.31 (1H, d, *J* = 8.4 Hz, Ar), 7.24–7.16 (5H, m, Ar), 6.95–6.92 (3H, m, Ar and N*H*CH_2_), 6.86–6.83 (3H, m, Ar), 5.49 (2H, s, O-CH_2_), 4.62 (2H, s, N-CH_2_), 4.00 (2H, d, *J* = 11.6 Hz, CH_2_), 3.38 (2H, t, *J* = 10.8 Hz, CH_2_), 3.22 (2H, t, *J* = 6.4 Hz, CH_2_), 2.38 (6H, s, 2*CH_3_), 2.00–1.92 (1H, m, CH), 1.69 (2H, d, *J* = 12.4 Hz, CH_2_), 1.42–1.36 (2H, m, CH_2_); ^13^C NMR (CDCl_3_, 100 MHz) *δ* 161.8, 161.4, 156.2, 152.5, 147.9, 138.4, 137.1, 135.3, 135.2, 133.9, 133.0, 131.5, 131.0, 130.7, 129.7, 129.5, 129.3, 128.9, 128.5, 128.4, 127.9, 127.8, 124.7, 120.3, 119.4, 117.4, 113.7, 113.6, 72.5, 67.4, 54.3, 49.1, 34.6, 30.7, 20.9. 5-(*N*-Benzyl-4-(4-chloro-3,5-dimethylphenoxy)phenylsulfonamido)-2-(benzyloxy)-*N*-((3-nitro-4-(((tetrahydro-2*H*-pyran-4-yl)methyl)amino)phenyl)sulfonyl)benzamide was debenzylated according to general procedure G on a scale of 0.06 mmol to give the product as a bright yellow solid (22 mg, 45%): ^1^H NMR (DMSO-*d*_6_, 400 MHz) *δ* 8.51 (2H, m, Ph-N*H* and Ar), 7.85 (1H, d, *J* = 8.8 Hz, Ar), 7.59 (2H, d, *J* = 7.6 Hz, Ar), 7.47 (1H, d, *J* = 8.0 Hz, Ar), 7.19–7.07 (8H, m, Ar), 6.99 (2H, s, Ar), 6.56–6.51 (2H, m, Ar), 4.73 (2H, s, N-CH_2_), 3.79 (2H, d, *J* = 9.6 Hz, CH_2_), 3.28–3.18 (4H, m, 2*CH_2_), 2.97 (6H, s, 2*CH_3_), 1.87–1.84 (1H, m, CH), 1.55 (2H, d, *J* = 12.4 Hz, CH_2_), 1.21–1.18 (2H, m, CH_2_); ^13^C NMR (DMSO-*d*_6_, 100 MHz) *δ* 161.4, 153.0, 147.3, 143.4, 138.4, 136.5, 134.8, 131.8, m 130.3, 129.8, 128.8, 128.3, 127.8, 120.7, 118.1, 117.8, 116.5, 115.1, 67.0, 53.2, 48.3, 34.2, 30.6, 20.7; HRMS-ESI: *m*/*z* found 833.1886 [M–H]^−^, C_40_H_38_ClN_4_O_10_S_2_ requires 833.1723.

### 2.2 Biology

#### 2.2.1 Protein production

A His6-MBP tagged recombinant human Mcl-1 residues 172 to 327 was produced in *E. coli* in either LB or minimal media supplemented with ^15^NH_4_Cl to produce unlabeled or ^15^N-labeled Mcl-1. The tagged protein was initially purified from the crude cell lysate by IMAC chromatography (GE Healthcare Life Sciences), and after dialysis to remove the imidazole the affinity tag was cleaved using PreScission Protease (GE Healthcare Life Sciences). A Sephacryl S-200 size exclusion column was used as a final purification step before the protein was concentrated with a 10 000 MWCO centrifugal filter concentrator (Millipore). The protein purity was shown to be >98% by Coomassie Brilliant Blue (Bio-Rad) stained SDS-PAGE gel and the final concentration was determined using the Bradford protein assay (Bio-Rad) with BSA standards (Pierce).

BCl-xL was purchased from SinBiological.

#### 2.2.2 Peptide synthesis and purification

A 6-aminohexanoic acid linker was conjugated to the N-terminus of the Bak BH3 peptide (GQVGRQLAIIGDDINR), capped with fluorescein (on the amino group of the linker), and the peptide was amidated on the C-terminus to give FITC-Ahx-GQVGRQLAIIGDDINR-CONH_2_, hereafter referred to as “FITC-Bak” (synthesized by Neo BioScience in >95% purity).

#### 2.2.3 Fluorescence polarization experiments

Fluorescence polarization experiments were conducted using a BMG PHERAstar FS multimode microplate reader equipped with two PMTs for simultaneous measurements of the perpendicular and parallel fluorescence emission. The assays were performed in black polypropylene 384-well microplate (Costar) with a final volume of 20 mL. The affinity (*K*_d_) of the FITC-Bak peptide was determined by titrating Mcl-1^172-327^ into 10 nM FITC-Bak peptide in 20 mM HEPES, pH 6.8, 50 mM NaCl, 3 mM DTT, 0.01% Triton X-100 and 5% DMSO at room temperature while monitoring the perpendicular and parallel fluorescence emission with a 485 nm excitation and 520 nm emission filters. The fluorescence polarization competition assay (FPCA) was performed using 100 nM Mcl-1^172-327^ (or 15 nM Bcl-x_L_^2-212^ (R&D Systems)) in the same buffer (thus, 10 nM FITC-Bak) with varying concentrations of either unlabeled peptide or 2,6-disubstituted nicotinate. Regression analysis was carried out using Origin (OriginLab, Northampton, MA) to fit the data to the Hill equation to determine the IC_50_, which delivered *K*_d_s for the FITC-Bak peptide to Mcl-1 of 33.8 ± 0.50 nM and to Bcl-x_L_ of 6.67 ± 0.05 nM. For the fluorescence polarization competition titrations, an equation derived by Nikolovska-Coleska *et al.*^16^ was used to calculate the *K*_i_ from the IC_50_ data. All experiments were run in three biological replicates, each in triplicate.

#### 2.2.4 Cell viability assay

Cell viability studies were conducted using the CellTiter-Blue® (Promega, Madison, WI) assay. Briefly, HL60 cells, were seeded in a 96-well plate and maintained at 37 °C with 5% CO_2_ for 24 h. The cells were then exposed to test compounds at 11 concentrations ranging from 0 to 20 mM. After 72 h, cell viabilities were then determined according to the manufacturer's instructions.

## 3 Results and discussion

Fig. 1 depicts the target molecules of this work, which vary in the salicylic acid portion in blue (and in some cases the R^1^ group is iBu instead of Bn). Although the lead compound was “17cd” (*K*_i_s = 0.629 (MCL-1) and 1.67 (BCL-x_L_) μM), for this work, we initially developed analogues of the simpler compound 1.^11^ Methyl ester 2 and acetoxymethyl ester 3 are proposed as prodrugs of 1,^11^ similarly to how we improved the cell permeability of our lead Myc inhibitor JY-3-094.^17^ Classical bioisosteric targets include the hydroxamic acid 4, which has a p*K*_a_ of around 8–9,^18^ as well as tetrazole 5, 3-hydroxyisoxazole 6 (both of 5 and 6 lack the phenyl hydroxyl group owing to synthetic challenges), and acylsulfonamides 7, all of which have p*K*_a_ values of around 4–5,^18^*i.e.* akin to that of the parent carboxylic acid. Cyanophenol 8, bearing a p*K*_a_ of about 7, is an unconventional/putative bioisostere. It is noteworthy that the acylsulfonamide functionality may be further developed beyond the group itself (R^2^ group in 7), without compromising the acidity, to gain additional interactions with the target. Indeed, Fesik's group previously expanded their MCL-1 inhibitors in this fashion,^19^ and it is of interest to elucidate if a similar modification of our inhibitors is tolerated for targeting not only MCL-1 but BCL-xL as well. Likewise, the NH of hydroxamic acids, such as in 4,^20^ and the CH alkene carbon of 6, may be further elaborated whilst retaining the acidic NH.

**Scheme 1:**
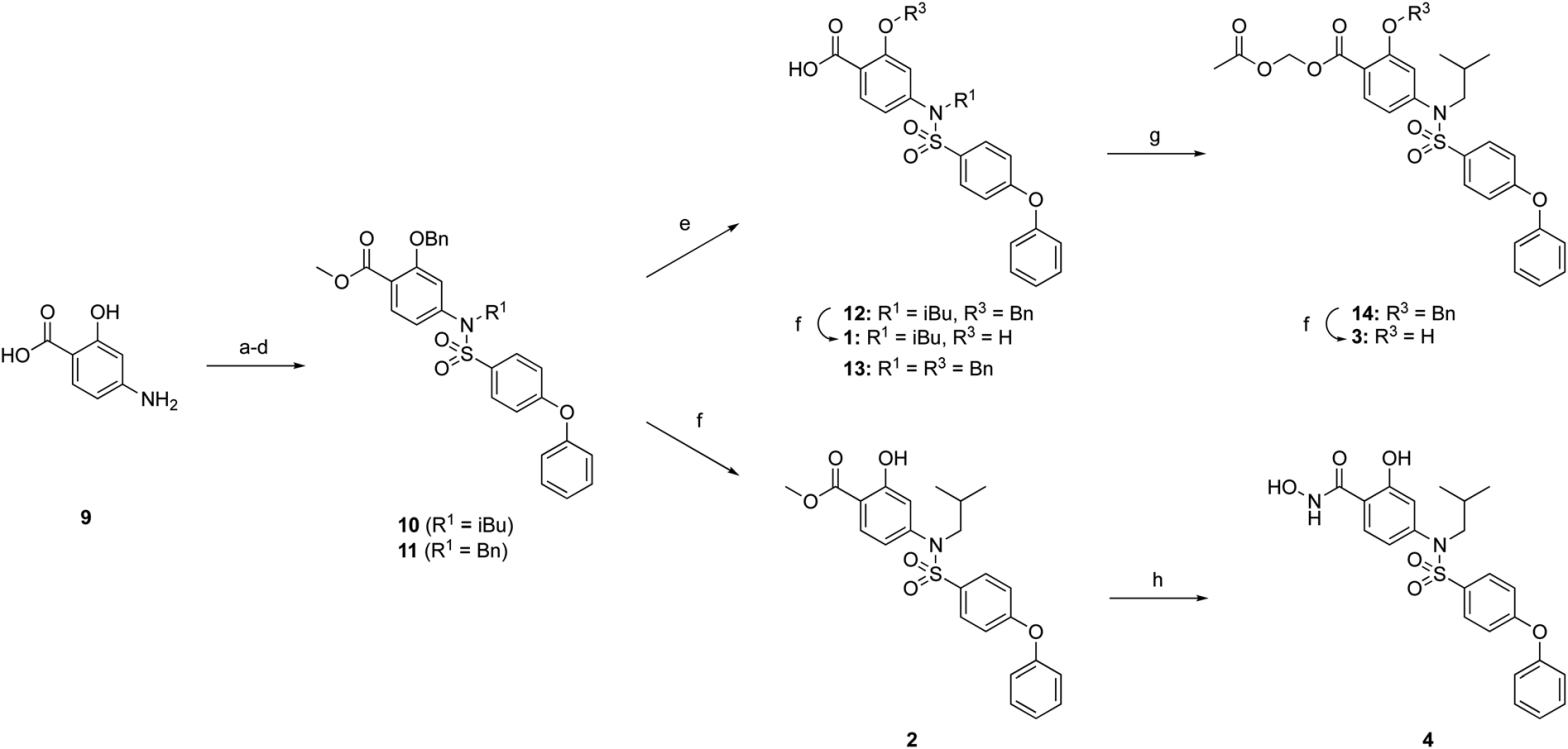
Reagents and conditions: (a) H_2_SO_4_, MeOH, reflux, overnight, 95%; (b) BnBr, K^*t*^OBu, DMF, RT, overnight, 70%; (c) isobutyraldehyde or benzaldehyde, NaBH(OAc)_3_, DCE, acetic acid, RT, overnight, 46%; (d) 4-phenoxybenzenesulfonyl chloride, DIPEA, DMAP, CHCl_3_, 60 °C, overnight, 96%; (e) LiOH·H_2_O, THF-MeOH-H_2_O, 3 : 1:1, RT, overnight, 57–99%; (f) TFA, toluene, RT, overnight, 80–89%; (g) bromomethyl acetate, K_2_CO_3_, DMF, RT, overnight, 68%; (h) NH_2_OH·HCl, NaOH, H_2_O, dioxane, RT, overnight, 71%.

The ester prodrugs 2 and 3 were prepared from 4-aminosalicyclic acid (9), as shown in Scheme 1. As previously described,^11^ esterification, *O*-benzylation, followed by reductive amination with either isobutyraldehyde or benzaldehyde, and lastly sulfonylation of the furnished secondary amines delivered tertiary sulfonamides 10 (ref. 11) and 11, respectively. Saponification of esters of 10 and 11 yielded acids 12 and 13, and then TFA-mediated debenzylation^21^ of 12 furnished salicylic acid lead compound 1. Re-esterification of 12 with acetoxymethyl bromide followed again by an uneventful debenzylation afforded the acetoxymethyl ester prodrug 3. Meanwhile, debenzylation of 10 yielded methyl ester prodrug 2 again in excellent yield. Finally, hydroxamic acid 4 was acquired by treatment of 2 with hydroxylamine under basic conditions.

**Scheme 2:**
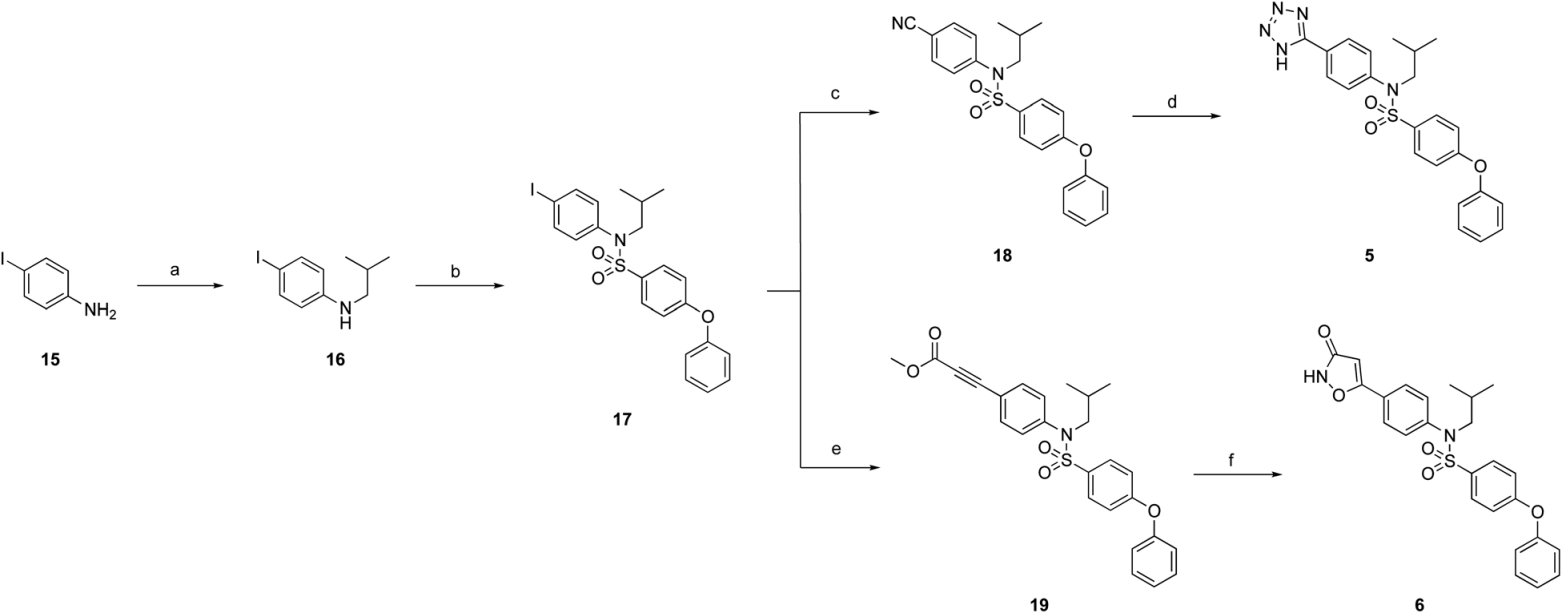
Reagents and conditions: (a) isobutyraldehyde, NaBH(OAc)_3_, DCE, acetic acid, RT, overnight, 78%; (b) 4-phenoxybenzenesulfonyl chloride, DIPEA, DMAP, CHCl_3_, 60 °C, overnight, 89%; (c) Zn(CN)_2_, Pd(PPh_3_)_4_, Zn-Zn(OAc)_2_, 1 : 1, DMF, 120 °C, 2 h, 41%; (d) NaN_3_, NH_4_Cl, DMF, 125 °C, overnight, 76%; (e) PPh_3_, CuI, Et_3_N, Pd(PPh_3_)_2_Cl_2_, methyl propionate, 110 °C, μwaves, 40 min, 44%; (f) NH_2_OH·HCl, NaOH, MeOH, RT, overnight, 20%.

Towards the synthesis of tetrazole 5 (Scheme 2), 4-iodoaniline (15) underwent reductive amination with isobutyraldehyde, and then *N*-sulfonylation yielded tertiary sulfonamide 17. Subsequently, Pd-catalyzed cyanation of 17 with zinc cyanide, doped with zinc dust and zinc acetate,^22^ delivered nitrile 18. Finally, a cycloaddition of azide to nitrile 18 delivered the desired tetrazole 5. Alternatively, iodide 17 was subjected to a Sonogashira coupling with methyl propionate to furnish the alkyne derivative 19 (moderate yield largely due to incomplete reaction), which was then transformed into 3-hydroxyisoxazole 6 upon treatment with hydroxylamine.

**Scheme 3:**
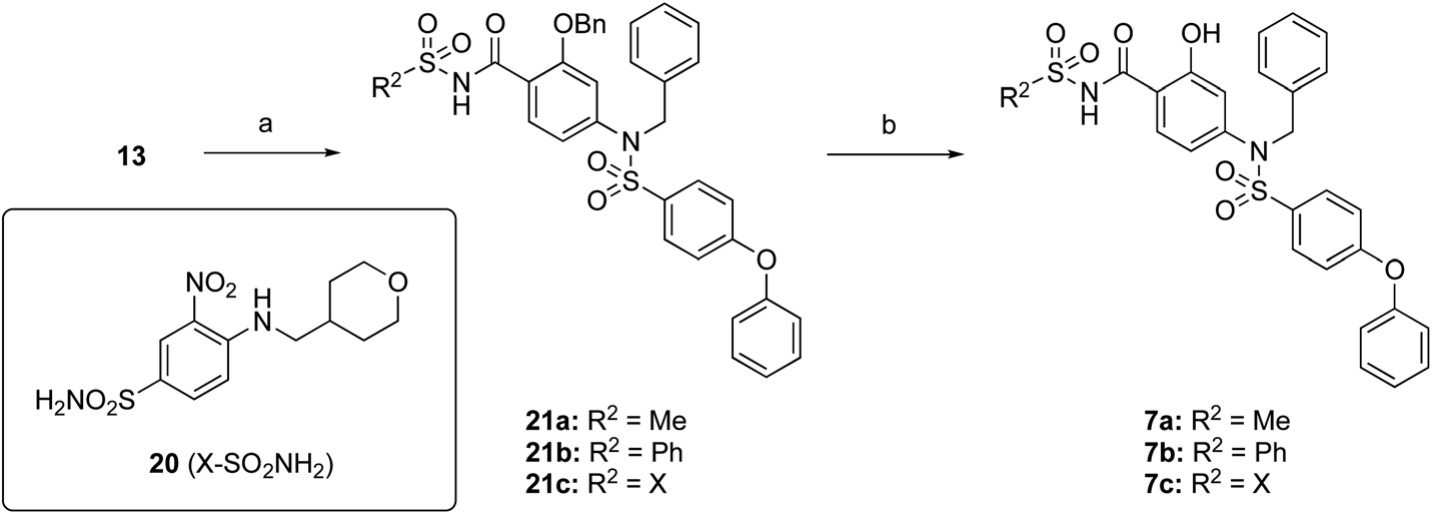
Reagents and conditions. (a) R^2^SO_2_NH_2_, EDCI·HCl, DMAP, CH_2_Cl_2_, RT, overnight, 30–56%; (b) TFA, toluene, RT, overnight, 40–50%.

The target acylsulfonamides 7a–c were prepared by coupling either methanesulfonamide, benzenesulfonamide or 20, respectively, to carboxylic acid 13, followed by TFA-driven debenzylation, as depicted in Scheme 3.

**Scheme 4:**
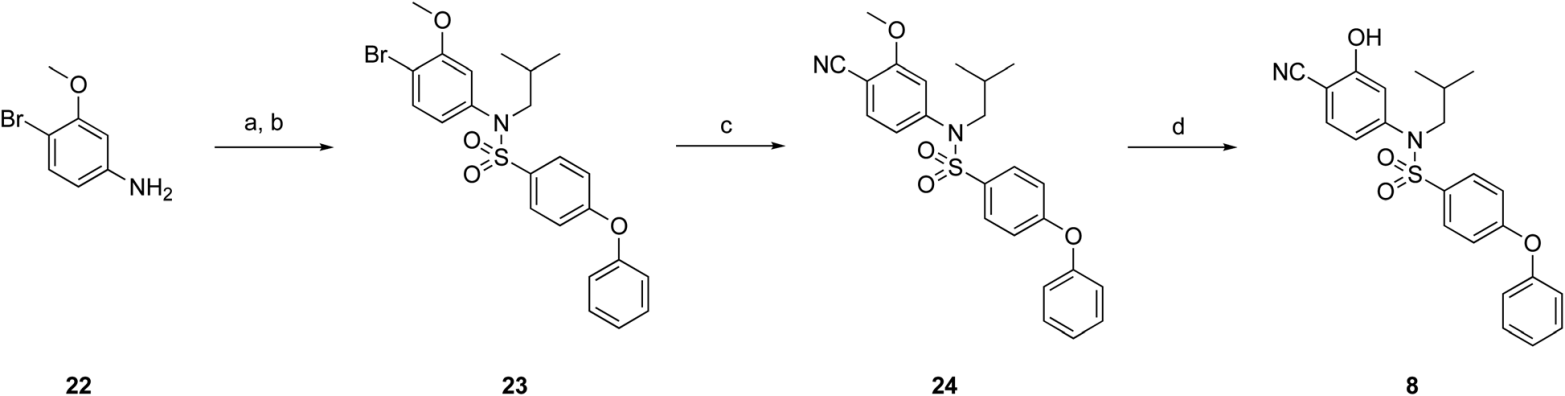
Reagents and conditions: (a) isobutyraldehyde, NaBH(OAc)_3_, DCE, acetic acid, RT, overnight, 78%; (b) 4-phenoxybenzenesulfonyl chloride, DIPEA, DMAP, CHCl_3_, 60 °C, overnight, 89%; (c) Zn(CN)_2_, Pd(PPh_3_)_4_, Zn-Zn(OAc)_2_, 1 : 1, DMF, 120 °C, 2 h, 34%; (d) LiCl, DMF, 160 °C, μwaves, 30 min, 40%.

As illustrated in Scheme 4, the cyanophenol variant of salicylic acid-based 1, was accessed from 4-bromo-3-methoxyaniline (22). Briefly, aniline 22 underwent reductive amination with isobutyraldehyde followed by sulfonylation with 4-phenoxybenzenesulfonyl chloride to afford tertiary sulfonamide 23. Pd-catalyzed cyanation of 23 as before delivered nitrile 24. Demethylation with LiCl under microwave irradiation then yielded the target cyanophenol 8.

As shown in Table 1, we initially evaluated these compounds against MCL-1 using a standard fluorescence polarization competition assay (FPCA), as described by us^11^ and others previously.^14,19,23^ For reference, the previously reported data for compound 1 is included.^11^ Predictably, esters 2 and 3 were inactive, confirming our previous findings that the carboxylic acid, which likely binds Arg263, is crucial to activity.^11,12^ Disappointingly, 3-hydroxyisoxazole 6 and salicylonitrile 8 demonstrated no inhibition of MCL-1. While hydroxamic acid 4 resulted in a significant reduction in affinity relative to control compound 1, which may be attributed to the reduced p*K*_a_ of the hydroxamic acid functional group, the tetrazole (5) and acylsulfonamide (7a and 7b) bioisosteres, on the other hand with comparable acidities to carboxylic acids, were around ≥2-fold better than the parent compound 1. We acknowledge there are subtle differences between 5, 7a and 7b and the parent 1, but our previous work indicates that the salicylic hydroxyl of 1 contributes positively to binding, and inhibitors with R^1^ = iBu or Bn that are otherwise identical result in comparable binding affinities (see Tables 1 and 2 in ref. 11). Thus, the introduction of tetrazole and acylsulfonamides as carboxylic acid surrogates into this class of dual MCL-1/BCL-xL inhibitors is tolerated for MCL-1 inhibition.

**Table 1:** MCL-1 binding affinities of ester prodrugs and bioisosteres of 1

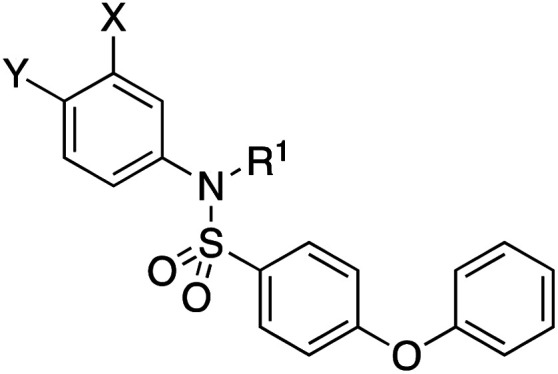									
Number	X	Y	R^1^	*K*_i_ (μM)	Number	X	Y	R^1^	*K*_i_ (μM)
1	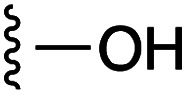	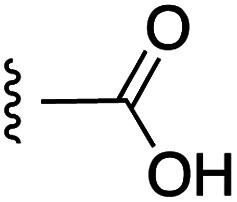	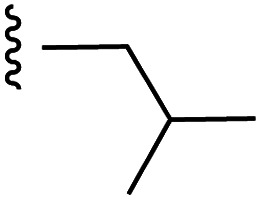	5.77 ± 0.46	6	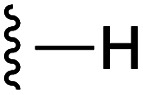	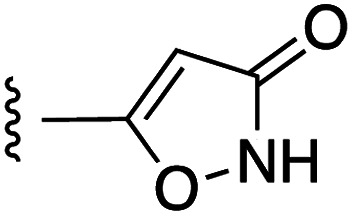	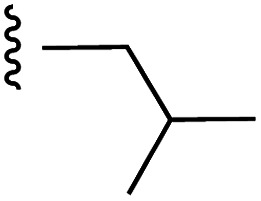	>500
2	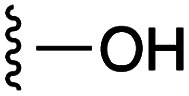	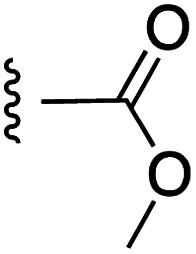	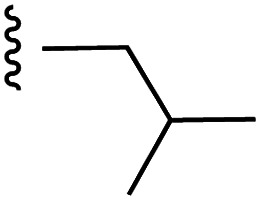	>500	7a	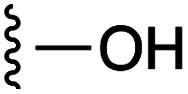	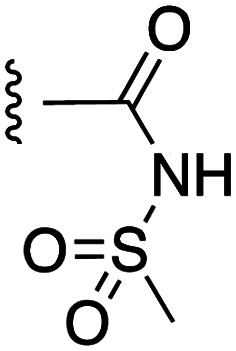	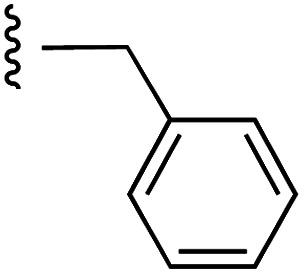	2.94 ± 0.35
3	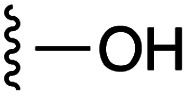	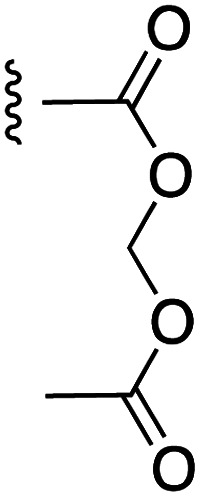	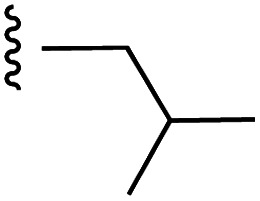	>500	7b	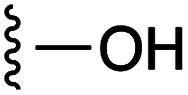	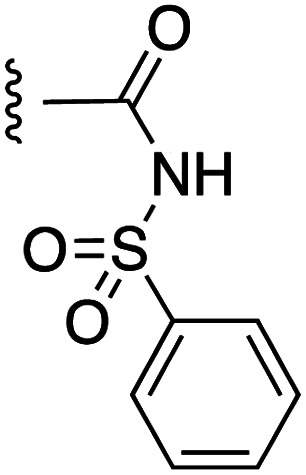	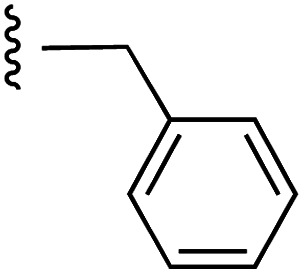	1.95 ± 0.22
4	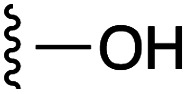	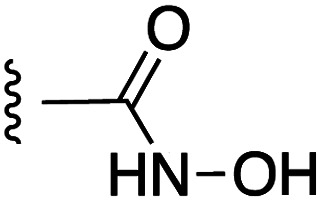	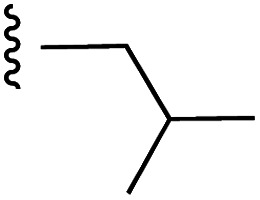	70.6 ± 5.8	8	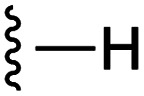	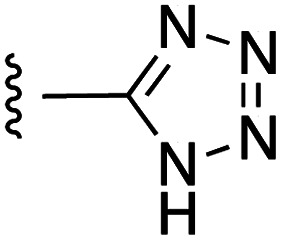	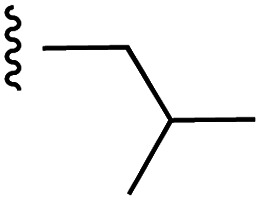	>500
5	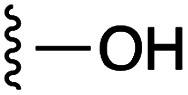	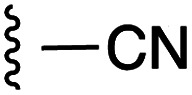	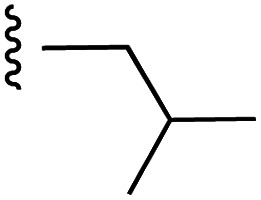	3.29 ± 0.26					

The inhibition data for the acylsulfonamides were particularly encouraging, and so we decided to investigate them further, both by expanding the series as well as by evaluating their activities against BCL-xL. As illustrated in Table 2, acylsulfonamide 7a inhibited BCL-xL about 4-fold weaker than parent compound 17cd, although this is likely due to the different R^4^ groups, as also observed in Table 2 in ref. 11. Nevertheless, switching the methyl sulfonamide of 7a to the phenyl sulfonamide in 7b elicited a 3-fold improvement in BCL-x_L_ inhibitory activity, such that 7b is on a par with “17cd”.^11^ In addition, this trend was also observed in the MCL-1 inhibition data. Elaboration of the sulfonamide portion with that present in the BCL-2 inhibitor venetoclax yielded acyl sulfonamide 7c, which afforded even greater improvements in inhibition of both proteins, although at the same time, these improvements were minimal, indicating limited interactions of these sulfonamide moieties with the protein surfaces. In order to create a better comparison, we synthesized 7d, the congener of 7c in which the R^4^ group was the same as in 17cd. We were delighted to observe further increases in activity, with 7d equipotent to 26 at inhibiting both MCL-1 and BCL-x_L_. Switching the hydroxyl and acylsulfonamide functionalities as in 29 was unremarkable with similar binding affinities to isomer 7d (and also to parent salicylic acids 27 and “6e-OH-5”^11^). In summary, whilst the acylsulfonamide is clearly tolerated as a surrogate for the carboxylic acid in this class of dual MCL-1/BCL-x_L_ inhibitors, it is surprising that the increase in size of the sulfonamide group from 7a to 7b to 7c yielded minimal improvements in inhibition of MCL-1 and BCL-x_L_.

**Table 2:** MCL-1 and BCL-x_L_ binding affinities of acylsulfonamide bioisosteres of 17cd from ref. 11

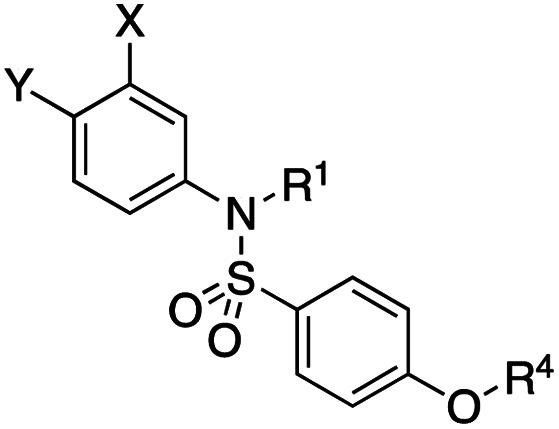							
Number	X	Y	R^1^	R^4^	*K*_i_ (MCL-1, μM)	*K*_i_ (BCL-x_L_, μM)	Selectivity (*K*_i_ MCL-1/*K*_i_ BCL-x_L_)
“17cd”^11^	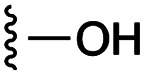	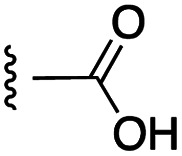	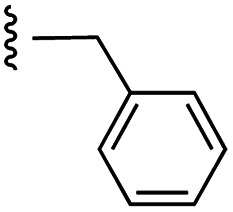	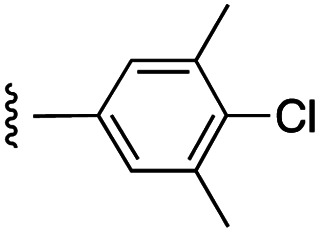	0.629 ± 0.05	1.63 ± 0.18	1 : 2.65
7a	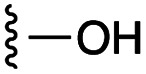	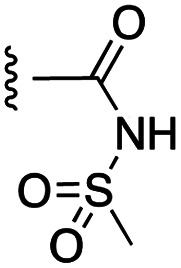	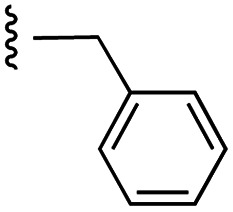	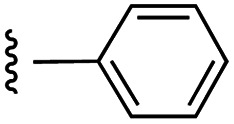	2.94 ± 0.35	7.22 ± 1.07	1 : 2.45
7b	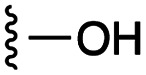	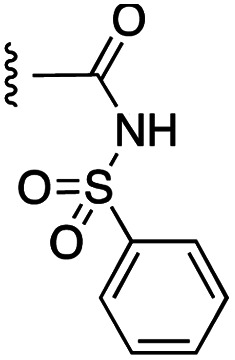	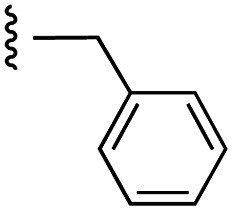	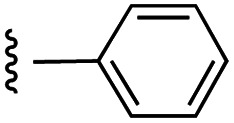	1.95 ± 0.22	2.34 ± 0.22	1 : 1.20
7c	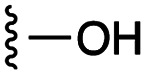	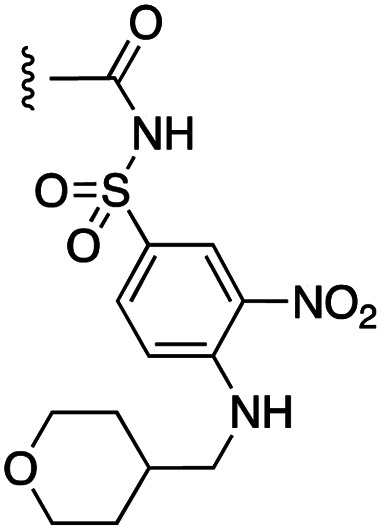	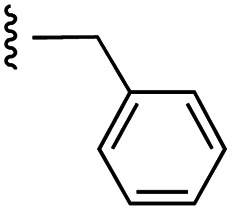	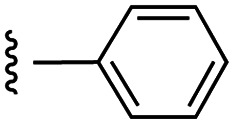	1.23 ± 0.15	1.05 ± 0.14	1 : 0.85
7d	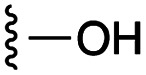	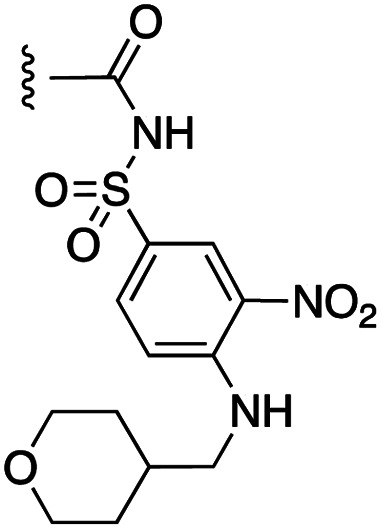	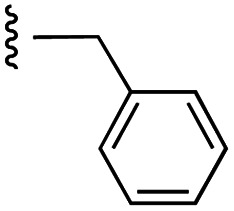	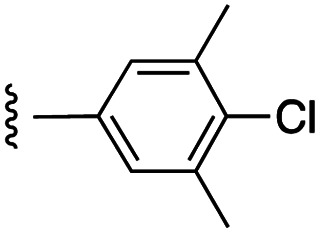	0.800 ± 0.07	1.82 ± 0.18	1 : 2.02
27	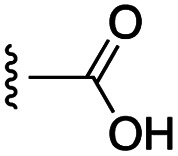	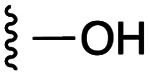	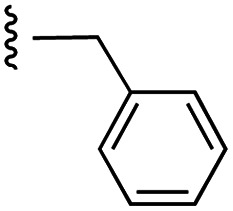	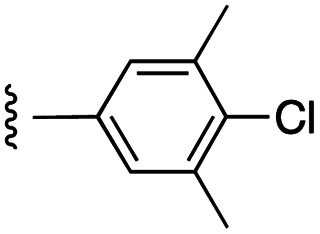	1.40 ± 0.47	1.82 ± 0.23	1 : 1.30
“6e-OH-5”^11^	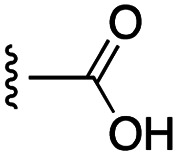	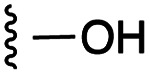	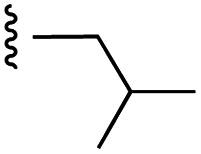	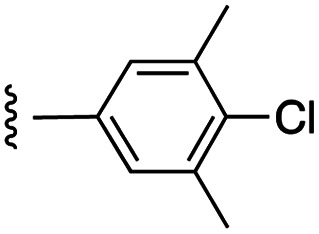	0.778 ± 0.041	2.19 ± 0.19	1 : 1.30
29	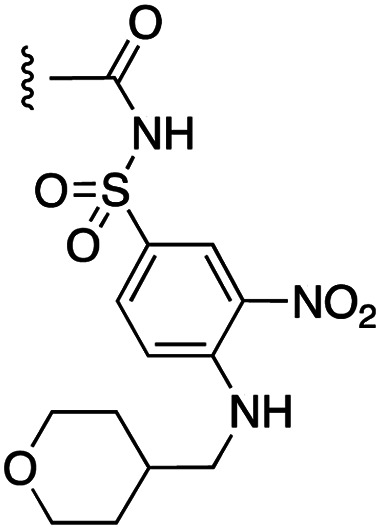	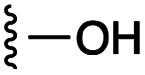	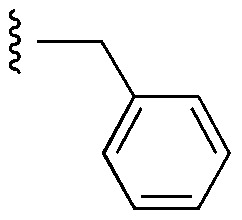	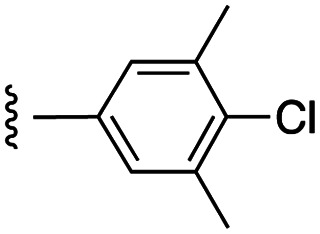	1.01 ± 0.08	1.54 ± 0.23	1 : 4.96

Lastly, we evaluated the cell killing activities of a small batch of our dual inhibitors with the human leukemia HL60 cell line that is known to be sensitive to MCL-1 and BCL-x_L_ inhibitors,^24^ and the data is shown in Table 3. Carboxylic acids 1, 26 and 28 were just a few-fold worse than their cell-free *K*_i_ values with 0.5% FBS. Ester prodrugs 2 and 3 were not as effective at killing cells as the parent acid from which they were derived, which may suggest incomplete ester hydrolysis under the conditions of the experiment – we intend to investigate this in future work. Pleasingly, tetrazole 5 and acyl sulfonamide 7d were somewhat comparable, or better, than the parent acid 1. In all but once instance, the cell killing activities were significantly reduced in the presence of a greater concentration of FBS, which was anticipated since lipophilic molecules with acidic groups, such as ours, bind albumin.^15^ The similar activities for neutral methyl ester 2 in 0.5% FBS and 10% FBS suggests 2 likely does not bind albumin. Although the cell activity of ester congener 3 was impacted by a greater concentration of FBS, this may be explained by greater ester hydrolysis outside of the cell since the acetoxymethyl ester is far more prone to hydrolysis than is the methyl ester.^17^

**Table 3:** Cell killing of select compounds in HeLa cells, in the presence of varying amounts of FBS

Compound	HL60 IC_50_ (μM)	
	0.15% FBS	10% FBS
1	18.5	>120
2	68.1	76.4
3	46.4	>120
5	15.7	>120
7d	7.45	>120
26	5.79	53.2
28	9.82	>120

## 4 Conclusions

In conclusion, we prepared ester prodrugs and carboxylic acid bioisosteres of one of our lead dual MCL-1/BCL-x_L_ inhibitors. As predicted, the ester prodrugs 2 and 3 were inactive in the cell-free MCL-1 binding assay, which we presume was due to the lack of a carboxylic acid, and hence the loss of a crucial salt bridge most probably with Arg263, but unfortunately they were also less active than the parent carboxylic acid 1 in HL60 cells, likely due to incomplete ester hydrolysis. The introduction of a tetrazole as a carboxylic acid surrogate was tolerated, yielding the compound 5 that inhibited MCL-1 with a similar activity to the parent carboxylic acid. We also prepared a small library of acylsulfonamide bioisosteres (7a–d, 29) and evaluated their inhibitory activities against both MCL-1 and BCL-x_L_. Pleasingly, this carboxylic acid surrogate was also tolerated across both proteins. At the same time, we were surprised that the series of acylsulfonamides exhibited rather similar inhibitory activities to one another, and so before we pursue the further optimization of our dual inhibitors, it is paramount that we obtain an X-ray co-crystal structure of one of our lead compounds with either MCL-1 or BCL-x_L_ to facilitate ligand design. Lastly, we evaluated some of our best compounds in HL60 cells, and discovered that the tetrazole 5 and acylsulfonamide 7d were as potent as, or slightly better than, the parent acids 1 and “17cd”.

## Conflicts of interest

There are no conflicts to declare.

## Supplementary Material

RA-013-D3RA05711A-s001
