# Supplementary material for: Tetrazole and acylsulfonamide bioisosteric replacements of the carboxylic acid in a dual MCL-1/BCL-xL inhibitor are tolerated
Source: RSC Adv. 2023 Nov 22;13(49):34322–34. doi: 10.1039/d3ra05711a (PMC10664828; doi:10.1039/d3ra05711a)
Supplement: RA-013-D3RA05711A-s001 [file RA-013-D3RA05711A-s001.pdf]

Electronic Supplementary Material (ESI) for RSC Advances.
This journal is © The Royal Society of Chemistry 2023

Compound 2

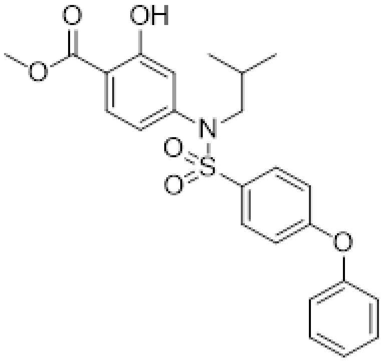

Sample Name:

Data Collected on:
400mr-vnmrs400
Archive directory:

Sample directory:

FidFile: PROTON

Pulse Sequence: PROTON (s2pul)
Solvent: cdcl3
Data collected on: Jan 27 2015

Temp. 25.0 C / 298.1 K
Operator: work

Relax. delay 2.000 sec
Pulse 79.4 degrees
Acq. time 1.278 sec
Width 6410.3 Hz
4 repetitions
OBSERVE H1, 399.7512853 MHz
DATA PROCESSING
Line broadening 0.5 Hz
FT size 16384
Total time 0 min 13 sec

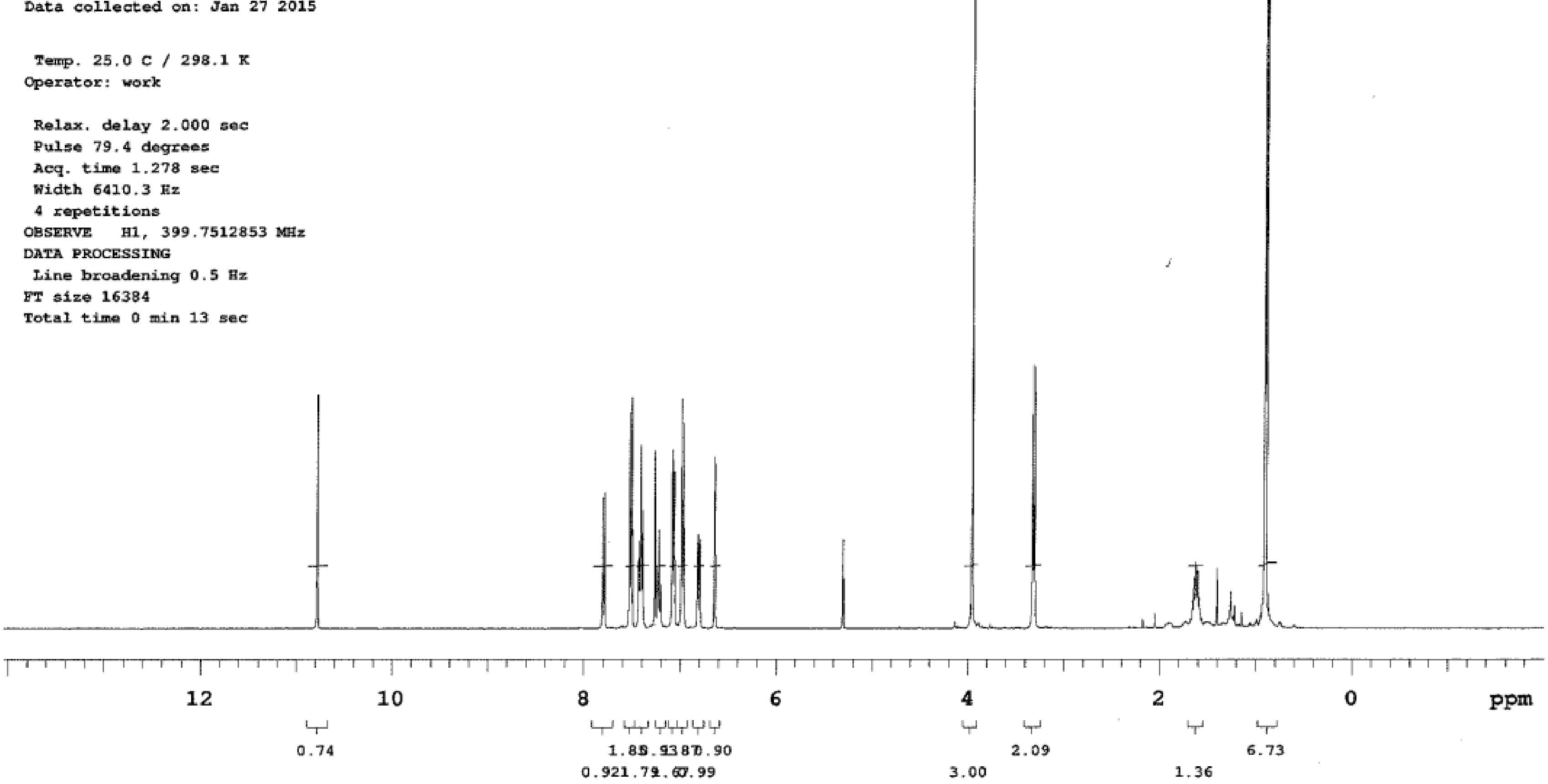

Compound 2

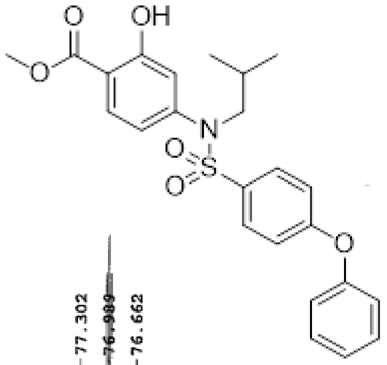

Sample Name:

Data Collected on:
400mr-vnmrs400
Archive directory:

Sample directory:

FidFile: CARBON

Pulse Sequence: CARBON (s2pul)
Solvent: cdcl3
Data collected on: Jan 27 2015

Temp. 25.0 C / 298.1 K
Operator: work

Relax. delay 1.000 sec
Pulse 58.1 degrees
Acq. time 0.813 sec
Width 24509.8 Hz
280 repetitions
OBSERVE C13, 100.5175428 MHz
DECOUPLE H1, 399.7532925 MHz
Power 39 dB
continuously on
WALTZ-16 modulated
DATA PROCESSING
Line broadening 3.0 Hz
FT size 32768
Total time 30 min

VARIAN

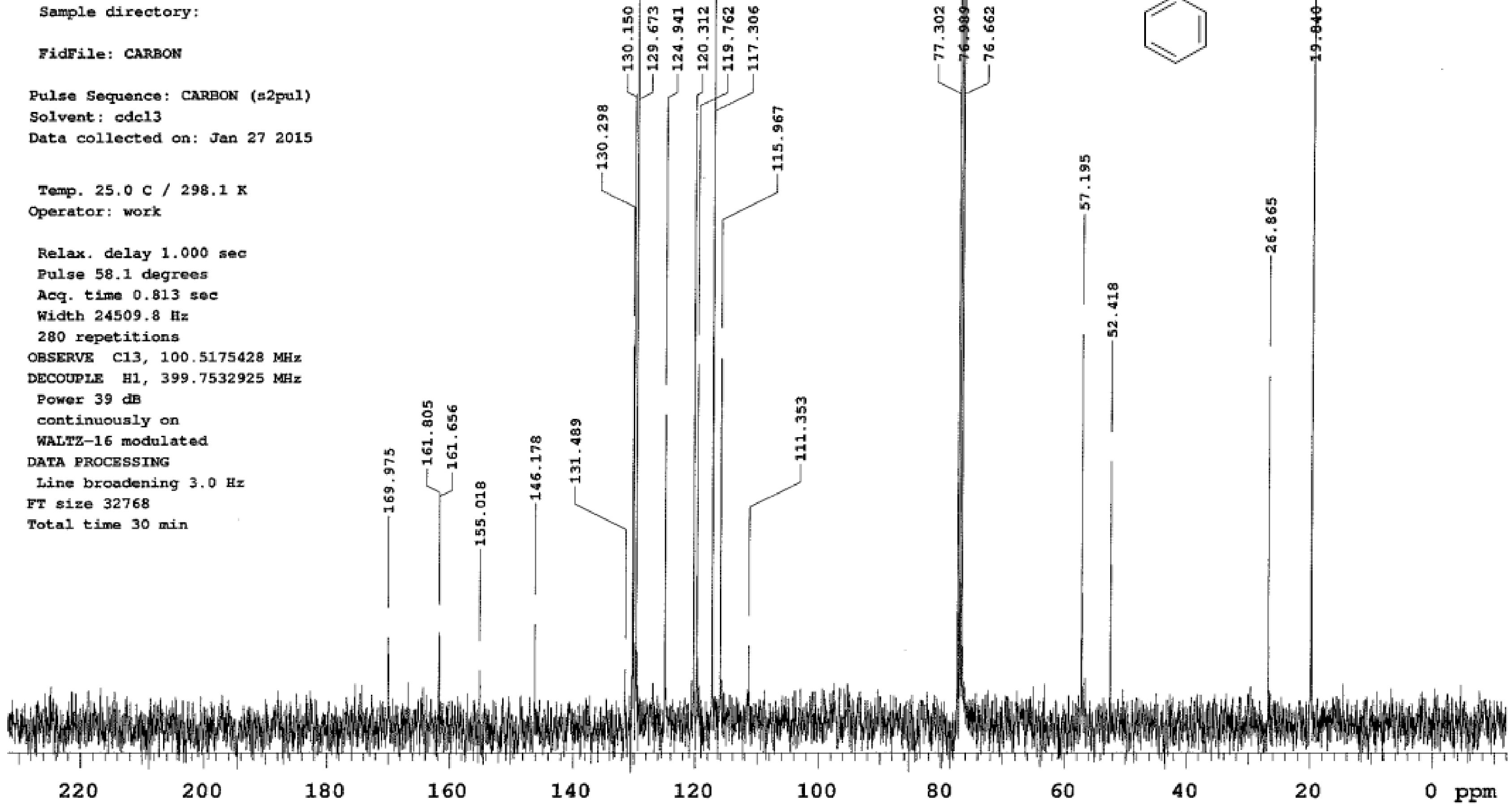

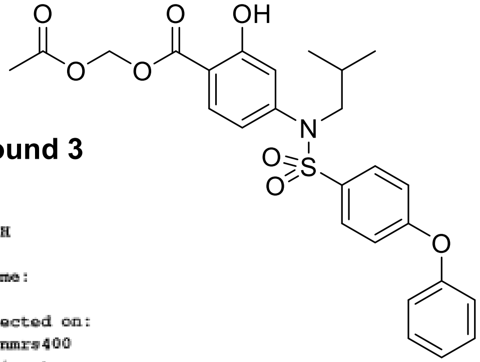

Compound 3

LC-4-141-1H

Sample Name:

Data Collected on:
400mr-vnmrs400
Archive directory:

Sample directory:

FidFile: PROTON

Pulse Sequence: PROTON (s2pul)
Solvent: dmso
Data collected on: Feb 21 2023

Temp. 25.0 C / 298.1 K
Operator: work

Relax. delay 5.000 sec
Pulse 92.6 degrees
Acq. time 1.278 sec
Width 6410.3 Hz
8 repetitions
OBSERVE H1, 399.7525412 MHz
DATA PROCESSING
Line broadening 0.5 Hz
FT size 16384
Total time 3 min 21 sec

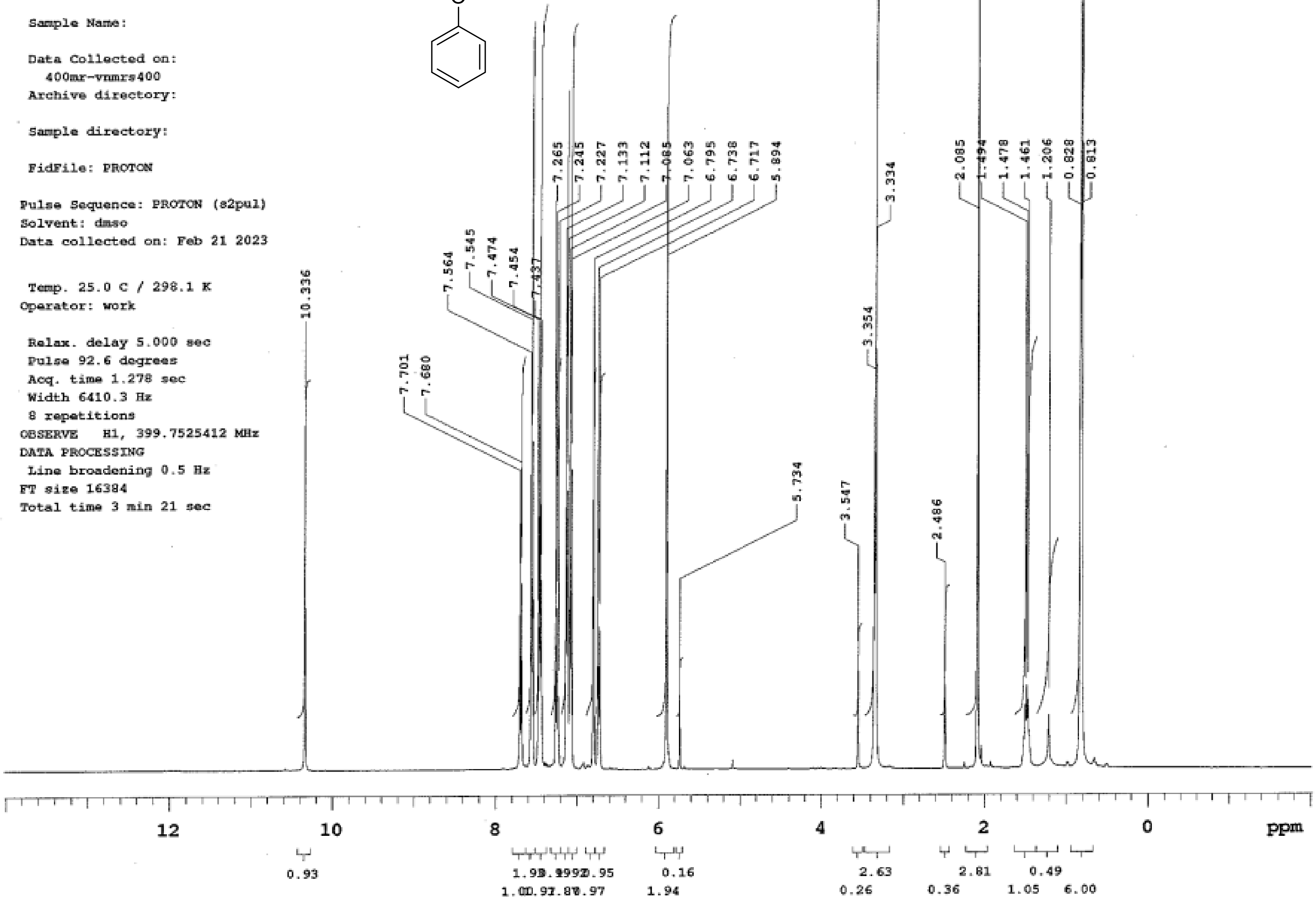

Compound 3

BDL-2-138-LC-4-141-C13

Sample Name:

Data Collected on:
400mr-vnmrs400
Archive directory:

Sample directory:

FidFile: BDL-2-138-LC-4-141-C13

Pulse Sequence: CARBON (s2pul)
Solvent: dmso
Data collected on: Feb 21 2023

Temp. 25.0 C / 298.1 K
Operator: work

Relax. delay 5.000 sec
Pulse 67.7 degrees
Acq. time 0.813 sec
Width 21186.4 Hz
204 repetitions
OBSERVE C13, 100.5178564 MHz
DECOUPLE H1, 399.7545399 MHz
Power 39 dB
continuously on
WALTZ-16 modulated
DATA PROCESSING
Line broadening 3.0 Hz
FT size 32768
Total time 12 hr, 55 min

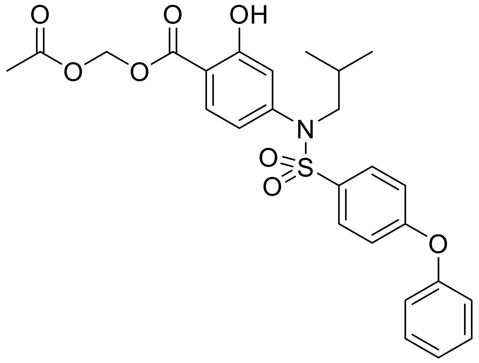

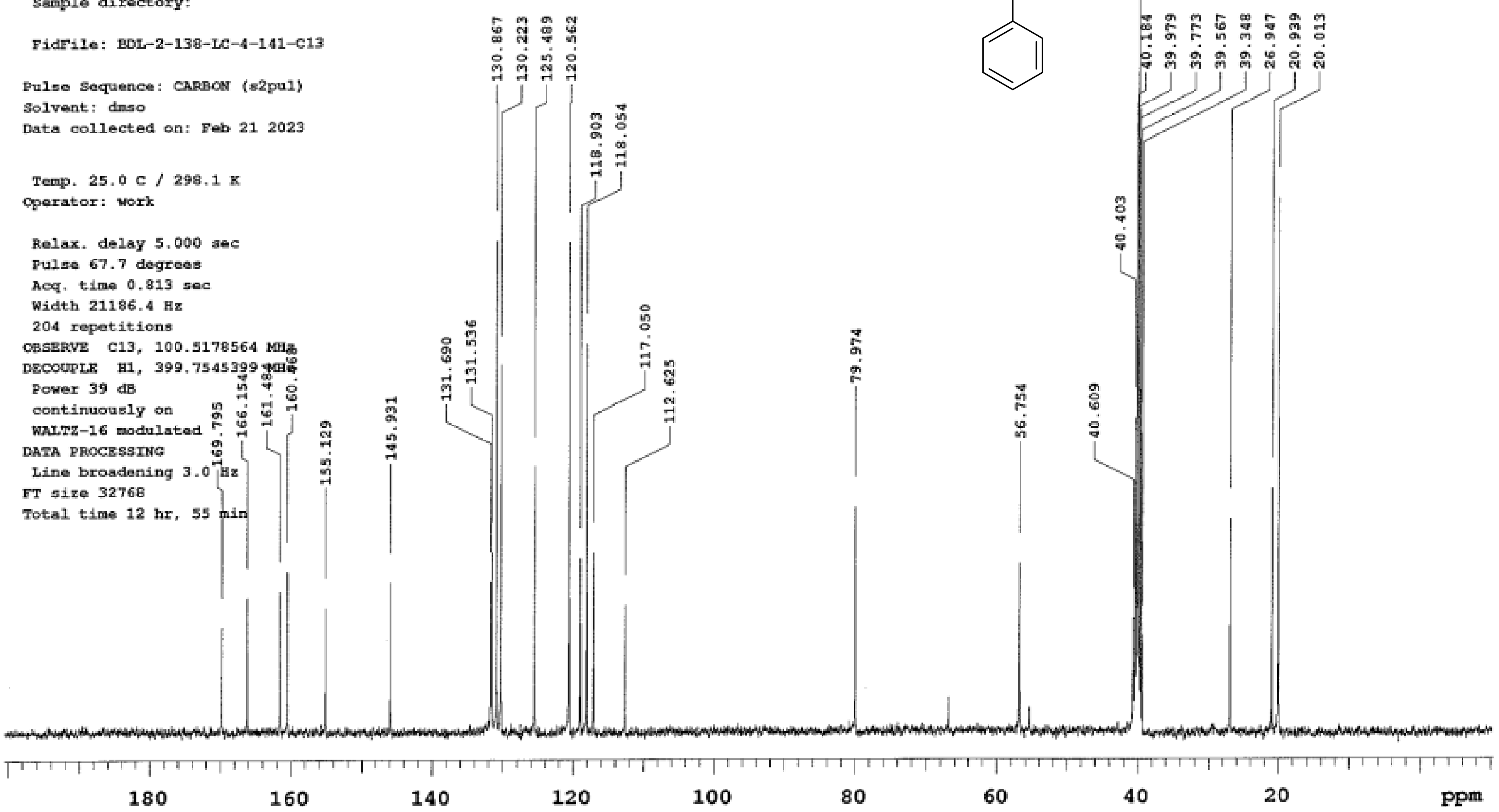

Compound 4

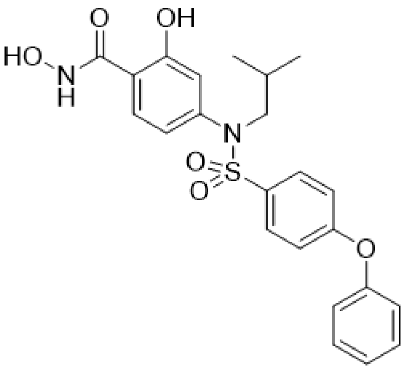

LC-5-061-1H-BDL-rerun

Sample Name:

Data Collected on:
400mr-vnmrs400
Archive directory:

Sample directory:

FidFile: PROTON

Pulse Sequence: PROTON (s2pul)
Solvent: dmso
Data collected on: Feb 28 2023

Temp. 25.0 C / 298.1 K
Operator: work

Relax. delay 5.000 sec
Pulse 92.6 degrees
Acq. time 1.278 sec
Width 6410.3 Hz
8 repetitions
OBSERVE H1, 399.7525412 MHz
DATA PROCESSING
Line broadening 0.5 Hz
FT size 16384
Total time 3 min 21 sec

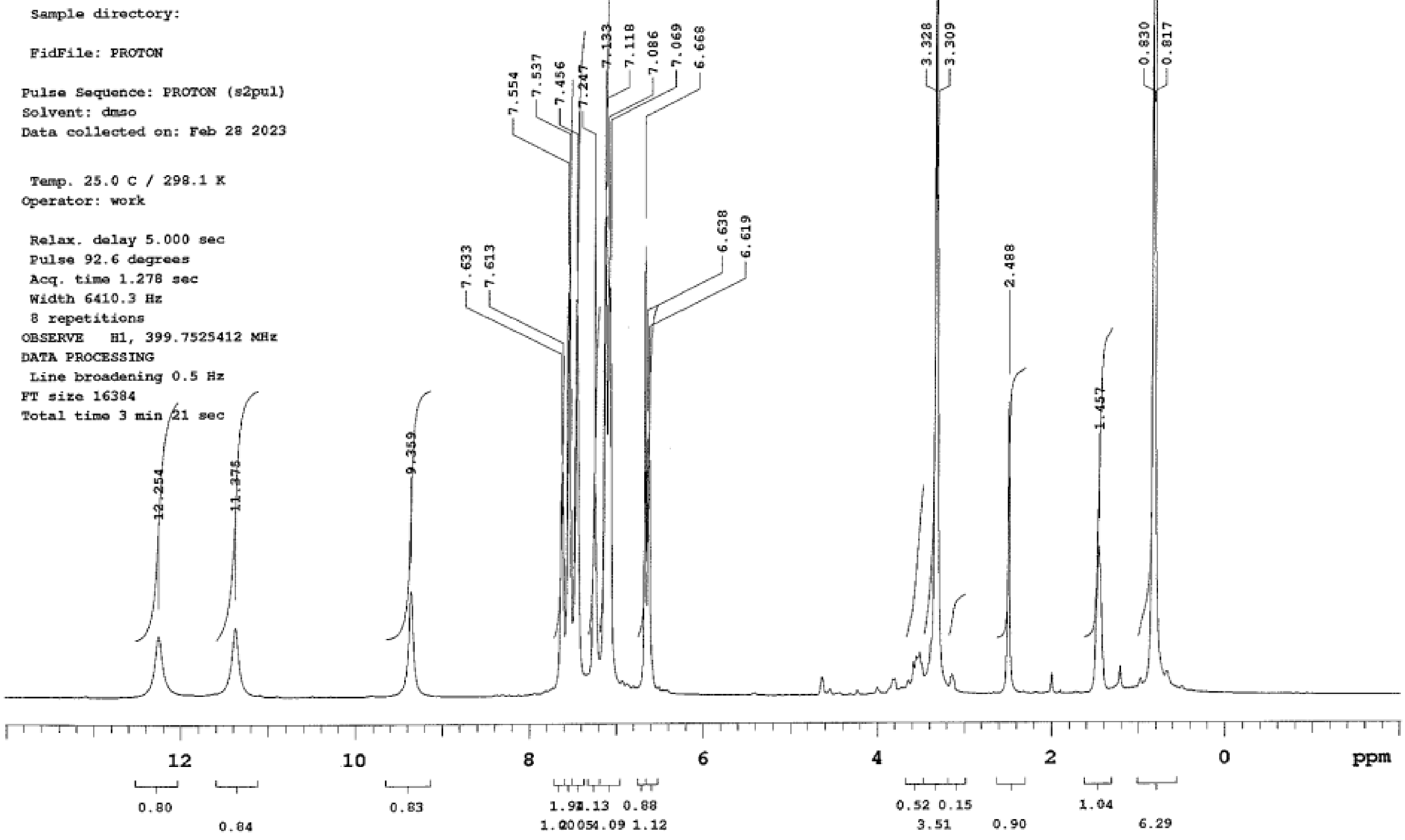

Compound 4

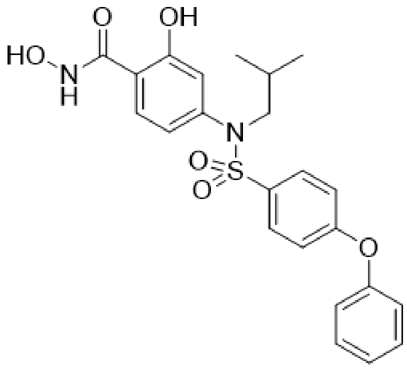

LC-5-061-C13

Sample Name:

Data Collected on:
400mr-vnmrs400
Archive directory:

Sample directory:

FidFile: CARBON

Pulse Sequence: CARBON (s2pul)
Solvent: dmso
Data collected on: Feb 21 2023

Temp. 25.0 C / 298.1 K
Operator: work

Relax. delay 5.000 sec
Pulse 67.7 degrees
Acq. time 0.813 sec
Width 21186.4 Hz
200 repetitions
OBSERVE C13, 100.5178564 MHz
DECOUPLE H1, 399.7545399 MHz
Power 39 dB
continuously on
WALTZ-16 modulated
DATA PROCESSING
Line broadening 3.0 Hz
FT size 32768
Total time 12 hr, 55 min

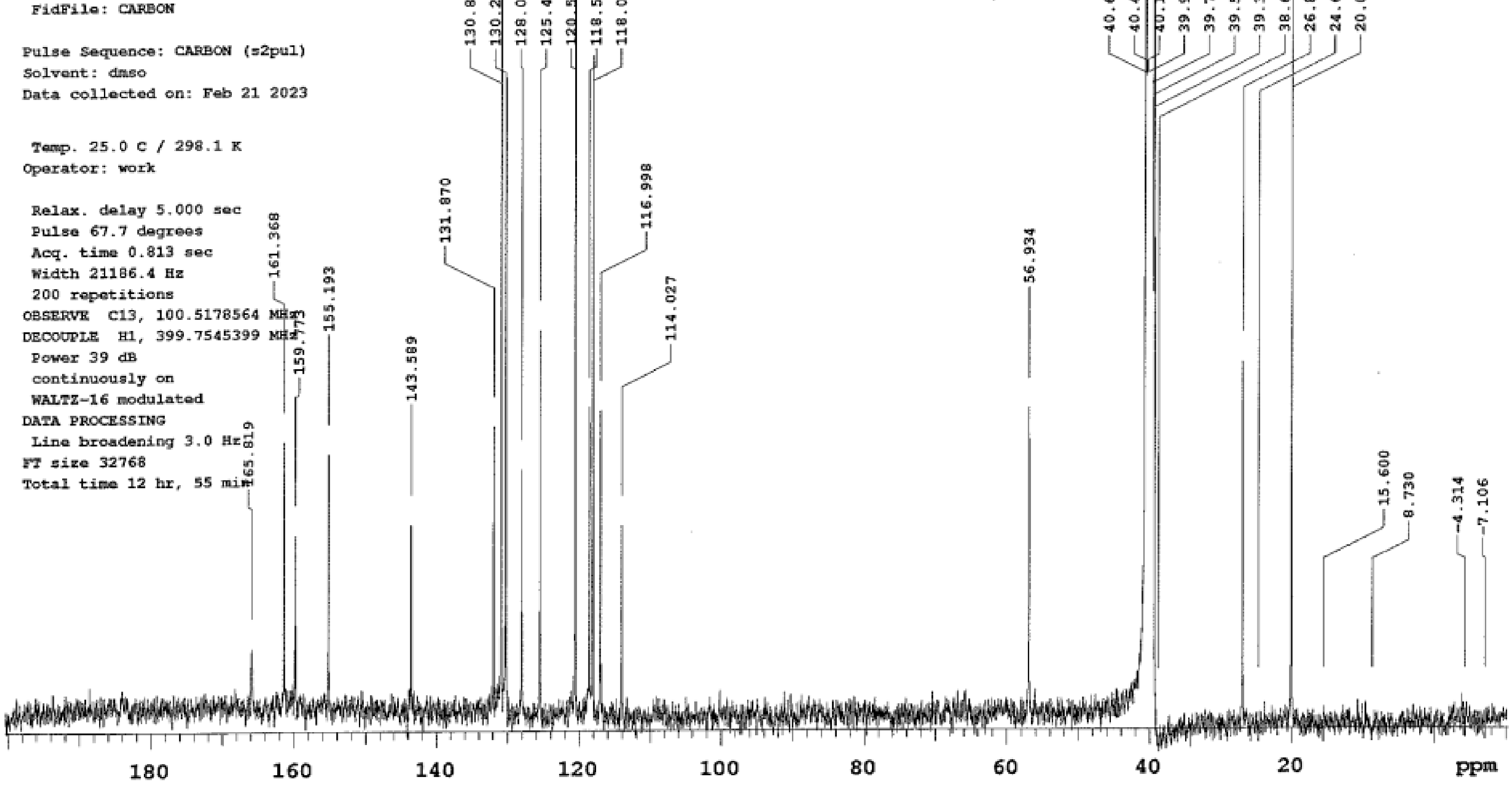

Compound 5

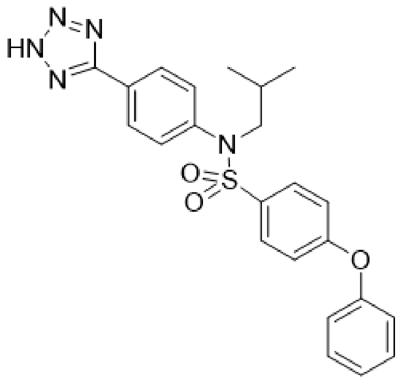

LC-5-068-1H-2

Sample Name:

Data Collected on:
400mr-vnmrs400
Archive directory:

Sample directory:

FidFile: PROTON

Pulse Sequence: PROTON (s2pul)
Solvent: dmso
Data collected on: Mar 13 2023

Temp. 25.0 C / 298.1 K
Operator: work

Relax. delay 5.000 sec
Pulse 92.6 degrees
Acq. time 1.278 sec
Width 6410.3 Hz
8 repetitions
OBSERVE H1, 399.7525412 MHz
DATA PROCESSING
Line broadening 0.5 Hz
FT size 16384
Total time 3 min 21 sec

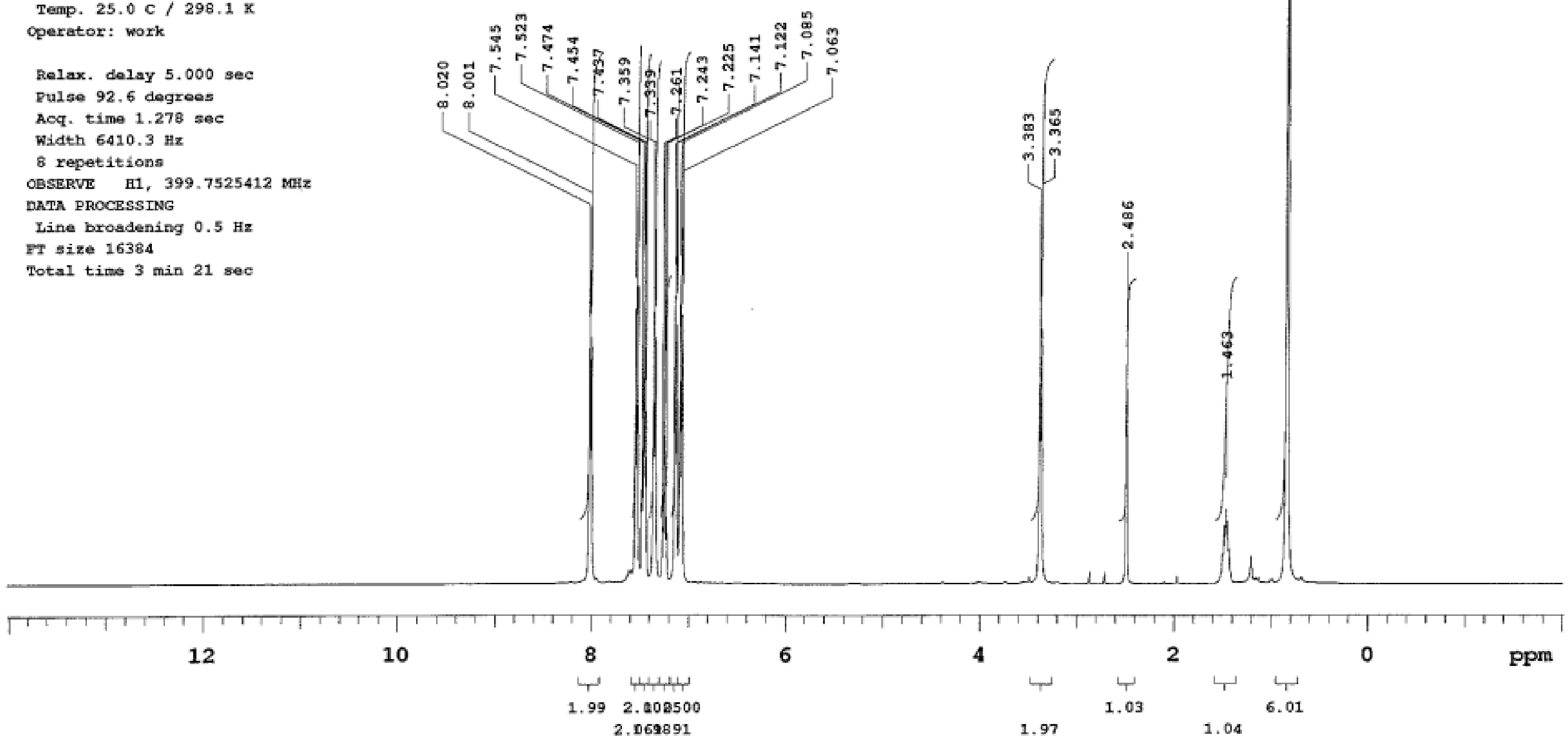

Compound 5

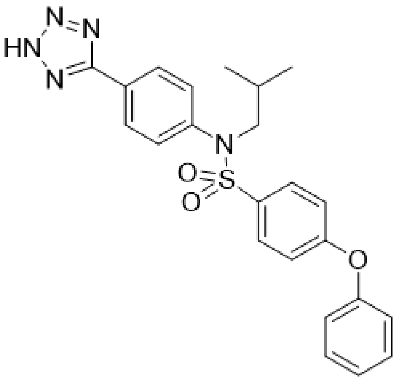

LC-5-068-C13

Sample Name:

Data Collected on:
400mr-vnmrs400
Archive directory:

Sample directory:

FidFile: CARBON

Pulse Sequence: CARBON (s2pul)
Solvent: dmso
Data collected on: Mar 13 2023

Temp. 25.0 C / 298.1 K
Operator: work

Relax. delay 5.000 sec
Pulse 67.7 degrees
Acq. time 0.813 sec
Width 21186.4 Hz
394 repetitions
OBSERVE C13, 100.5178564 MHz
DECOUPLE H1, 399.7545399 MHz
Power 39 dB
continuously on
WALTZ-16 modulated
DATA PROCESSING
Line broadening 3.0 Hz
FT size 32768
Total time 12 hr, 55 min

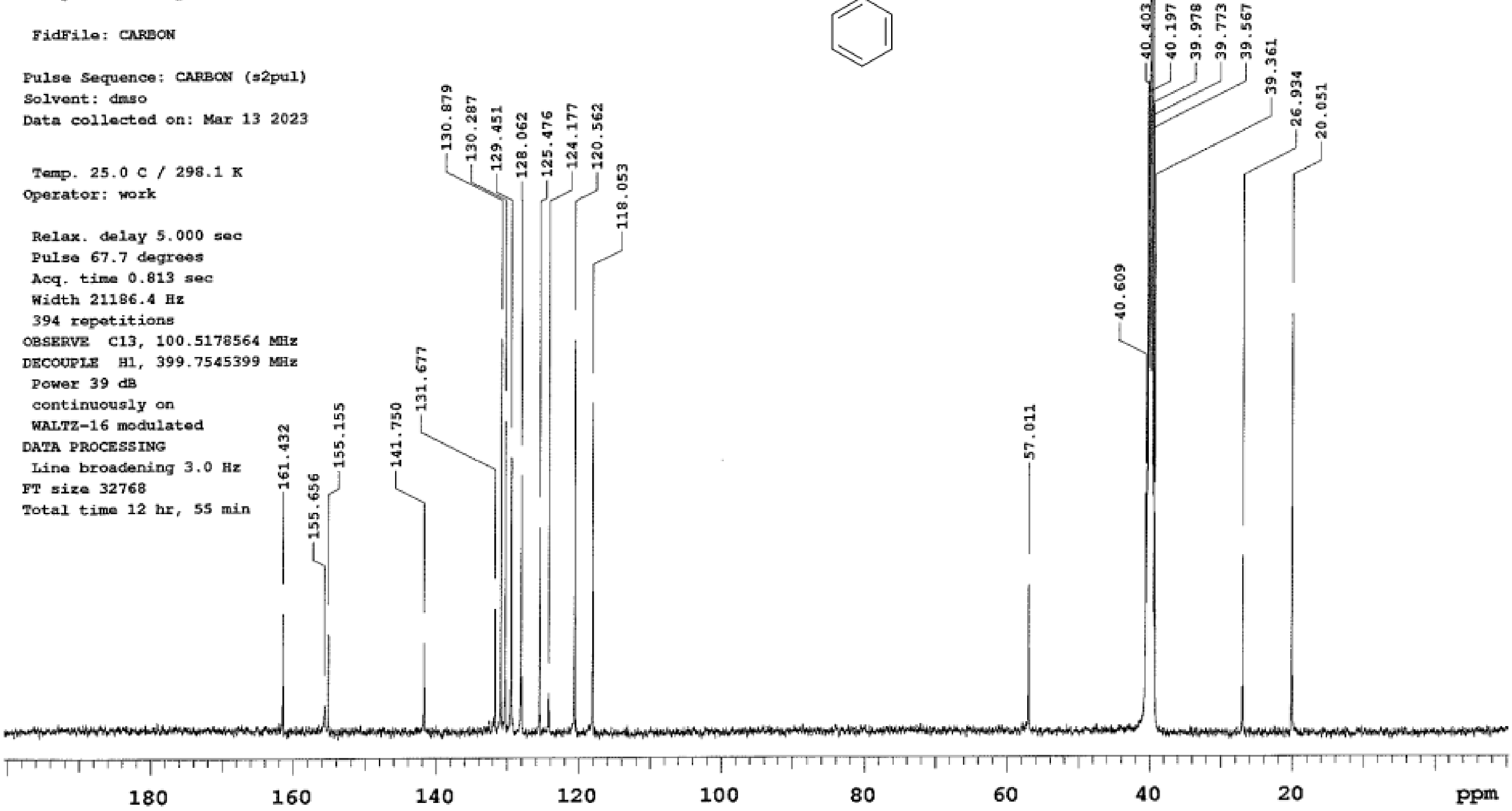

Compound 6

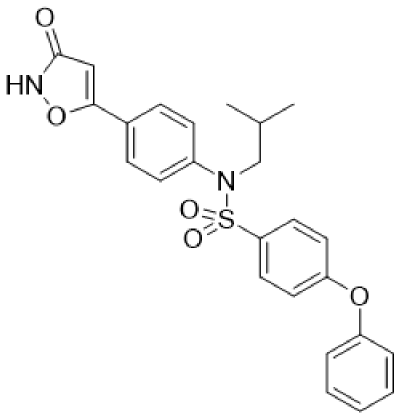

LC-5-069-1H-BDL-rerun

Sample Name:

Data Collected on:
400mr-vnmrs400
Archive directory:

Sample directory:

FidFile: PROTON

Pulse Sequence: PROTON (s2pul)
Solvent: dmso
Data collected on: Feb 28 2023

Temp. 25.0 C / 298.1 K
Operator: work

Relax. delay 5.000 sec
Pulse 79.4 degrees
Acq. time 1.278 sec
Width 6410.3 Hz
8 repetitions
OBSERVE H1, 399.7525411 MHz
DATA PROCESSING
Line broadening 0.5 Hz
FT size 16384
Total time 3 min 21 sec

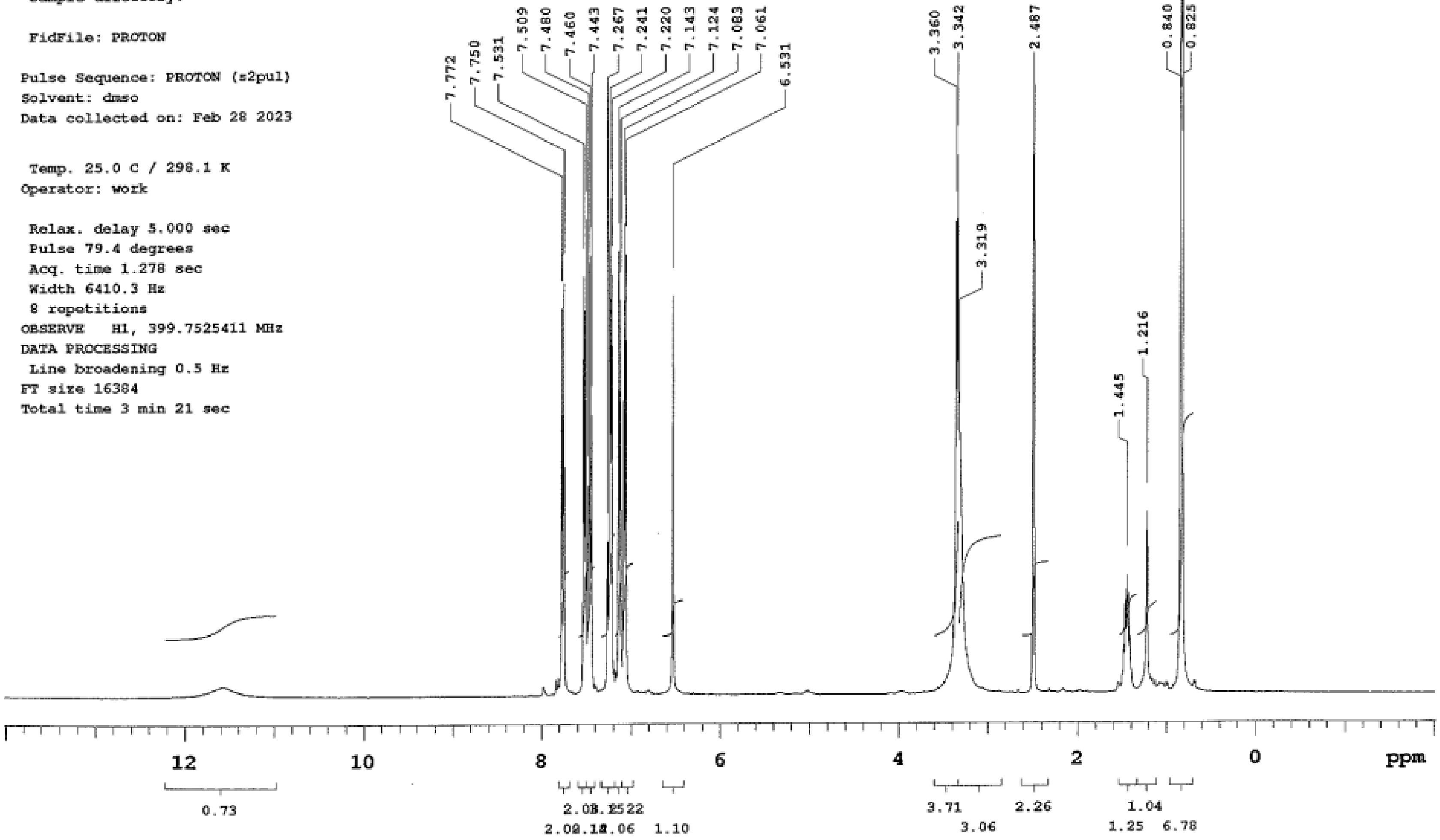

Compound 6

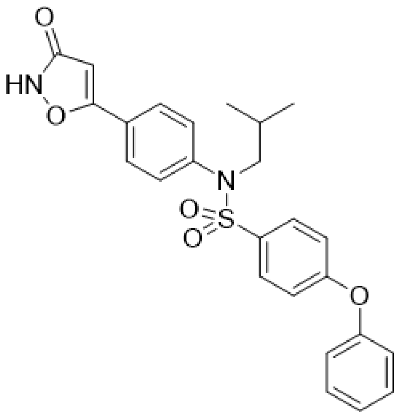

LC-5-069-C13

Sample Name:

Data Collected on:
400mr-vnmrs400
Archive directory:

Sample directory:

FidFile: CARBON

Pulse Sequence: CARBON (s2pul)
Solvent: dmso
Data collected on: Feb 21 2023

Temp. 25.0 C / 298.1 K
Operator: work

Relax. delay 5.000 sec
Pulse 67.7 degrees
Acq. time 0.813 sec
Width 21186.4 Hz
420 repetitions
OBSERVE C13, 100.5178564 MHz
DECOUPLE H1, 399.7545399 MHz
Power 39 dB
continuously on
WALTZ-16 modulated
DATA PROCESSING
Line broadening 3.[illegible] Hz
FT size 32768
Total time 12 hr, 5[illegible] min

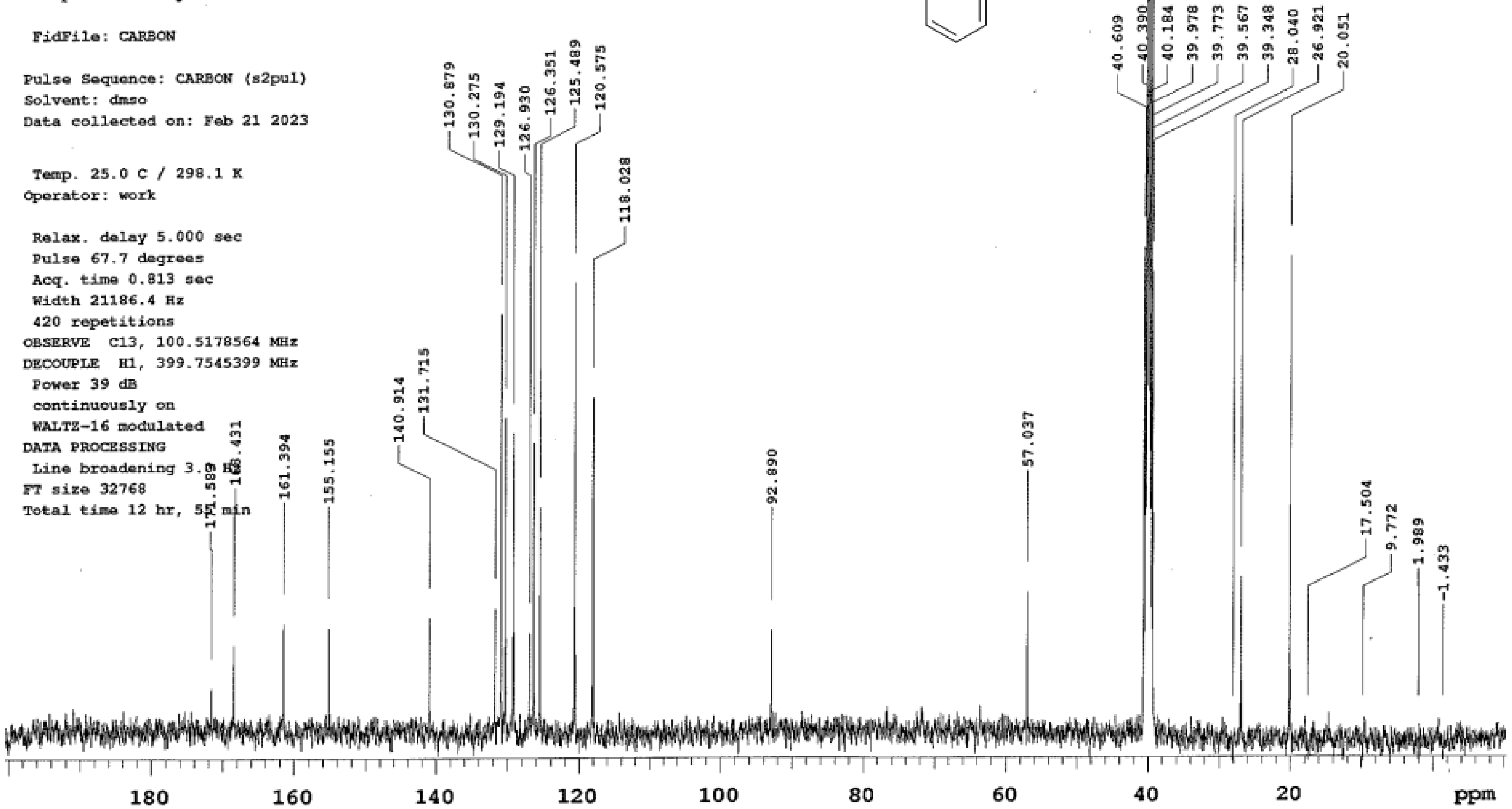

Compound 7a

LC-5-112-BDL-reprocess

Sample Name:

Data Collected on:
400mr-vnmrs400
Archive directory:

Sample directory:

FidFile: LC-5-112

Pulse Sequence: PROTON (s2pul)
Solvent: dmso
Data collected on: Jan 21 2016

Operator: work

Relax. delay 2.000 sec
Pulse 79.4 degrees
Acq. time 1.278 sec
Width 6410.3 Hz
4 repetitions
OBSERVE H1, 399.7531926 MHz
DATA PROCESSING
Line broadening 0.5 Hz
FT size 16384
Total time 0 min 13 sec

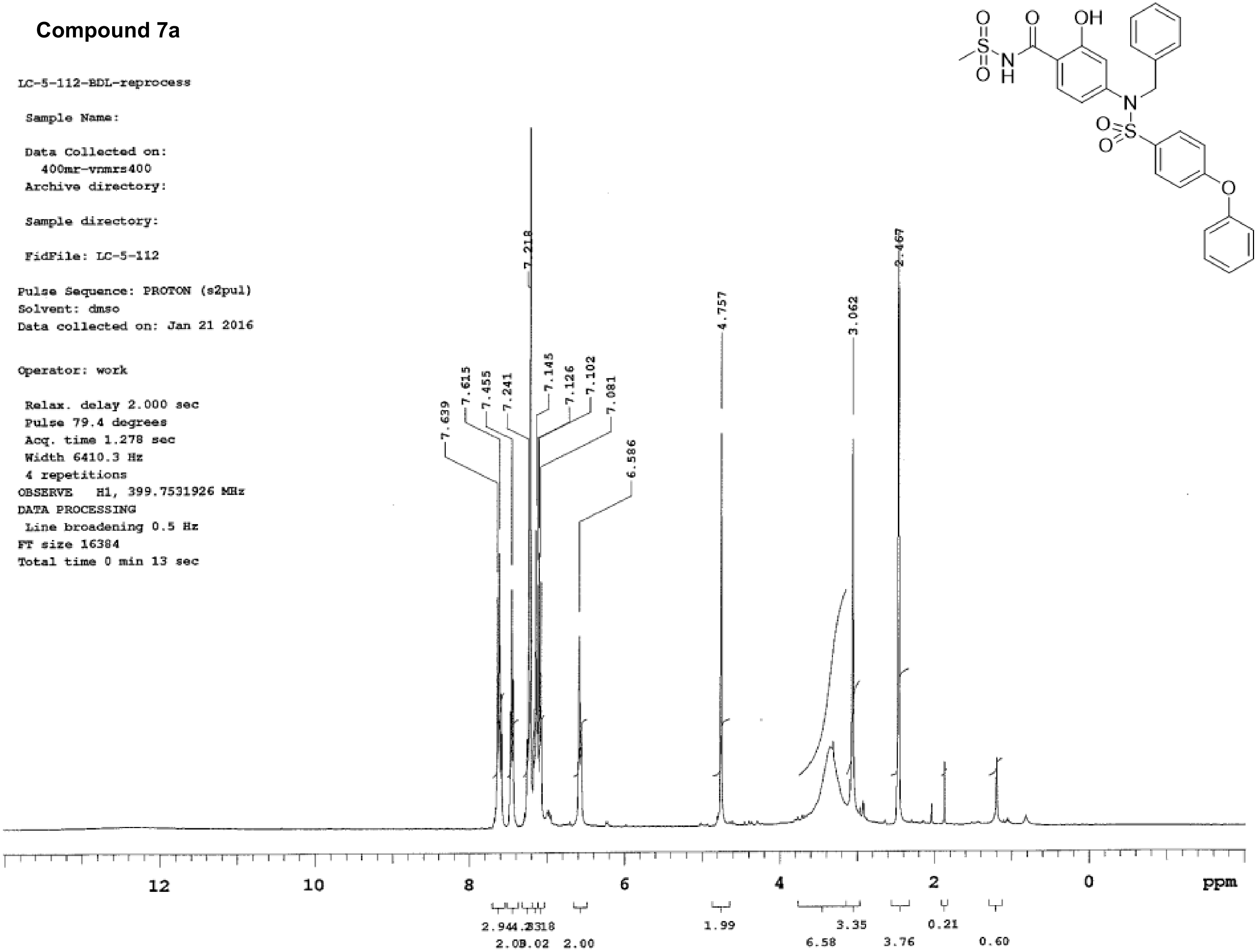

Compound 7b

LC-5-096/BDL-2-139

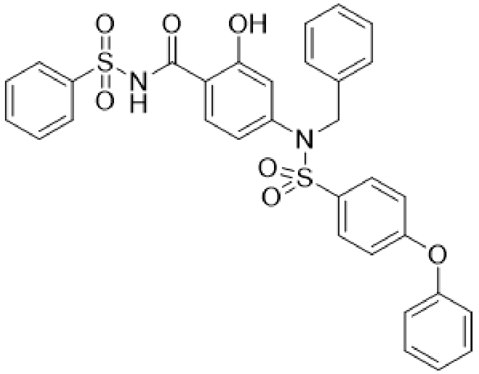

Sample Name:

Data Collected on:
400mr-vnmrs400
Archive directory:

Sample directory:

FidFile: PROTON

Pulse Sequence: PROTON (s2pul)
Solvent: dmso
Data collected on: Dec 28 2015

Operator: work

Relax. delay 3.000 sec
Pulse 79.4 degrees
Acq. time 1.278 sec
Width 6410.3 Hz
16 repetitions
OBSERVE H1, 399.7531926 MHz
DATA PROCESSING
Line broadening 0.5 Hz
FT size 16384
Total time 1 min 8 sec

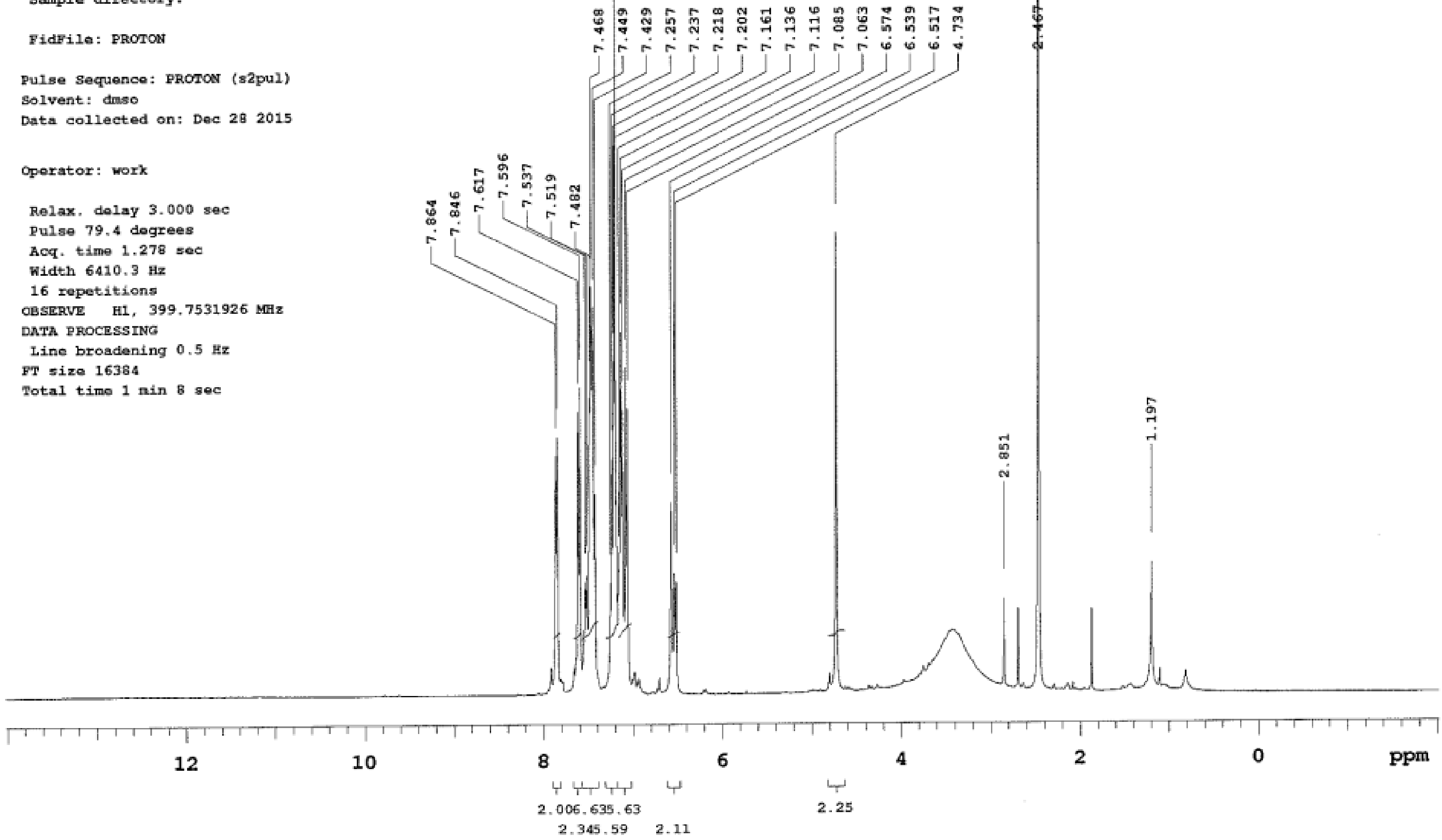

Compound 7b

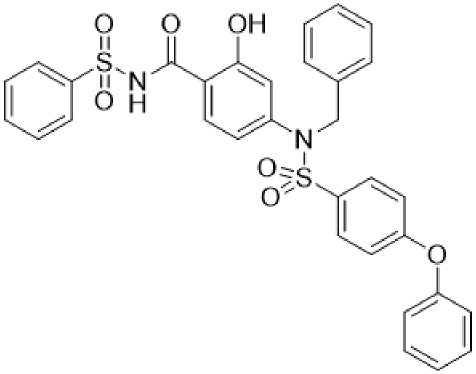

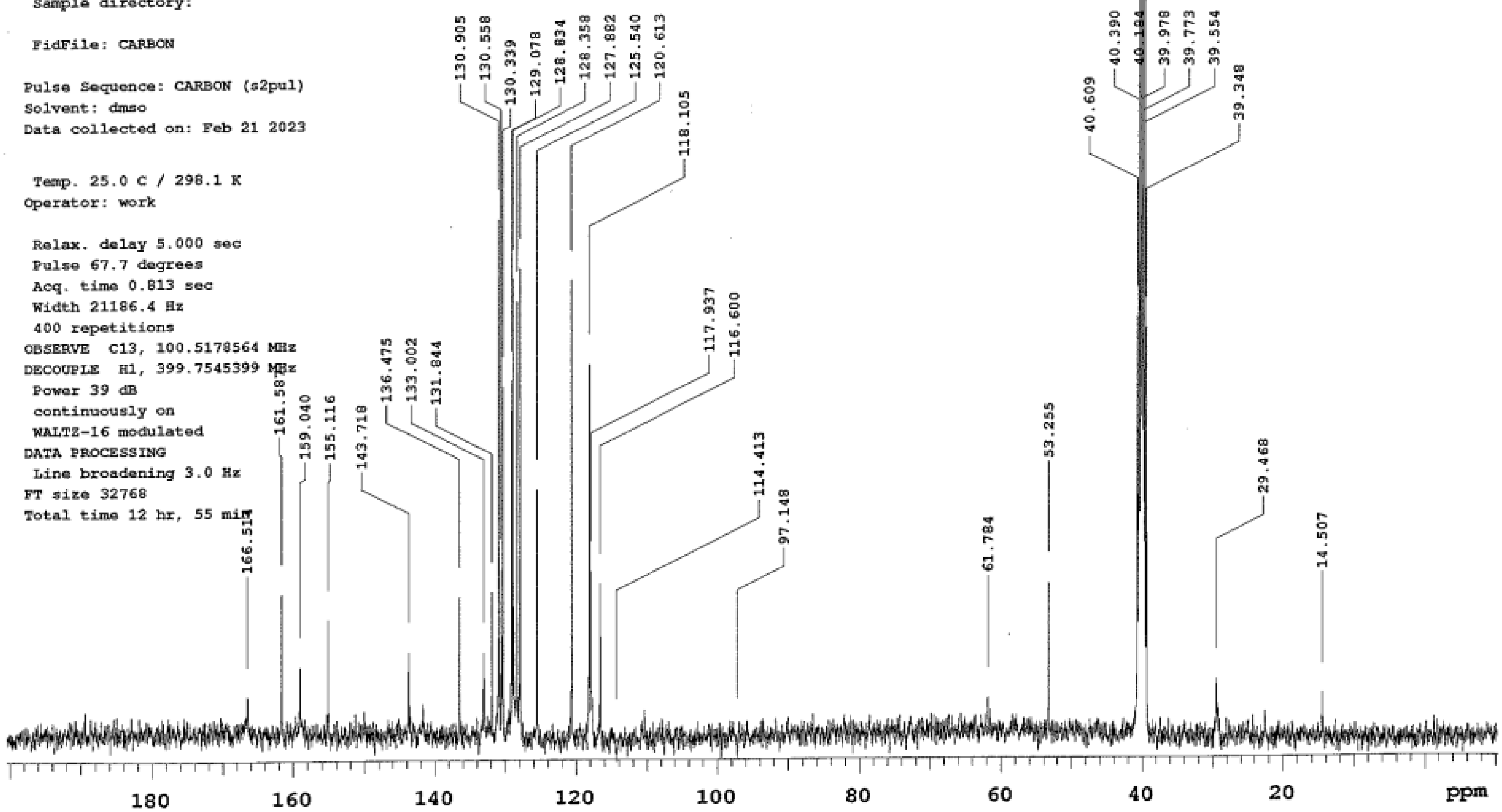
LC-5-096-C13
Sample Name:
Data Collected on:
400mr-vnmrs400
Archive directory:
Sample directory:
FidFile: CARBON
Pulse Sequence: CARBON (s2pul)
Solvent: dmso
Data collected on: Feb 21 2023
Temp. 25.0 C / 298.1 K
Operator: work
Relax. delay 5.000 sec
Pulse 67.7 degrees
Acq. time 0.813 sec
Width 21186.4 Hz
400 repetitions
OBSERVE C13, 100.5178564 MHz
DECOUPLE H1, 399.7545399 MHz
Power 39 dB
continuously on
WALTZ-16 modulated
DATA PROCESSING
Line broadening 3.0 Hz
FT size 32768
Total time 12 hr, 55 min
159.040
155.116
143.718
136.475
133.002
131.844
130.905
130.558
130.339
129.078
128.834
128.358
127.882
125.540
120.613
118.105
117.937
116.600
114.413
97.148
61.784
53.255
40.609
40.390
40.184
39.978
39.773
39.554
39.348
29.468
14.507
180
160
140
120
100
80
60
40
20
ppm

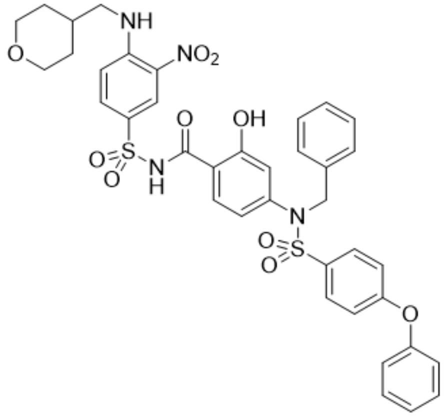

Compound 7c

LC-5-107-1H-BDL-rerun-reprocess

Sample Name:

Data Collected on:
400mr-vnmrs400
Archive directory:

Sample directory:

FidFile: LC-5-107-1H-BDL-rerun

Pulse Sequence: PROTON (s2pul)
Solvent: dmso
Data collected on: Feb 28 2023

Temp. 25.0 C / 298.1 K
Operator: work

Relax. delay 5.000 sec
Pulse 92.6 degrees
Acq. time 1.278 sec
Width 6410.3 Hz
4 repetitions
OBSERVE H1, 399.7525412 MHz
DATA PROCESSING
Line broadening 0.5 Hz
FT size 16384
Total time 3 min 21 sec

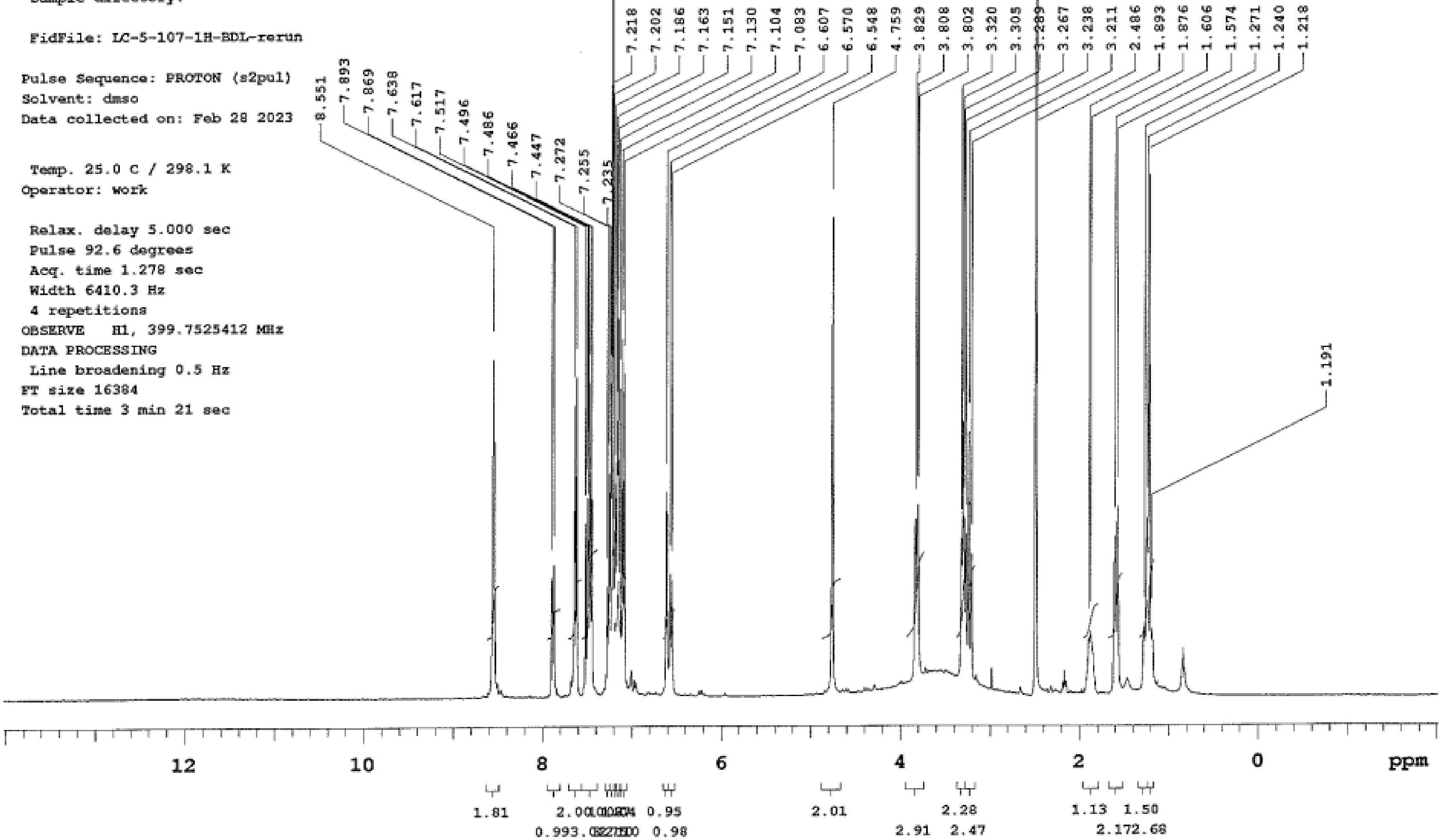

Compound 7c

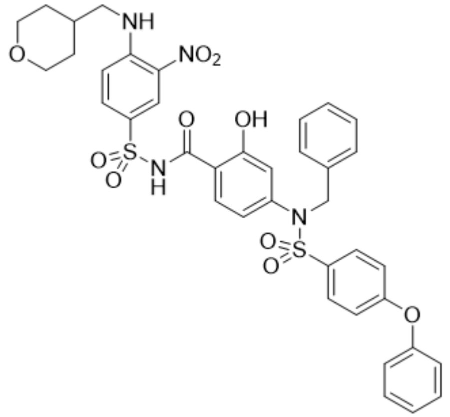

NH
NO2
O
OH
N
H
O=S=O
O

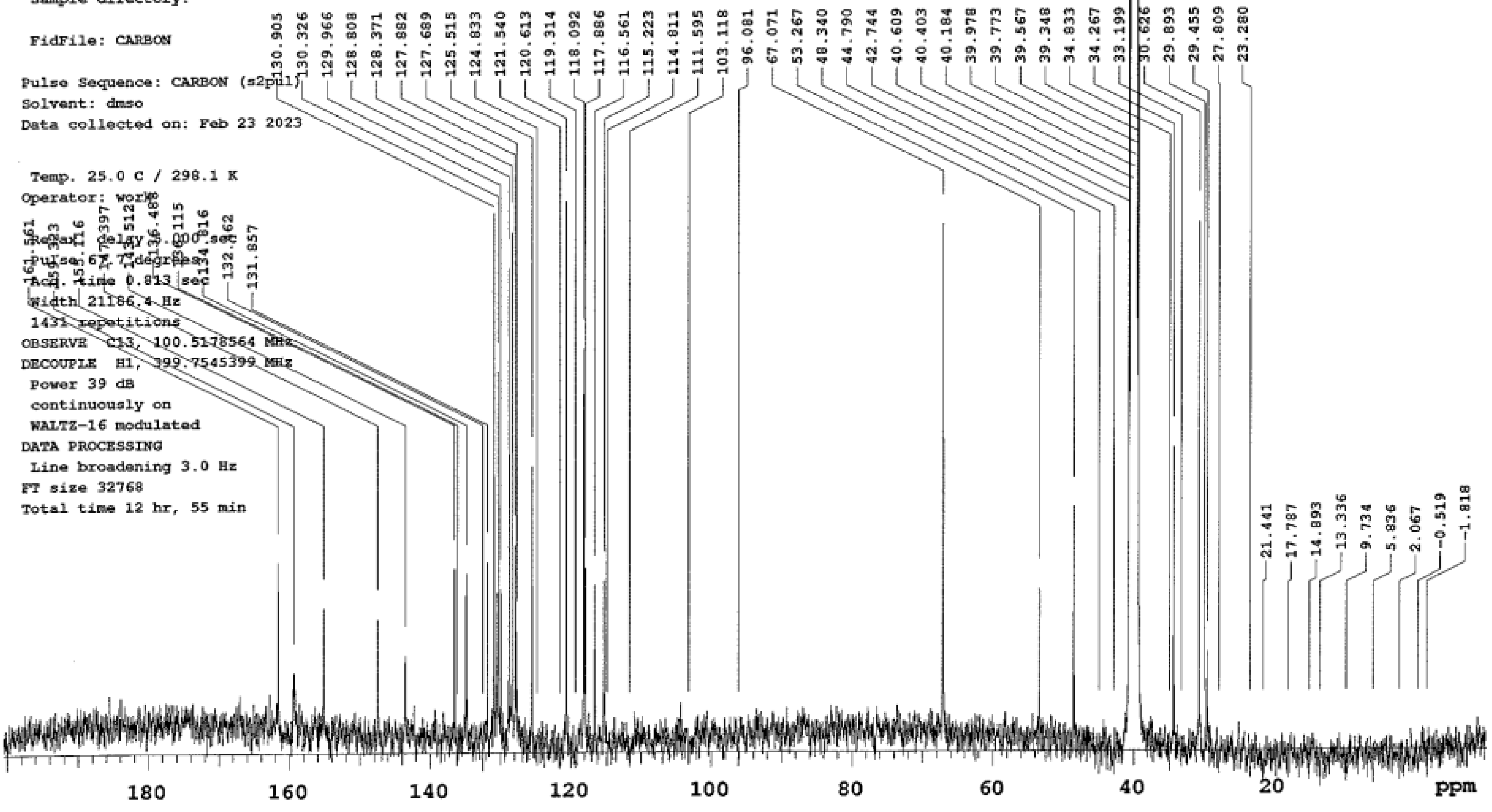

LC-5-107-C13
Sample Name:
Data Collected on:
400mr-vnmrs400
Archive directory:
Sample directory:
FidFile: CARBON
Pulse Sequence: CARBON (s2pul)
Solvent: dmso
Data collected on: Feb 23 2023
Temp. 25.0 C / 298.1 K
Operator: work
Acq. time 0.813 sec
Width 21186.4 Hz
1431 repetitions
OBSERVE C13, 100.5178564 MHz
DECOUPLE H1, 399.7545399 MHz
Power 39 dB
continuously on
WALTZ-16 modulated
DATA PROCESSING
Line broadening 3.0 Hz
FT size 32768
Total time 12 hr, 55 min
161.561
136.488
134.816
132.862
131.857
130.905
130.326
129.966
128.808
128.371
127.882
127.689
125.515
124.833
121.540
120.613
119.314
118.092
117.886
116.561
115.223
114.811
111.595
103.118
96.081
67.071
53.267
48.340
44.790
42.744
40.609
40.403
40.184
39.978
39.773
39.567
39.348
34.833
34.267
33.199
30.626
29.893
29.455
27.809
23.280
21.441
17.787
14.893
13.336
9.734
5.836
2.067
-0.519
-1.818
180
160
140
120
100
80
60
40
20
ppm

Compound 7d

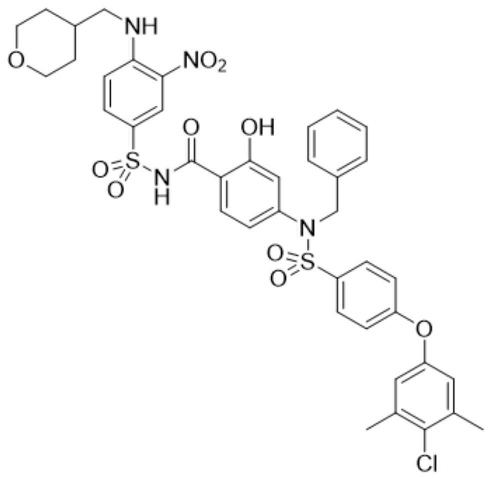

LC-5-131-1H-BDL-rerun-reprocess

Sample Name:

Data Collected on:
400mr-vnmrs400
Archive directory:

Sample directory:

FidFile: LC-5-131-1H-BDL-rerun

Pulse Sequence: PROTON (s2pul)
Solvent: dmso
Data collected on: Feb 28 2023

Temp. 25.0 C / 298.1 K
Operator: work

Relax. delay 5.000 sec
Pulse 92.6 degrees
Acq. time 1.278 sec
Width 6410.3 Hz
8 repetitions
OBSERVE H1, 399.7525412 MHz
DATA PROCESSING
Line broadening 0.5 Hz
FT size 16384
Total time 3 min 21 sec

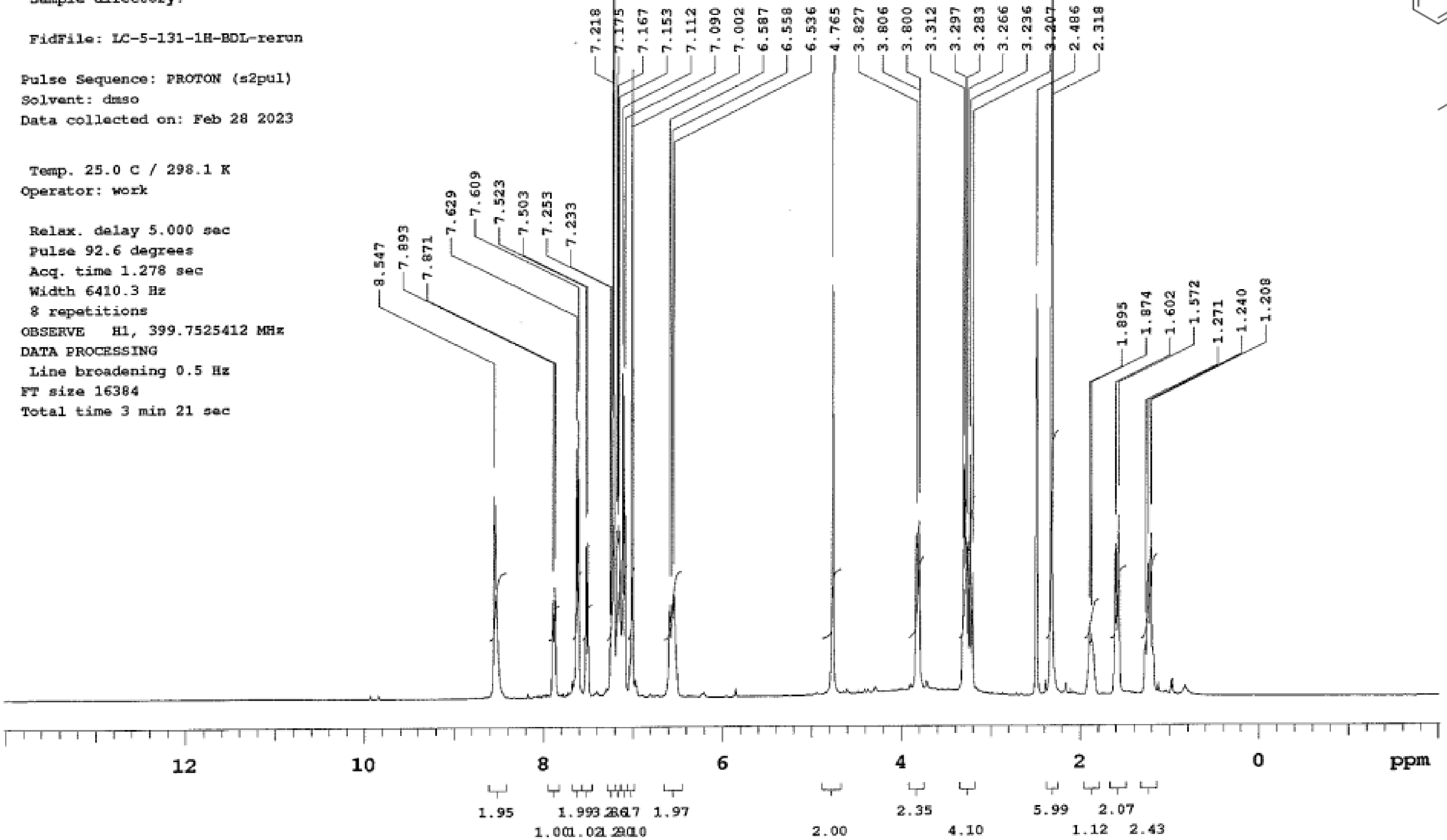

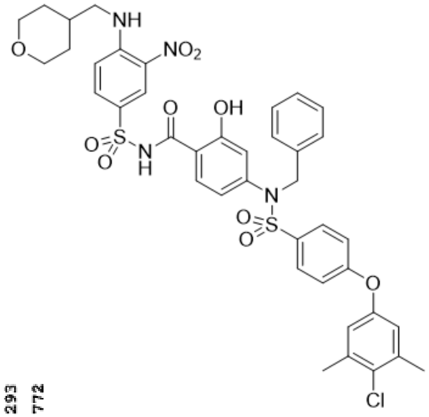

Compound 7d

LC-5-131-C13

Sample Name:

Data Collected on:
400mr-vnmrs400
Archive directory:

Sample directory:

FidFile: CARBON

Pulse Sequence: CARBON (s2pul)
Solvent: dmso
Data collected on: Feb 23 2023

Temp. 25.0 C / 298.1 K
Operator: work

Relax. delay 5.000 sec
Pulse 67.7 degrees
Acq. time 0.813 sec
Width 21186.4 Hz
601 repetitions
OBSERVE C13, 100.5178564 MHz
DECOUPLE H1, 399.7545399 MHz
Power 39 dB
continuously on
WALTZ-16 modulated
DATA PROCESSING
Line broadening 3.0 Hz
FT size 32768
Total time 12 hr, 55 min

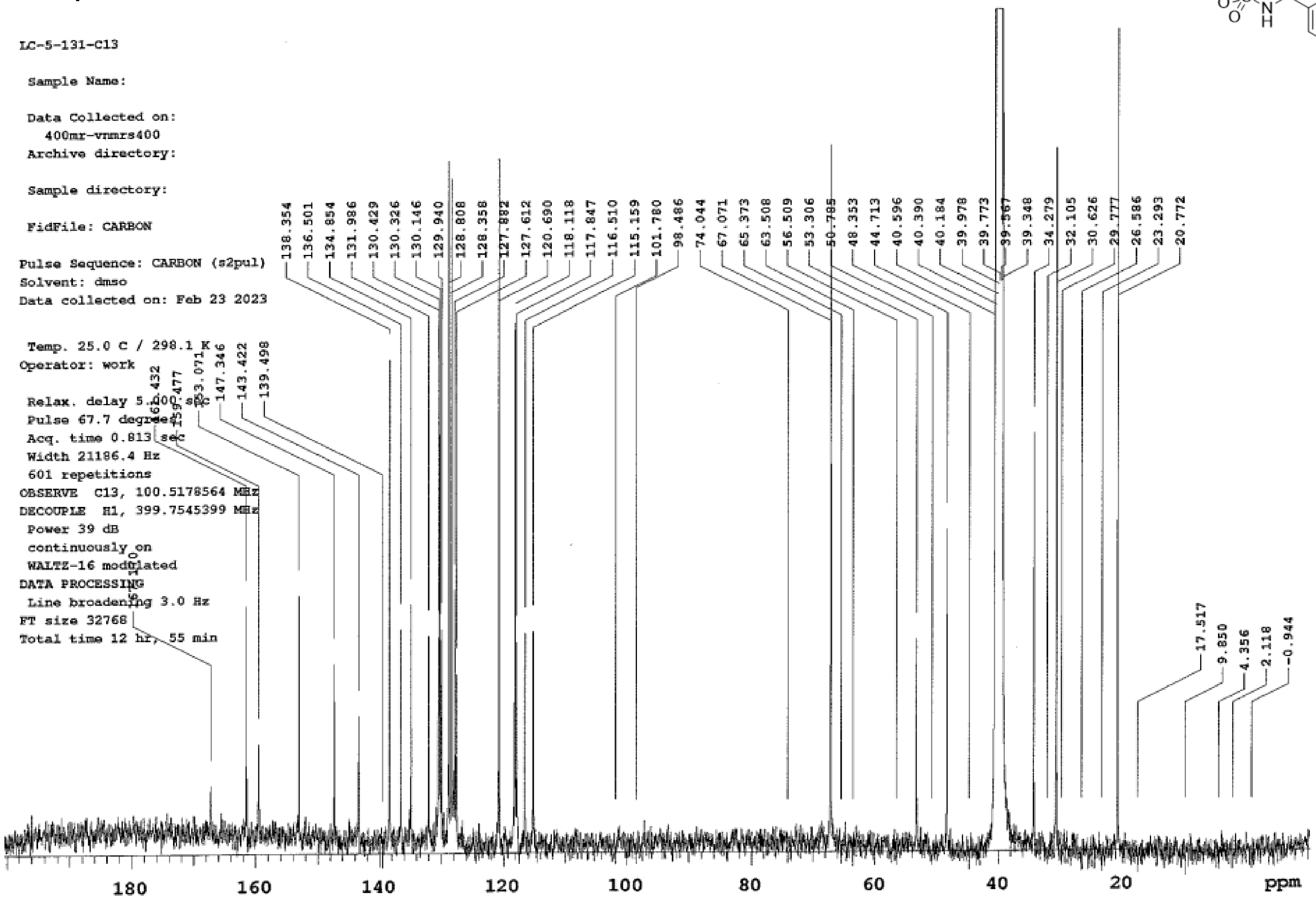

Compound 8

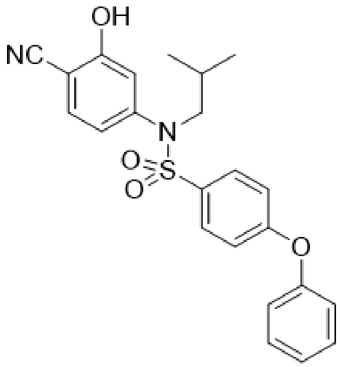

LC-5-050-1H-BDL-rerun

Sample Name:

Data Collected on:
400mr-vnmrs400
Archive directory:

Sample directory:

FidFile: PROTON

Pulse Sequence: PROTON (s2pul)
Solvent: dmso
Data collected on: Feb 28 2023

Temp. 25.0 C / 298.1 K
Operator: work

Relax. delay 5.000 sec
Pulse 92.6 degrees
Acq. time 1.278 sec
Width 6410.3 Hz
8 repetitions
OBSERVE H1, 399.7525412 MHz
DATA PROCESSING
Line broadening 0.5 Hz
FT size 16384
Total time 3 min 21 sec

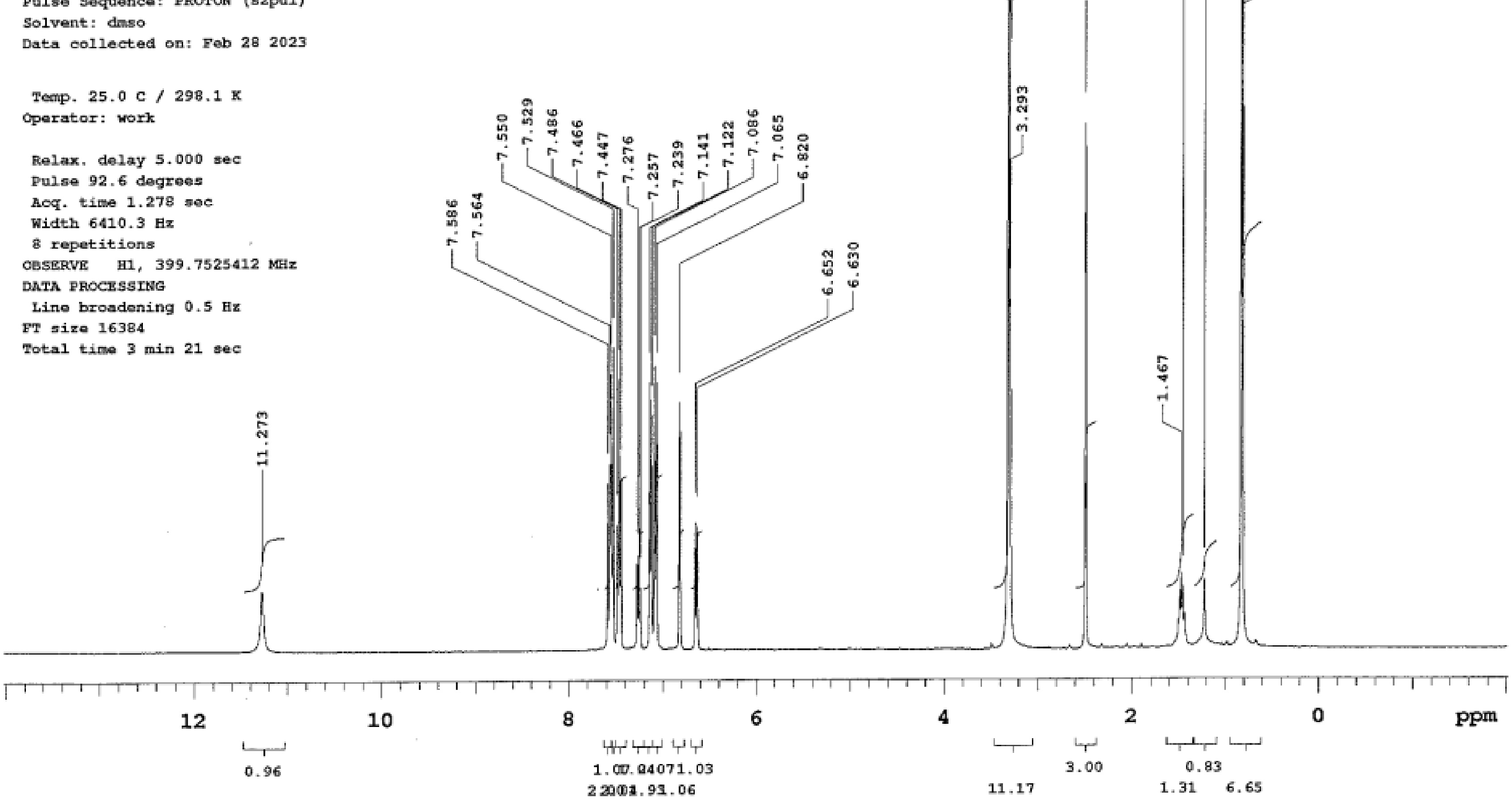

Compound 8

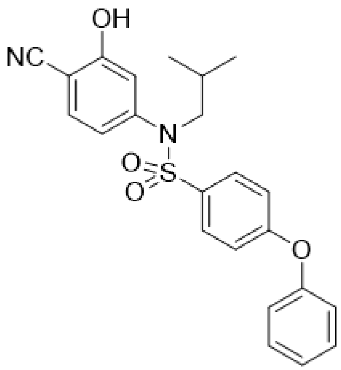

LC-5-050-C13

Sample Name:

Data Collected on:
400mr-vnmrs400
Archive directory:

Sample directory:

FidFile: CARBON

Pulse Sequence: CARBON (s2pul)
Solvent: dmso
Data collected on: Feb 21 2023

Temp. 25.0 C / 298.1 K
Operator: work

Relax. delay 5.000 sec
Pulse 67.7 degrees
Acq. time 0.813 sec
Width 21186.4 Hz
300 repetitions
OBSERVE C13, 100.5178564 MHz
DECOUPLE H1, 399.7545399 MHz
Power 39 dB
continuously on
WALTZ-16 modulated
DATA PROCESSING
Line broadening 3.0 Hz
FT size 32768
Total time 12 hr, 55 min

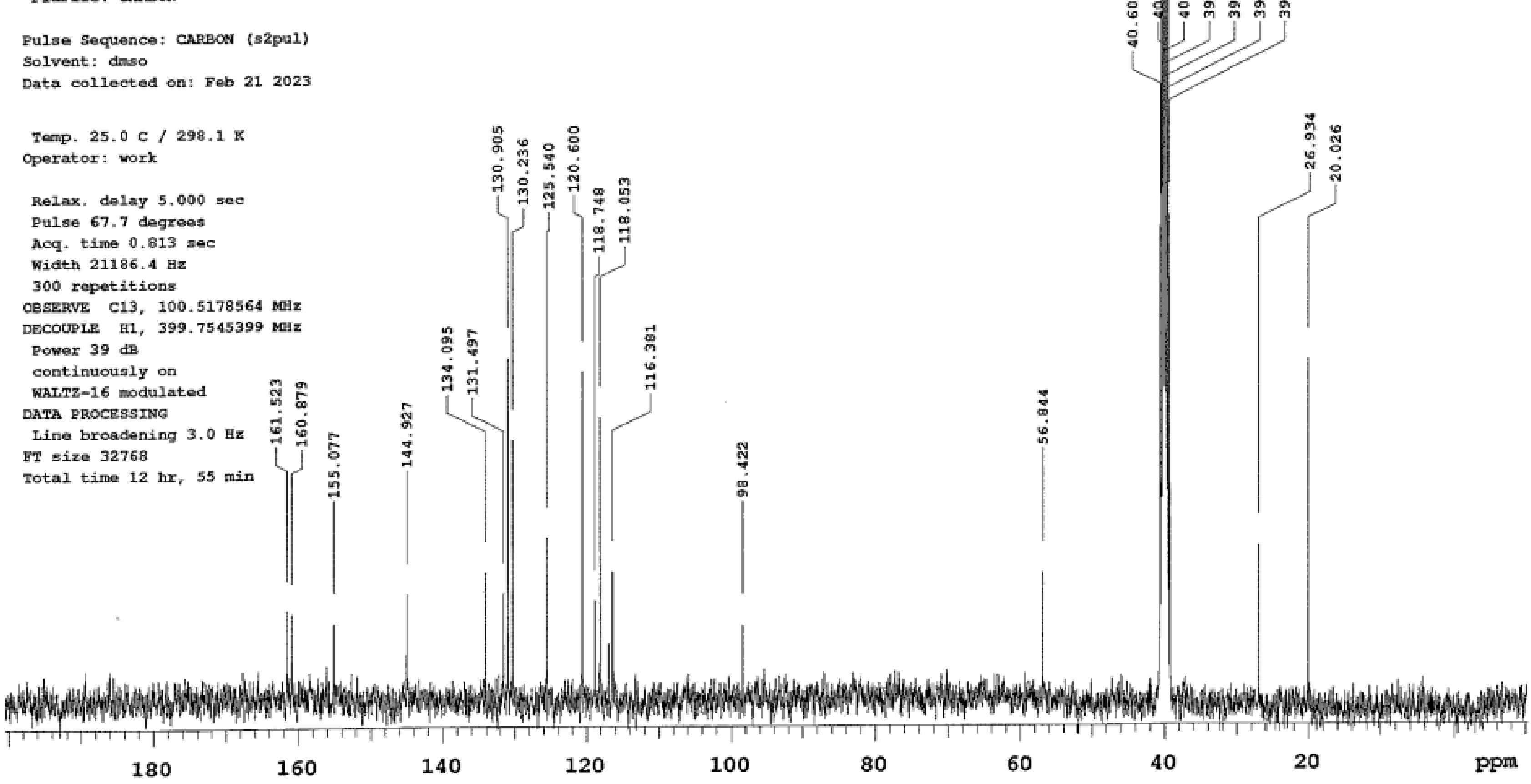

Compound 27

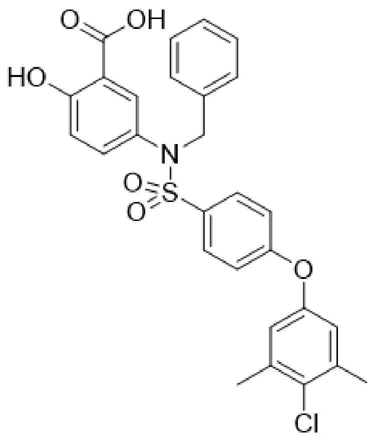

BDL-2-118-1H

Sample Name:

Data Collected on:
AgilentNMR-vnmrs500
Archive directory:
/home/vnmr1/vnmrsys/data/fidlib
Sample directory:

FidFile: BDL-2-118-1H

Pulse Sequence: PROTON (s2pul)
Solvent: dmso
Data collected on: Jan 6 2023

Temp. 25.0 C / 298.1 K
Operator: biopk

Relax. delay 5.000 sec
Pulse 93.6 degrees
Acq. time 1.304 sec
Width 6281.4 Hz
8 repetitions
OBSERVE H1, 499.7313913 MHz
DATA PROCESSING
Line broadening 0.5 Hz
FT size 16384
Total time 3 min 22 sec

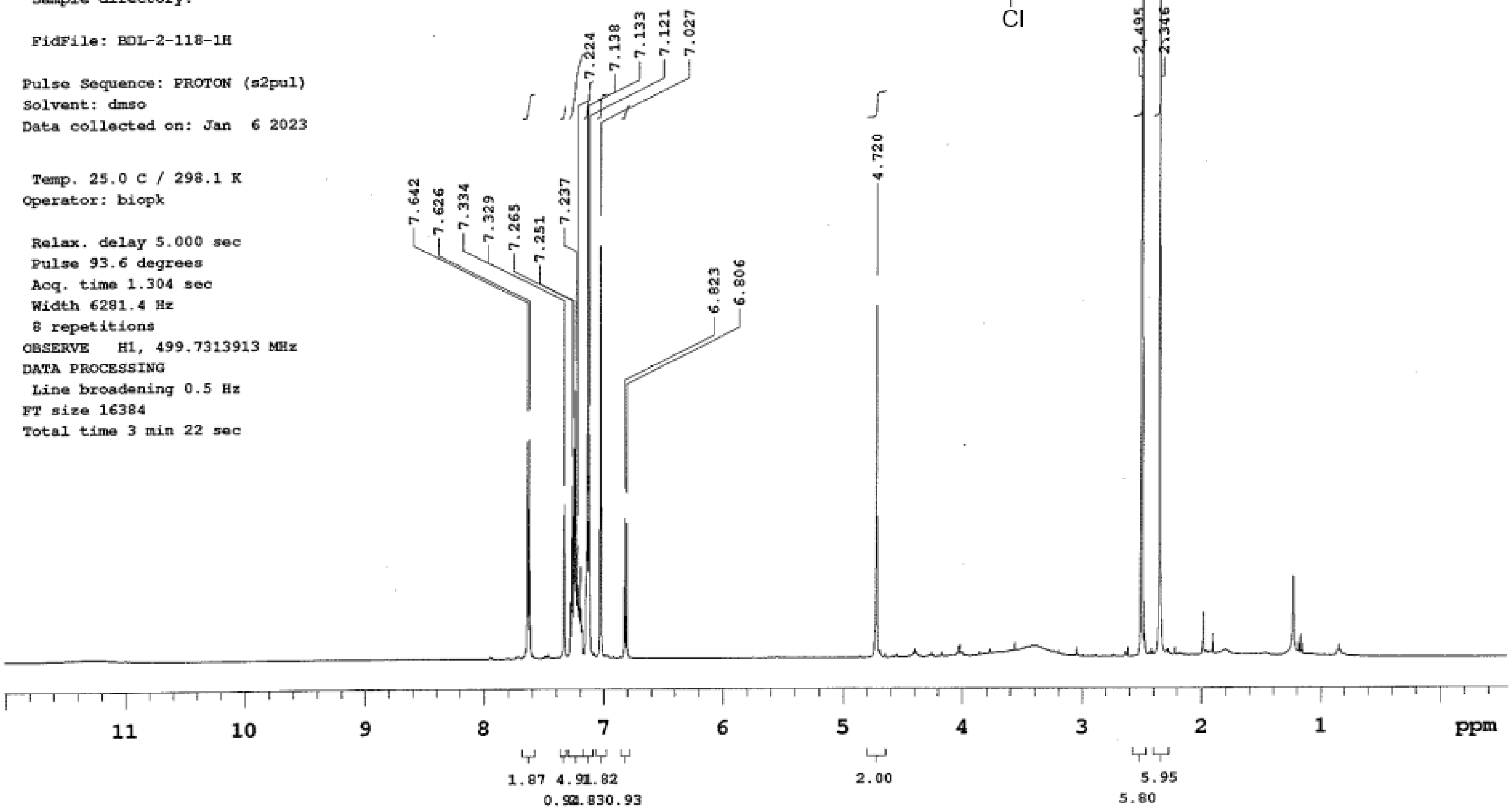

Compound 27

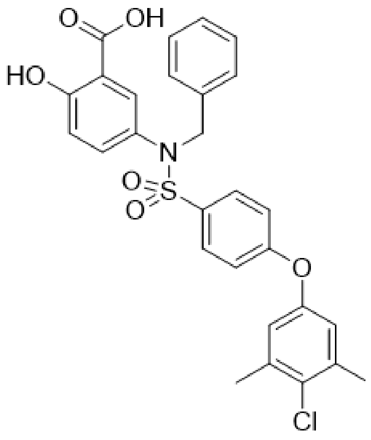

LC-5-129-BDL-2-118-C13

Sample Name:

Data Collected on:
400mr-vnmrs400
Archive directory:

Sample directory:

FidFile: CARBON

Pulse Sequence: CARBON (s2pul)
Solvent: dmso
Data collected on: Feb 23 2023

Temp. 25.0 C / 298.1 K
Operator: work

Relax. delay 5.000 sec
Pulse 67.7 degrees
Acq. time 0.813 sec
Width 21186.4 Hz
158 repetitions
OBSERVE C13, 100.5178564 MHz
DECOUPLE H1, 399.7545399 MHz
Power 39 dB
continuously on
WALTZ-16 modulated
DATA PROCESSING
Line broadening 3.0 Hz
FT size 32768
Total time 12 hr, 55 min

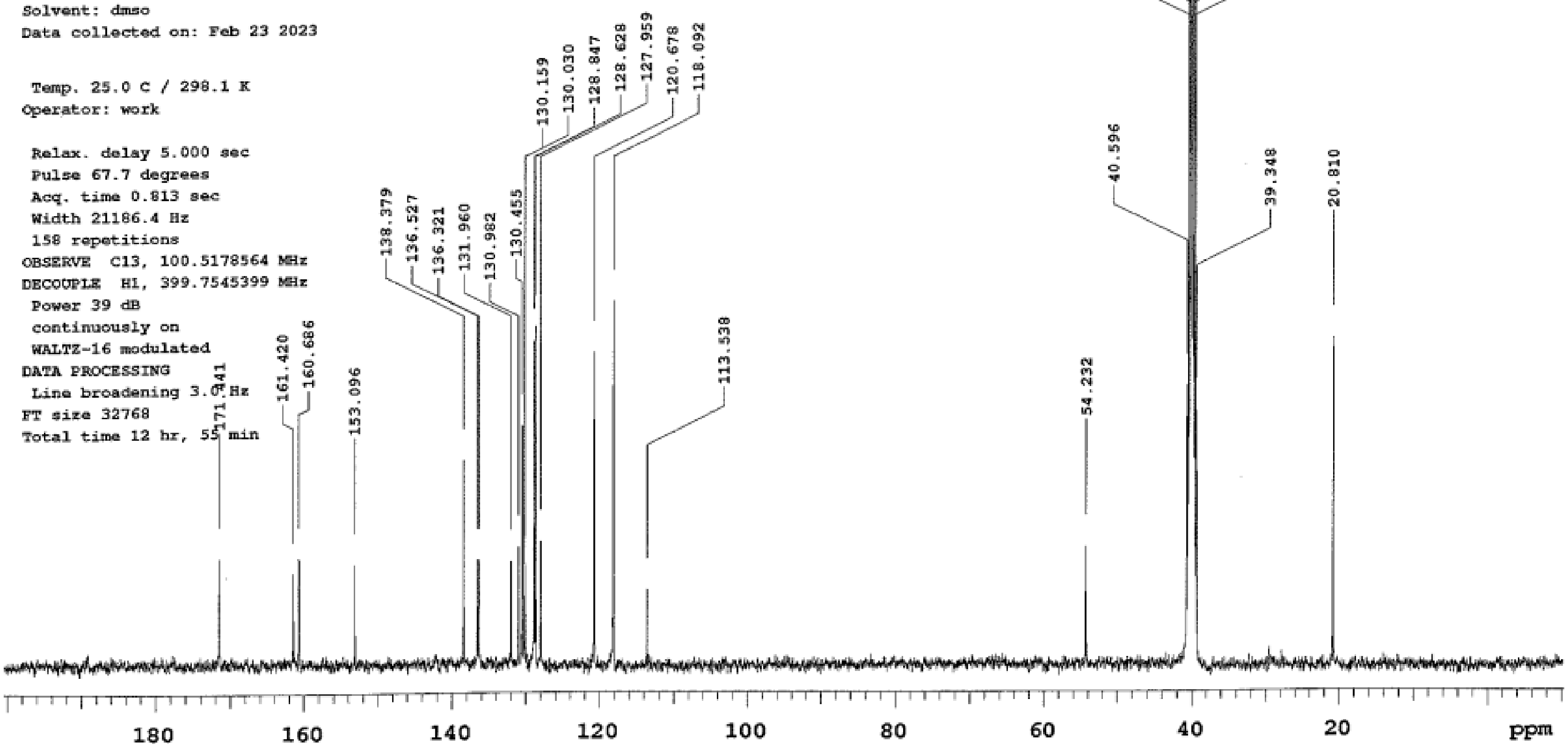

Compound 29

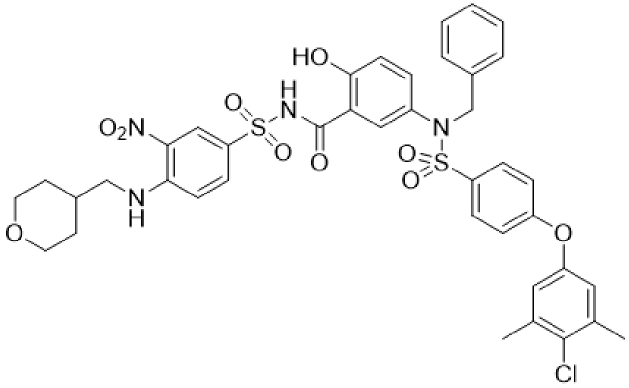

LC-5-132-1H-BDL-rerun

Sample Name:

Data Collected on:
400mr-vnmrs400
Archive directory:

Sample directory:

FidFile: PROTON

Pulse Sequence: PROTON (s2pul)
Solvent: dmso
Data collected on: Feb 28 2023

Temp. 25.0 C / 298.1 K
Operator: work

Relax. delay 5.000 sec
Pulse 92.6 degrees
Acq. time 1.278 sec
Width 6410.3 Hz
8 repetitions
OBSERVE H1, 399.7525412 MHz
DATA PROCESSING
Line broadening 0.5 Hz
FT size 16384
Total time 3 min 21 sec

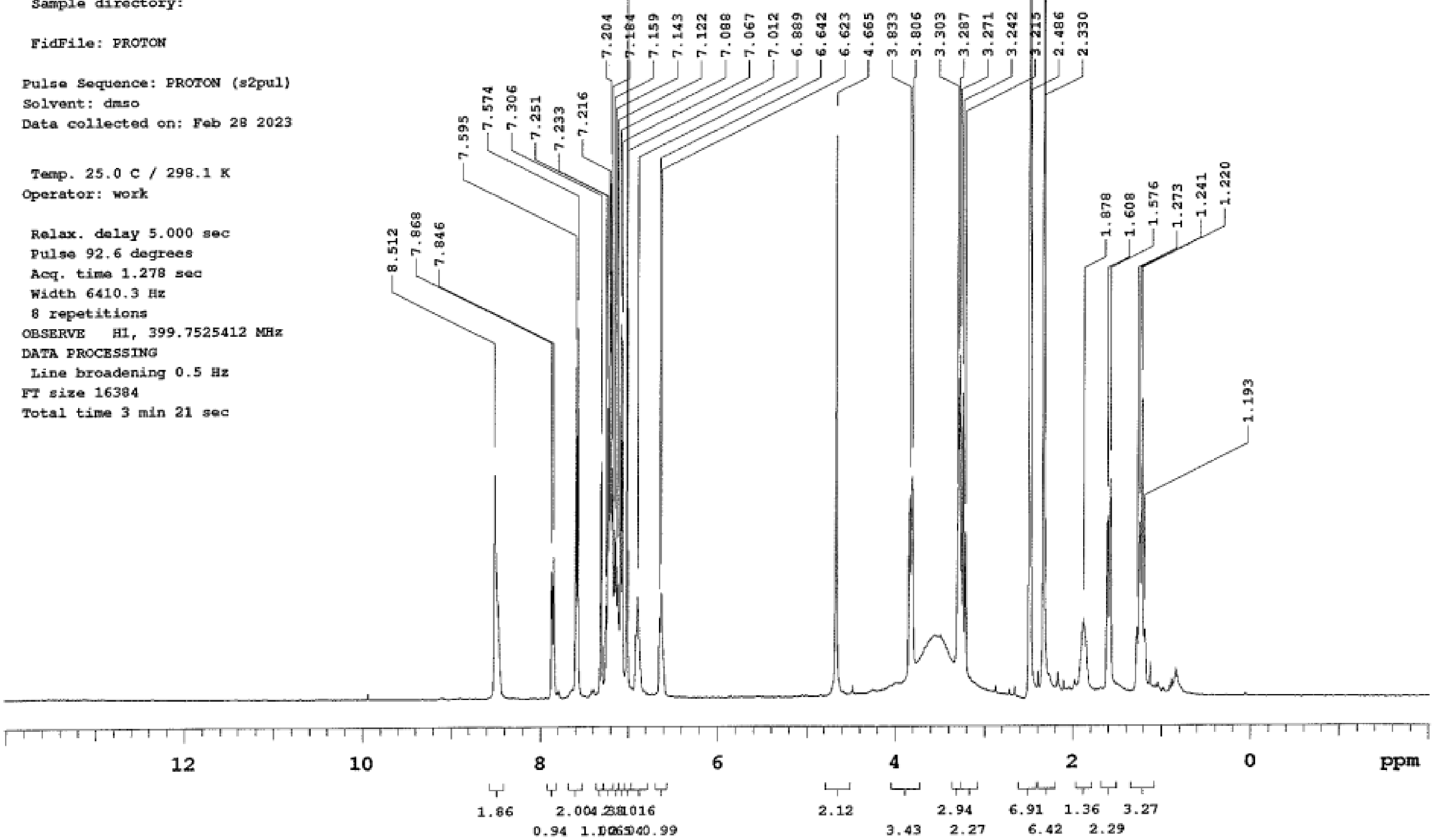

Compound 29

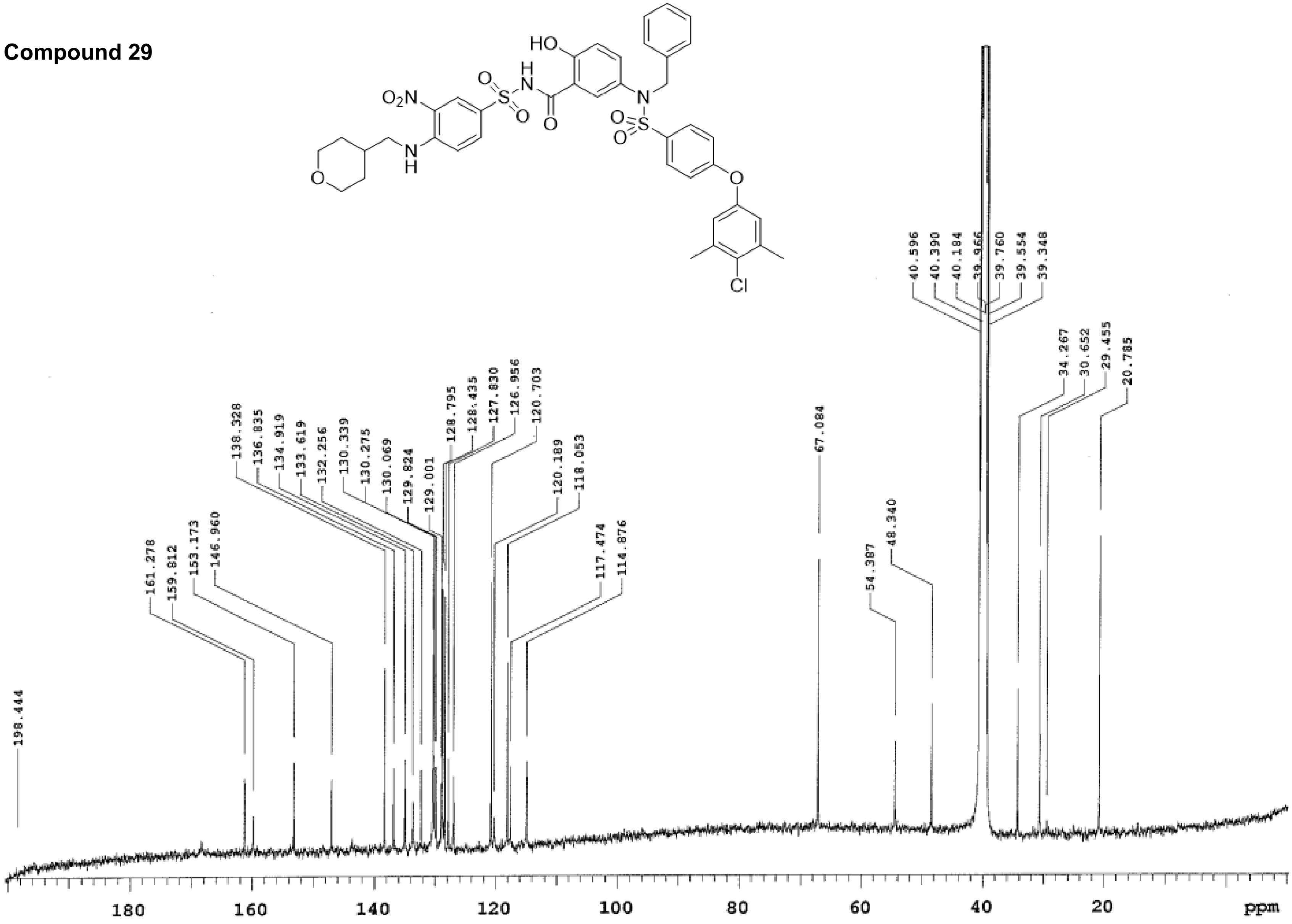

HO
O2N
NH
Cl
198.444
161.278
159.812
153.173
146.960
138.328
136.835
134.919
133.619
132.256
130.339
130.275
130.069
129.824
129.001
128.795
128.435
127.830
126.956
120.703
120.189
118.053
117.474
114.876
67.084
54.387
48.340
40.596
40.390
40.184
39.966
39.760
39.554
39.348
34.267
30.652
29.455
20.785
180
160
140
120
100
80
60
40
20
ppm
